# A Survey of Small‐Scale Waves and Wave‐Like Phenomena in Jupiter's Atmosphere Detected by JunoCam

**DOI:** 10.1029/2019JE006369

**Published:** 2020-06-28

**Authors:** Glenn S. Orton, Fachreddin Tabataba‐Vakili, Gerald Eichstädt, John Rogers, Candice J. Hansen, Thomas W. Momary, Andrew P. Ingersoll, Shawn Brueshaber, Michael H. Wong, Amy A. Simon, Leigh N. Fletcher, Michael Ravine, Michael Caplinger, Dakota Smith, Scott J. Bolton, Steven M. Levin, James A. Sinclair, Chloe Thepenier, Hamish Nicholson, Abigail Anthony

**Affiliations:** ^1^ Jet Propulsion Laboratory California Institute of Technology Pasadena CA USA; ^2^ Independent Scholar Stuttgart Germany; ^3^ British Astronomical Association London UK; ^4^ Planetary Science Institute Tucson AZ USA; ^5^ Division of Geological and Planetary Sciences, California Institute of Technology Pasadena CA USA; ^6^ College of Engineering and Applied Sciences, Western Michigan University Kalamazoo MI USA; ^7^ Department of Astronomy, University of California Berkeley CA USA; ^8^ SETI Institute Mountain View CA USA; ^9^ NASA Goddard Space Flight Center Greenbelt MD USA; ^10^ Department of Physics and Astronomy, University of Leicester Leicester UK; ^11^ Malin Space Science Systems San Diego CA USA; ^12^ National Center for Atmospheric Research Boulder CO USA; ^13^ Space Science and Engineering Division, Southwest Research Institute San Antonio TX USA; ^14^ Glendale Community College Glendale CA USA; ^15^ Now at the University of California Davis CA USA; ^16^ Harvard College Cambridge MA USA; ^17^ Golden West College Huntington Beach CA USA; ^18^ Now at the University of California Berkeley CA USA

**Keywords:** Jupiter, atmosphere, waves, Juno, JunoCam, dynamics

## Abstract

In the first 20 orbits of the Juno spacecraft around Jupiter, we have identified a variety of wave‐like features in images made by its public‐outreach camera, JunoCam. Because of Juno's unprecedented and repeated proximity to Jupiter's cloud tops during its close approaches, JunoCam has detected more wave structures than any previous surveys. Most of the waves appear in long wave packets, oriented east‐west and populated by narrow wave crests. Spacing between crests were measured as small as ~30 km, shorter than any previously measured. Some waves are associated with atmospheric features, but others are not ostensibly associated with any visible cloud phenomena and thus may be generated by dynamical forcing below the visible cloud tops. Some waves also appear to be converging, and others appear to be overlapping, possibly at different atmospheric levels. Another type of wave has a series of fronts that appear to be radiating outward from the center of a cyclone. Most of these waves appear within 5° of latitude from the equator, but we have detected waves covering planetocentric latitudes between 20°S and 45°N. The great majority of the waves appear in regions associated with prograde motions of the mean zonal flow. Juno was unable to measure the velocity of wave features to diagnose the wave types due to its close and rapid flybys. However, both by our own upper limits on wave motions and by analogy with previous measurements, we expect that the waves JunoCam detected near the equator are inertia‐gravity waves.

## Key Points

- In the first 20 orbits of the Juno mission, over 150 waves and wave‐like features have been detected by the JunoCam public‐outreach camera
- A wide variety of wave morphologies were detected over a wide latitude range, but the great majority were found near Jupiter's equator
- By analogy with previous studies of waves in Jupiter's atmosphere, most of the waves detected are likely to be inertia‐gravity waves

## 1 Introduction

The Juno mission's JunoCam instrument (Hansen et al., 2017), conceived as a public‐outreach camera, has provided a surprising wealth of scientific results. These include the first close‐up examination of Jupiter's polar regions (Orton et al., 2017), in particular, the unexpected presence and properties of constellations of cyclonic vortices around each pole (Adriani et al., 2018; Tabataba‐Vakili et al., 2020). JunoCam's proximity to Jupiter's cloud tops has also provided high‐resolution details of Jupiter's Great Red Spot and its environment (Sánchez‐Lavega et al., 2018). These studies have been enabled by JunoCam's wide field of view (58°) and the close proximity of the spacecraft to the clouds being imaged, with target distances as small as 3,500 km near closest approaches (“perijoves”), yielding a horizontal pixel‐to‐pixel spacing as good as 3 km.

We have used JunoCam's coverage over a wide range of latitudes, coupled with its high spatial resolving power, to examine all of our images for various phenomena in Jupiter's clouds. Small‐scale waves, with wavelengths (distances between wave crests) less than ~300 km, were first detected in 1979 by Voyager (Hunt & Muller, 1979) and have been detected by Galileo (e.g., Bosak & Ingersoll, 2002) and New Horizons (e.g., Reuter et al., 2007) since then, as well as by the near‐infrared JIRAM instrument on Juno (Adriani et al., 2018; Fletcher et al., 2018). Larger waves, with scales of 1,200 km or greater, have since also been detected from the Earth using Hubble Space Telescope (HST) and ground‐based imaging (Simon et al., 2018). A summary of observations of these waves is given in Table 1, which includes and updates similar information in Table 1 of Simon et al. (2015) and various tables in Simon et al. (2018). Table 1 includes a JunoCam wave feature examined by Sánchez‐Lavega et al. (2018) that we will also consider in this report. No waves were detected by the Cassini mission, most likely because Cassini was too far from Jupiter for adequate spatial resolution, but other reasons are possible. Virtually none were seen by Galileo imaging despite several close, although spatially limited, passes. The planet‐encircling New Horizons waves were a surprise, as were the larger waves observed by HST and ground‐based imaging for the past 4 years, which Cassini would have detected. During the Cassini epoch, there may not have been sufficient contrast to detect waves or waves were simply not propagating because of conditions unknown.

**Table 1:** Summary of Previous Observations of Waves in Jupiter's Clouds Detected at 5 μm or Shorter Wavelengths That Include Small‐Scale Waves, That Is, Those Shorter Than 1,000 km

Observing platform (year)	Associated publications	Range of planetocentric latitudes	Range of wavelengths (km)
Voyager (1979)	Hunt and Muller (1979), Flasar and Gierasch (1986)	27^°^S to 27^°^N	70–430
Galileo (1996)	Bosak and Ingersoll (2002)	13^°^S	300
Galileo (1999)	Arregi et al. (2009), Simon, Li, and Reuter (2015)	0.2^°^N, 3.6^°^N	155–205
Galileo (2001)	Arregi et al. (2009)	1.8^°^S	195–215
New Horizons (2007)	Reuter et al. (2007), Simon, Li, and Reuter (2015)	0^°^ to 1.1^°^N	280–330
Juno/JIRAM (2017)	Adriani, Moriconi, et al. (2018), Fletcher et al. (2018)	14^°^ to 15^°^N	1,400–1,900
Juno/JunoCam (2017)	Sánchez‐Lavega et al. (2018)	16^°^S	35
Hubble Space Telescope (2012–2018)	Simon et al. (2018)	14.5^°^ ± 2.5^°^N	1,220–1,340
Ground‐Based Visible Observations (2017)	Simon et al. (2018)	14.5^°^ ± 2.5^°^N	1,220–1,340
Ground‐Based 5‐μm Observations (2016–2017)	Fletcher et al. (2018)	14.5^°^ ± 2.5^°^N	1,300–1,600

*Note*. Some values are also displayed in Figure 16. The waves addressed by Sánchez‐Lavega et al. (2018) are associated with the Great Red Spot.

Below, we describe how the measurements are made, followed by a survey of the different types of atmospheric waves we have detected—along with any analogous wave formations in the Earth's atmosphere. We then discuss quantitative properties of the waves and conclude with an analysis and discussion section.

## 2 Description of the Measurements

JunoCam is a CCD‐based camera, spanning a 58° field of view. The instrument is a “push‐frame” imager, taking advantage of Juno's 2 RPM spin to sweep its 58° swath to build spatial and spectral coverage without involving a shuttering mechanism. Thus, sequential images are acquired in broadband blue, green, and red filters plus a narrow band filter centered on a 889‐nm methane absorption band. Time‐delayed integration of multiple pixel rows builds up the signal‐to‐noise ratio. Hansen et al. (2017) provide details of the instrument and its modes of operation. Sequential images are typically rendered in red‐green‐blue (“RGB”) composites, with the “methane filter” acquired and rendered separately, and the RGB images cover all latitudes on nearly all perijoves. The spatial resolution varies with the distance to the planet, which changes with each orbit: Successive perijoves move approximately one degree of latitude north. For all the waves we discuss in this report, the spatial resolution is much finer than the distances reported in each case.

In order to determine properties of the features, each image was transformed into a cylindrical cartesian map in longitude and latitude. This was done independently of the standard coordinate‐transformation approach using the SPICE system (Acton, 1996), as image timing, orientation in the spacecraft coordinate system, and optics distortion were still being determined. We used limb fitting to constrain these properties, as the limb appears in all of our images. Current SPICE data show good agreement with these maps, with the limb‐fitting approach showing an uncertainty better than 2° in the position of the south pole, as reported by Tabataba‐Vakili et al. (2020). Further details of this mapping process are provided by Adriani, Mura, et al. (2018) (see their Supplementary Information) and by Tabataba‐Vakili et al. (2020). All JunoCam images are publicly available on the Mission Juno web site: https://www.missionjuno.swri.edu/junocam/processing.

**Figure 1:**
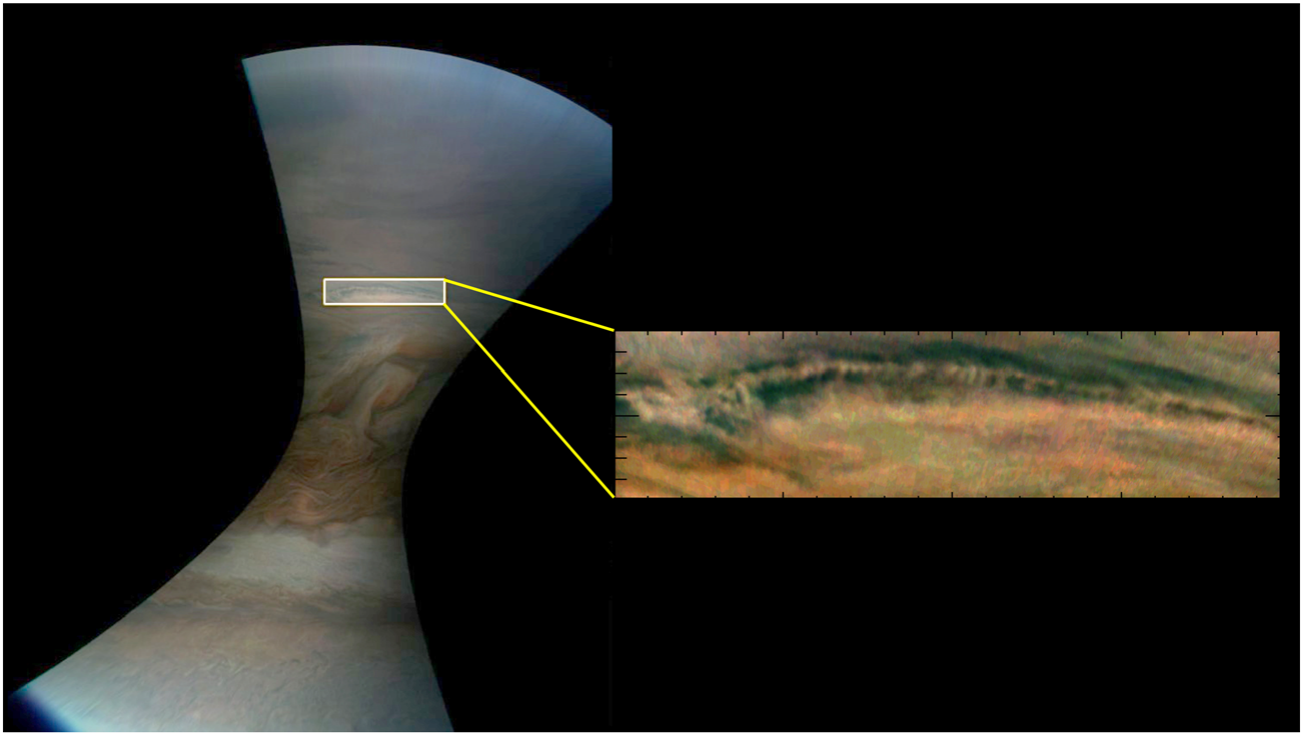
Example of excerpting a portion of a wave‐like feature from a JunoCam image from planetocentric latitudes 3°S to 4°S. This is a cylindrical mapping of a color‐composited image JNCE_2017297_09C00088_V01 (which here and elsewhere we simply identify as Image 88 from PJ9. The extracted panel is also shown as Figure 10b and in the supporting information as Figure PJ09_88 with a grid indicating the location of peak radiances in the wave‐like feature.

Figure 1 shows an example of a full JunoCam image, rendered in a cylindrically mapped format, together with an excerpt (“crop”) of the image in which we identify wave‐like features. The mapped versions were adjusted to compensate for the variation of illumination across the field. We found that a second‐order power‐law enhancement of color composites allowed wave features to be identified more readily. For the images shown below, as well as in the supporting information, we further stretched each red, green, and blue colors independently for ease of identification by the reader. We also applied unsharp‐mask sharpening in a few cases to make faint waves appear more prominently. Several coauthors independently searched manually through all of the JunoCam images in order to identify wave‐like features that were candidates for this study. For detailed quantitative measurements, we used additional high‐pass filtering to isolate fine‐scale features. Our quantitative measurements are based on maps of the images rendered with 180 pixels per degree of latitude and longitude, together with high‐pass filtering. We did not find identifiable wave features in any methane‐band images. As a result, our discussion will be limited to enhanced RGB‐composite images. We did not see any consistent differences in the contrast of wave features between the colors in images, which do not have any radiometric calibration.

## 3 Results

### 3.1 Overview

We limited the search for and characterization of waves to observations between perijoves 1 and 20 (27 August 2016 to 29 May 2019). Hereafter we will abbreviate “perijove” as “PJ.” During PJ2 (19 October 2016), no close‐up images were made of Jupiter's atmosphere as the result of a spacecraft “safing” event immediately before close approach. During PJ19 (6 April 2019), JunoCam only took distant images of Jupiter, as a result of an unusual orientation of the spacecraft for most of that perijove in order to enable scanning in longitude by Juno's Microwave Radiometer (MWR) instrument. The supporting information to this report documents and illustrates all of the images in which we identified wave‐like features with more than two wavefronts, together with a visual aid to identify the waves. In this report we select particular images that provide examples of the wide variety of waves and wave‐like phenomena and their properties. The reader is free to observe all the images that are available in various processed forms on the Mission Juno web site in order to verify or refute our selections, as well as to identify potential additional candidates. We define “small‐scale” as waves less than 1,000 km, although less than a dozen of the features we identified out of the total of 157 (Table 2) have wavelengths larger than 400 km.

**Table 2:** Number of Features in Each Morphological Category, Listed in Order of Frequency

Type of wave‐like feature (section where discussed)	Number of features
Long packets, short crests (3.2.1)	100
Wide wave crests (3.2.2)	25
Curved packets (3.2.1)	9
Small white clouds (3.2.3)	9
Regularly spaced dark features (3.2.7)	6
Emanating from vortex (3.2.5)	4
Extremely long curved features (3.2.2)	2
Lee waves (3.2.4)	1

*Note*. These include features not illustrated in the figures associated with the main article but included in the supporting information. The total number of waves or wave‐like features is 157. The category of waves with long packets and short crests dominates the total. Quantitative properties of these waves are shown in the table of Section SI2 of the supporting information. They are also shown graphically in Figures 16 and 17 and Figures SI3‐1 through SI3‐3 of the supporting information.

### 3.2 Types of Wave‐Like Features

Our survey of JunoCam images has revealed a surprising variety of features with wave‐like morphologies. In order to be inclusive in our inventory, we include here (and in the supporting information) features with any regularly repeated patterns that are three or more in number. The survey below includes many features that have not been discussed previously in the context of atmospheric waves in Jupiter. They are presented in terms of differences in visual morphology, without implication that this differentiation arises from the associated responsible dynamics.

#### 3.2.1 Long Wave Packets With Short, Dark Wavefronts

Long wave packets with short, dark wavefronts represent 79% of the types of waves in our inventory, especially in the Equatorial Zone (EZ) that were also detected in previous studies, particularly from Voyager imaging (Table 1).

##### 3.2.1.1 Orthogonal Wave Crests

**Figure 2:**
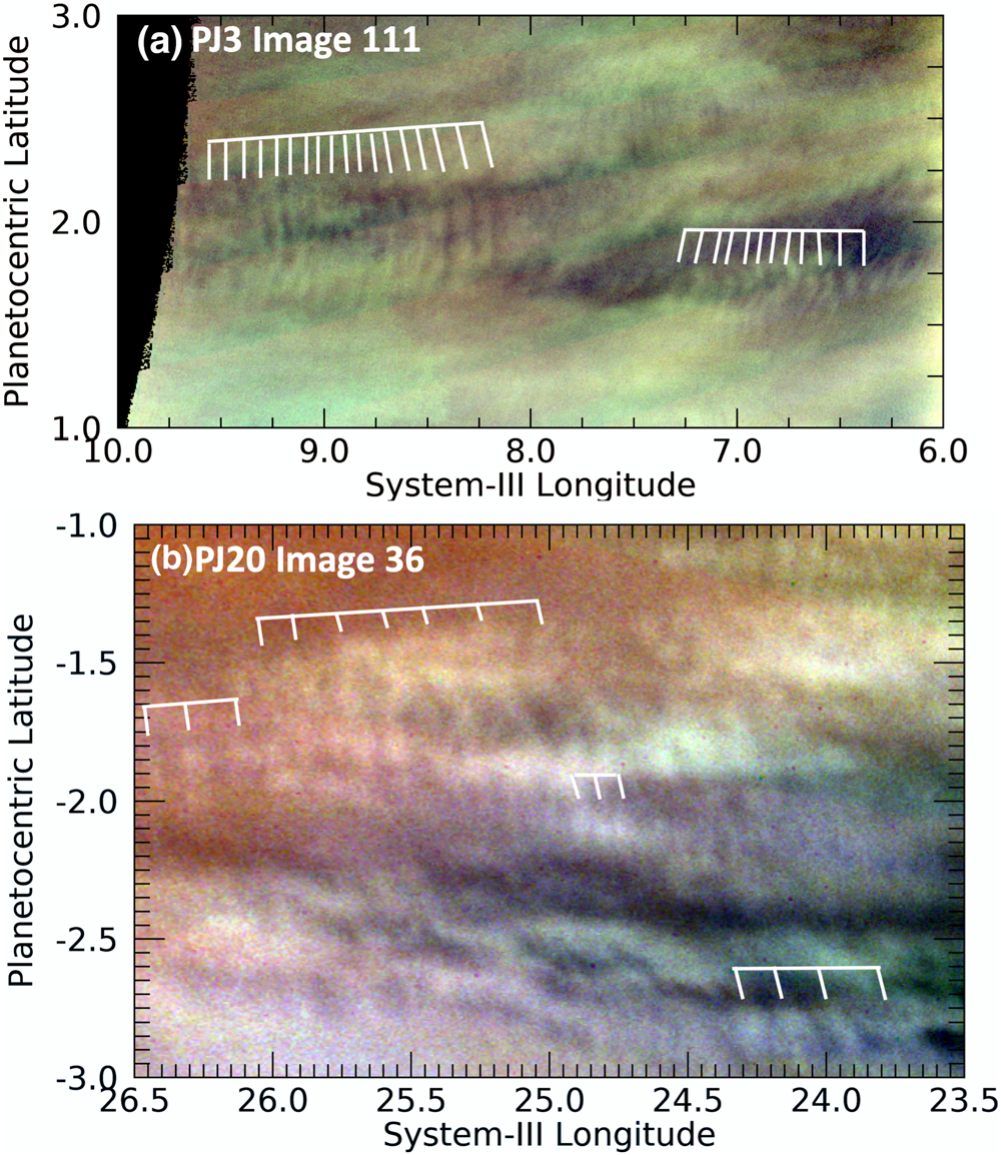
Excerpts from JunoCam color‐composite maps of images in PJ3 illustrating isolated wave packets with shorter wavefronts that are orthogonal to the orientation of the wave packet. In this and all other similar figures in this report, the colors have been stretched extensively in order to make the waves as visible as possible; they have absolutely no relationship with the true colors of the planet. In this and some sequent images here and in the supporting information, broad vertical or diagonal colored bands are artifacts of strong image enhancement to distinguish otherwise faint features.

Figure 2 shows two examples of these waves in which the wavefront is more‐or‐less orthogonal to the direction of the wave packet. The morphology of the waves shown in Figure 2 is most similar to those waves described in the articles cited in Table 1, although they are an order of magnitude smaller. They are most commonly referred to as mesoscale waves, by analogy to their appearance in the Earth's atmosphere. Our search through JunoCam images (see the images in the supporting information) did not appear to sample any of the longer‐wavelength (~1,200–1,900 km) packets detected by previous studies (Table 1), most likely as a result of the limited area over which JunoCam images can cover.

##### 3.2.1.2 “Tilted” Wave Crests

**Figure 3:**
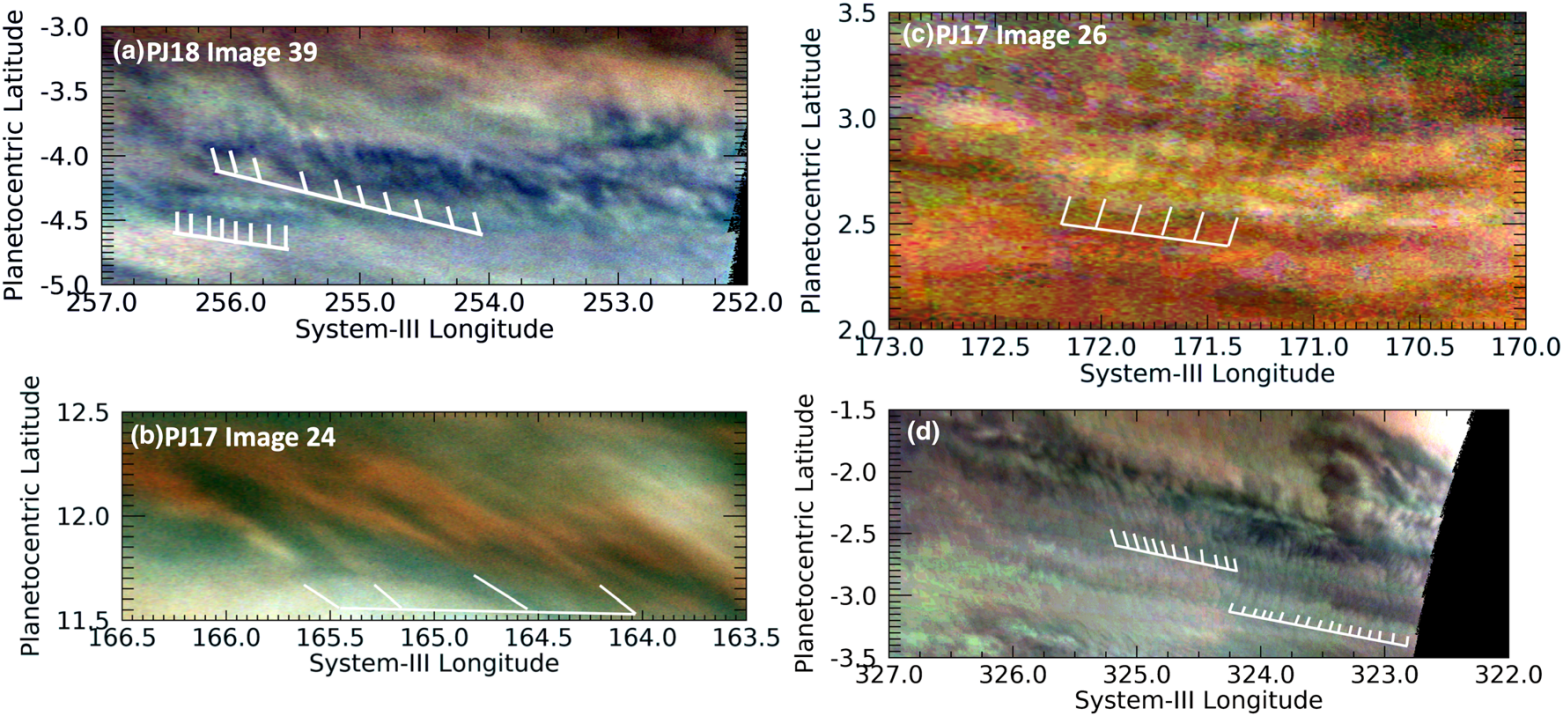
Excerpts from JunoCam maps of images in PJ8 (panel a), PJ17 (panels b and c), and PJ18 (panel d). These illustrate individual wave packets with shorter wavefronts that are not orthogonal (i.e., they are “tilted”) with respect to the orientation of the wave packet direction.

Even more commonly, the detected packets have wavefronts that are not oriented orthogonally to the wave packet direction. Several examples of these “tilted” wavefronts are shown in Figure 3. Simon, Li, and Reuter (2015) stated that this is consistent with an interpretation of the waves as baroclinic instabilities that tilt northward and westward with altitude, as noted on a theoretical basis by Holton (1992) and by observations of waves in the Earth's atmosphere (e.g., Blackmon et al., 1984). However, if the waves represented baroclinic instabilities, their meridional extent depends on the Rossby radius of deformation, which we estimated as L_d_ = NH/f, assuming geostrophic balance. N is the Brunt‐Väisälä frequency, estimated as 0.002 s^−1^ at the tropopause (see Rogers et al., 2016, or Fletcher et al., 2020). H is the atmospheric scale height, approximately 20 km. For a latitude 5° from the equator, the Coriolis parameter, f = 3 × 10^−5^ s^−1^, making L_d_ ~ 13,000 km. This is much larger than the observed meridional extent (~250 km on average) or the wavelengths (distance between wave crests, ~170 km) of these waves, and it increases close to the equator. So this is not likely to be the case, unlike the waves near 14°N discussed by Simon, Li, and Reuter (2015). We argue in a later section that the waves are most likely to be inertia‐gravity (IG) waves. Although wave tilt is better documented for baroclinic instabilities, their existence in gravity waves is not precluded. The generation of tilt is more closely related to the wind‐shear environment, and gravity waves may also tilt with increasing altitude. Plougonven and Zhang (2014) discuss tilts in potential vorticity with altitude for gravity waves studied by several investigators. Detection of tilts implies that we are seeing the upper levels of such waves.

**Figure 4:**
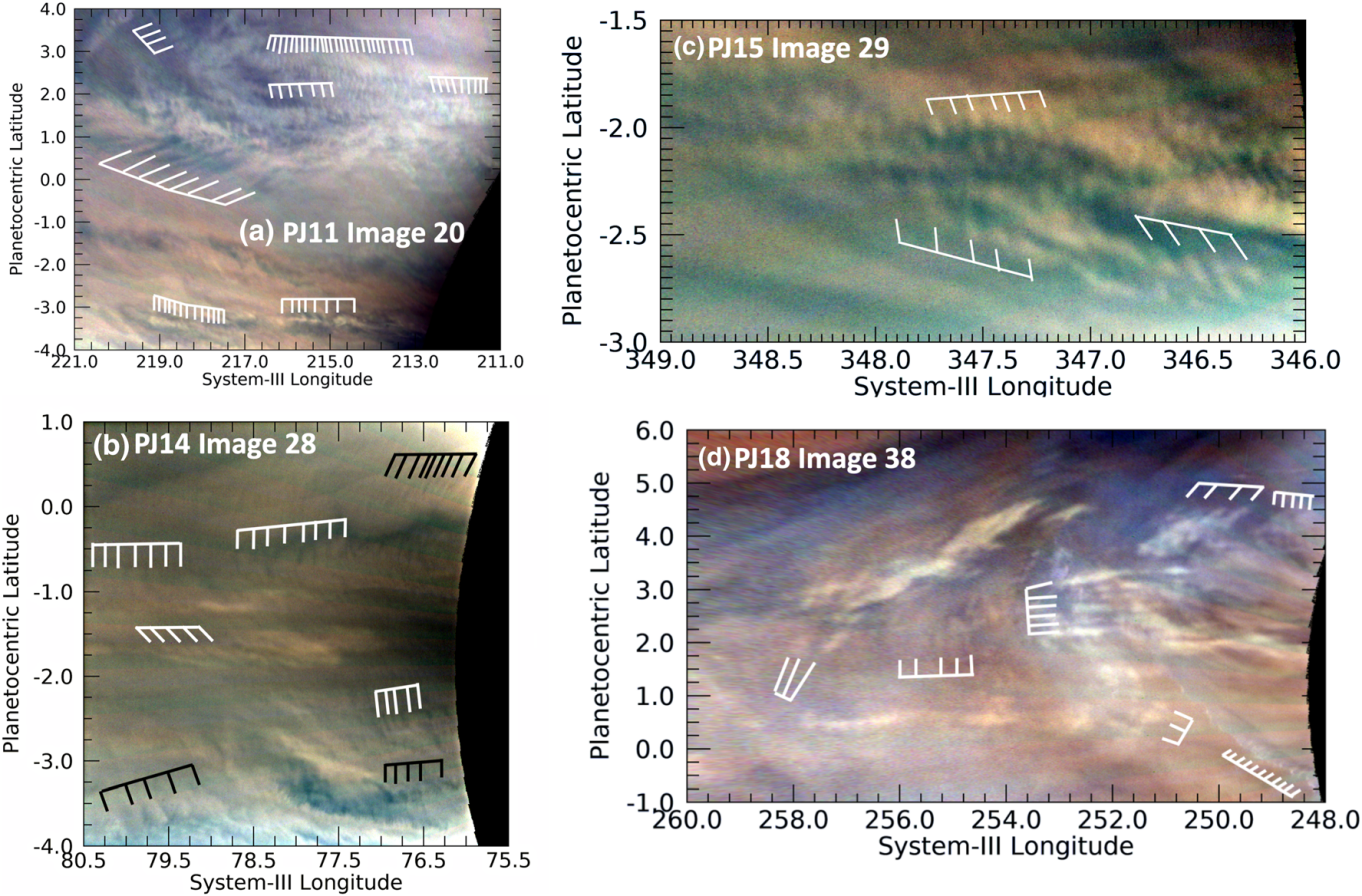
A variety of wave morphologies detected near the equator. As shown in these images, the equatorial region is often populated with both short and long wave trains with both orthogonal and “tilted” wavefronts, often overlapping. Toward the bottom of panels a and d are a set of waves defined by discrete white clouds, more of which are shown closer up in Figure 8.

Several images reveal the presence of large numbers of similar waves, as shown in the various panels of Figure 4. The waves are most often short with wave packets oriented east‐west, although there are many wave packets not ostensibly oriented in any preferred direction (Figure 4d). Some clearly cross one another, implying that the sources of their origin are not uniform. As we just noted, both the meridional extent and wavelength of these waves are much shorter than the Rossby deformation radius, so it is logical to assume that they are formed by and interact with small‐scale turbulence, and thereby propagate the waves in all directions. This is consistent with our observation that few, if any, of these waves are clearly associated with other atmospheric features.

##### 3.2.1.3 Curved Wave Packets

**Figure 5:**
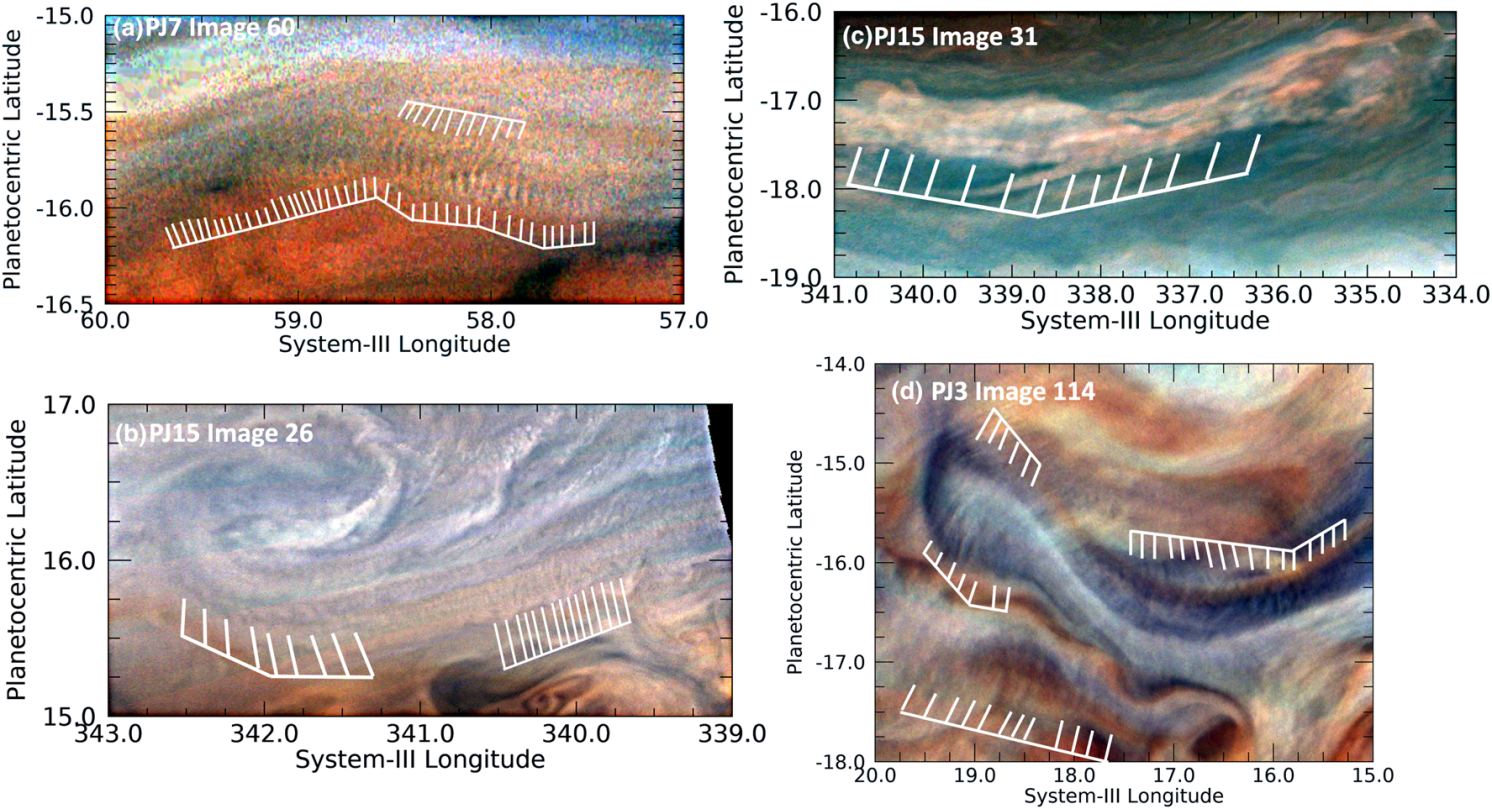
Examples of short wavefronts associated with a curved wave packet. (a) Wavefronts associated with the curvature of the northern boundary of the GRS. A version of this panel appears in Figure 8 of Sánchez‐Lavega et al. (2018). (b) Wavefronts along the southern edge of an anticyclonic eddy in the NEB. (c) Wavefronts on the south edge of a cyclonic circulation in the SEB. (d) Wavefronts that cross a relatively dark lobate feature in the South Equatorial Belt (SEB), a part of a turbulent region west of the Great Red Spot.

Sometimes the short wavefronts are aligned in wave packets that themselves appear to be curved, are associated with larger features, and are not located in the EZ. Figure 5 shows two examples. Figure 5a shows the short wave‐packets associated with the curved northern boundary of the Great Red Spot (GRS) near 15.8°S, described by Sánchez‐Lavega et al. (2018). This is the first of two cases in which multiple images of waves were made, the result of intensive targeting of the GRS by Juno at PJ7. Sánchez‐Lavega et al. (2018) estimated a phase speed for the wave of 45 ± 20 m/s relative to the very rapid local flow and determined that they were consistent with internal gravity waves, given estimates for the Richardson number for that part of the atmosphere that were based on the vertical wind shear deduced from temperature maps of the region (Fletcher et al., 2010). Two other examples of such wave packets imaged at PJ15 are shown. Figure 5b shows one on the south edge of a bright anticyclonic eddy in the NEB near 15.8°N, and Figure 5c shows one on the south edge of a dark cyclonic circulation in the SEB near 17.3°S. Just as for the wave trains in the northern edge of the GRS (Figure 5a), these two wave packets are located on or near the peaks of retrograde (westward) flows that are probably accelerated in these locations because of the circulation.

**Figure 6:**
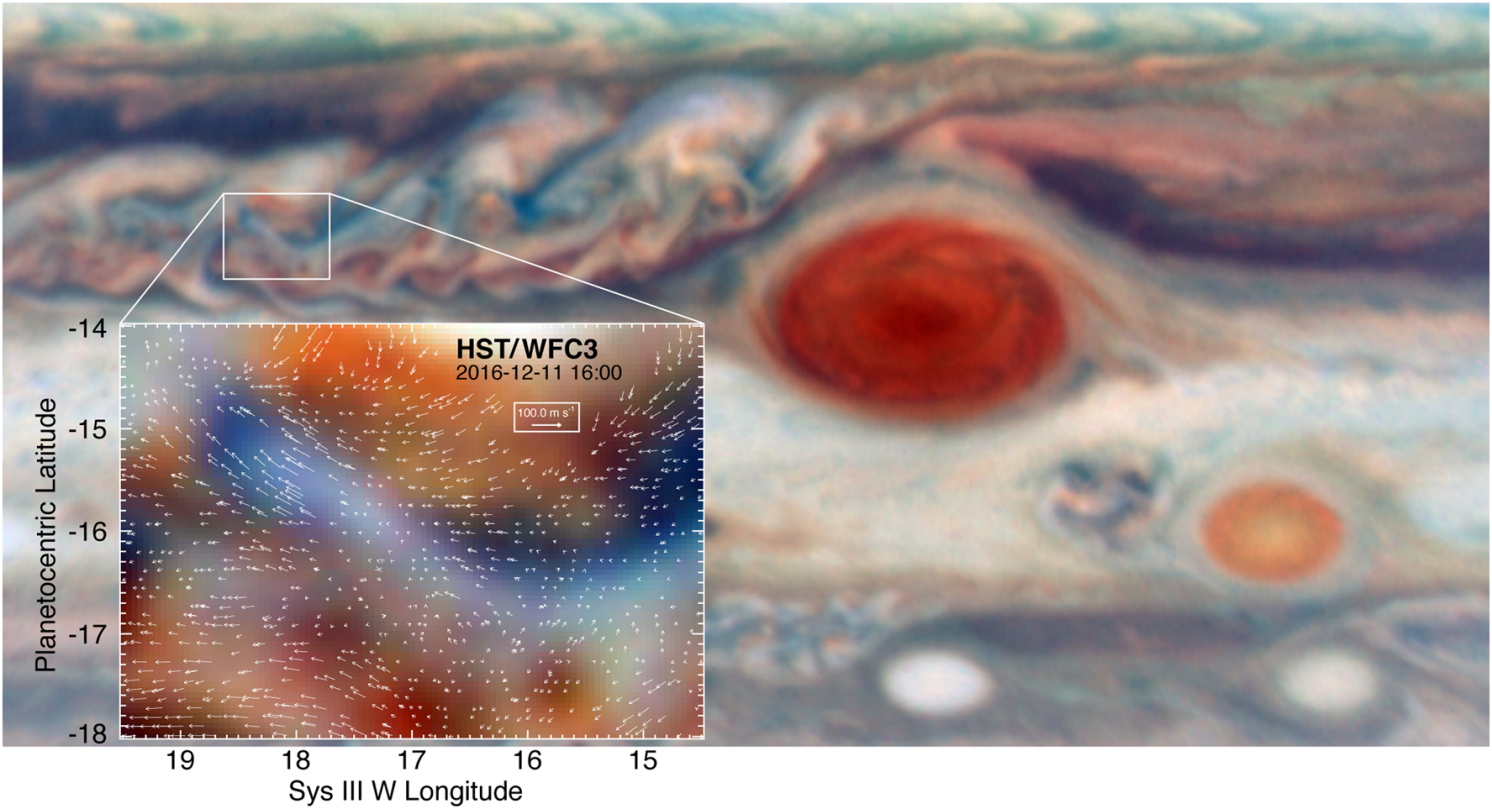
Contextual HST WFC3 image for Figure 5d, showing its position with respect to the GRS. The inset shows the wind field derived from tracking cloud features over a 45‐min interval, as a residual after subtracting the mean zonal wind profile. The winds appear to be a maximum at the western (left) end of the blue lobate feature, with a marked drop in velocities, that is, a region of wind shear, at the edges of the feature. This image was taken within a few minutes of the time at which the JunoCam image in Figure 5d was observed.

Another curved wavefront example is shown in Figure 5d: a dark, lobate feature with short wave crests that are most clearly detectable along its periphery. This feature is located in the chaotic region to the west of the GRS (see Figure 6 for context). Interestingly, the entire chaotic region covers a much larger area to the northwest and west of the GRS, but these waves only appear in the region shown in Figure 5d. The dark part of this lobate feature appears only slightly brighter in 5‐μm radiance than its surroundings using contemporaneous NASA Infrared Telescope Facility (IRTF) observations. Thus, it is likely to be a region of very moderate dry downwelling that only partially clears out particles in cloud layers. Although the series of wave crests appears to line the sharply curved periphery of the dark feature, the crests are more likely to be roughly parallel streaks in a haze that overlies the entire region, with their visibility over the darker regions of this image strongly subdued. This interpretation is reinforced by studies of the winds from Juno‐supporting observations by HST (Wong et al., 2020). Figure 6 shows the results of tracking winds in this region. Relative to the mean zonal winds, the residual winds shown in this figure appear to be flowing up toward the northwest along the dark lobe with speeds of 65 ± 17 m/s. Thus, the waves appearing in Figure 5d are aligned with the local retrograde flow in high‐shear regions. In this respect, they are similar to the curved wave packets described in the preceding paragraph.

#### 3.2.2 Short Wave Packets With Wide Wavefronts

**Figure 7:**
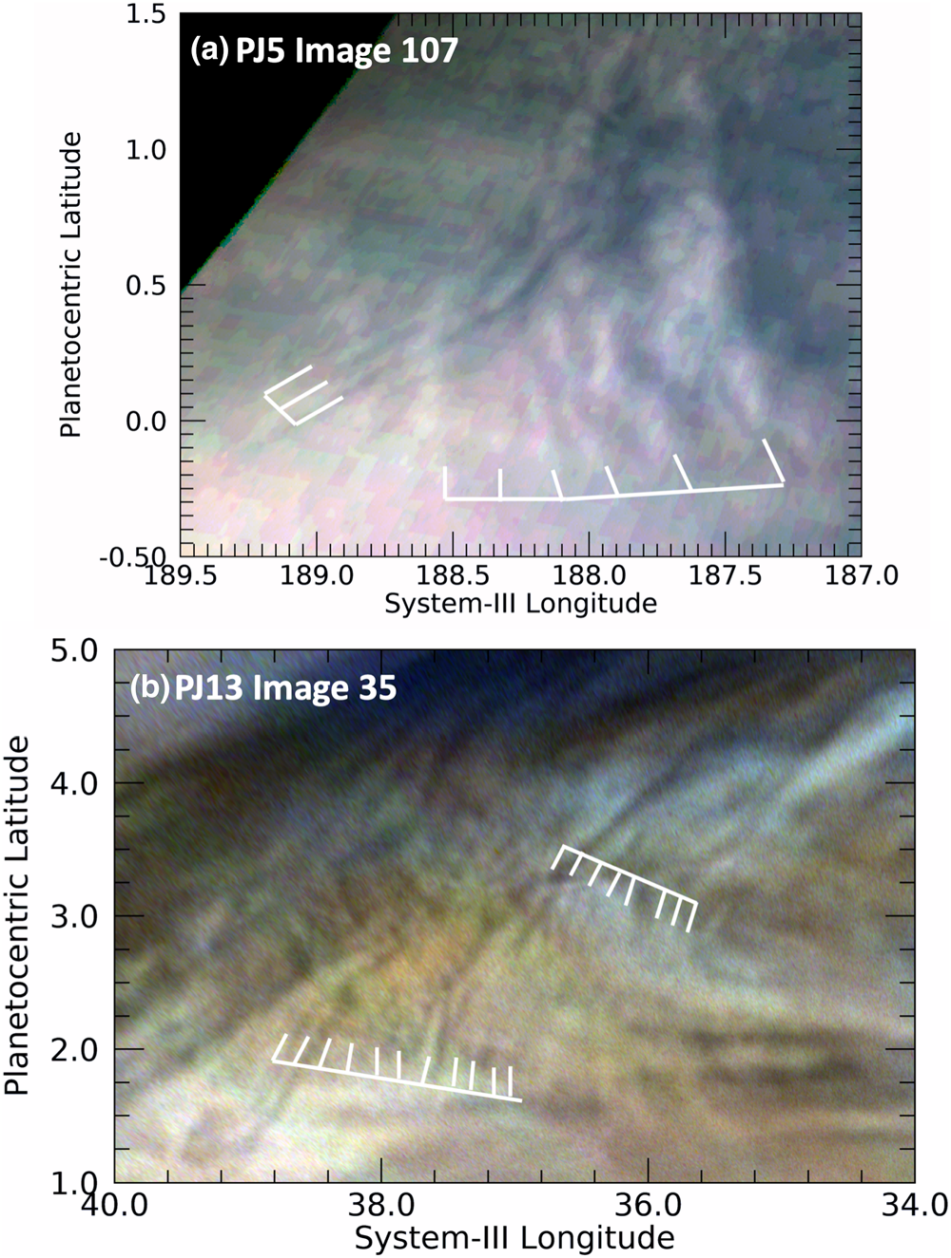
Excerpts from maps showing waves whose wavefronts are larger than the wave packet length. Both are curved and located in the Equatorial Zone. (a) A pair of wave packets, overlapping each other, the westernmost of which contains at least three wavefronts that are much longer than the packet. The overlapping easternmost wave packet has wavefronts and a length that are roughly equal in size. (b) A series of curved waves that appear to extend past the boundaries of the full image.

**Figure 8:**
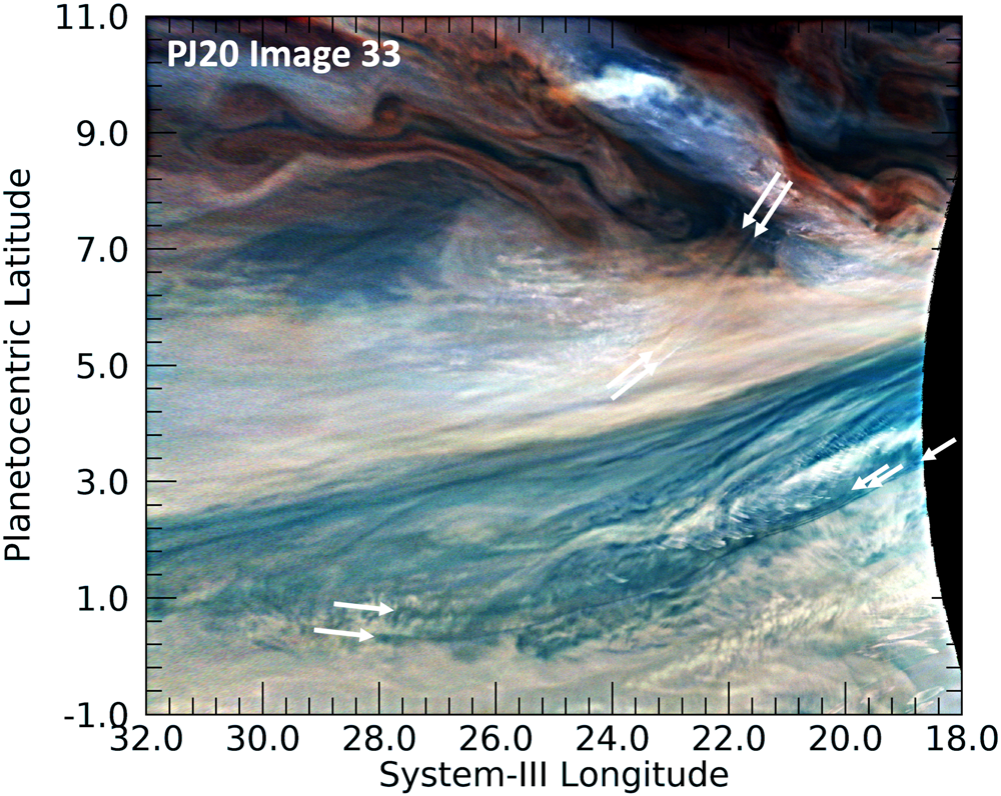
Extremely long, curved features detected in PJ20. Two unusually long features that may be waves were detected near the southwestern extension (“festoon”) of a 5‐μm hot spot, seen here as the dark area. North of the dark area of the festoon is a pair of lines, whose beginnings and ends are marked by white arrows. Among the many waves found south of the festoon, is a set of three closely spaced parallel lines, noted by the arrows that do not begin or end at the same position. Only for two of them is the western end evident. We note that some mild unsharp masking has been applied to this image in order to resolve for the reader the three dark curves at their eastern ends. (The locations of many more of the waves present in this figure are indicated in Figure PJ20_34 in the supporting information.)

Short wave packets with wide wavefronts, shown in Figures 7 and 8, are also detected in our survey. In the Earth's atmosphere, such waves are often associated with thunderstorms producing a brief impulse period with radiating waves. Other curved features situated adjacent to each other are shown in the supporting information, which are shorter and difficult to distinguish from different albedo clouds that are stretched along streamlines (see Figures PJ05_108, PJ14_25a, PJ14_25b, and PJ14_25c). Somewhat similar features were detected in a Voyager image of “spiral” waves to the west of a dark brown cyclonic feature commonly called a “barge” (Simon et al., 2018). Although there is some overlap between these waves and those described in section 3.2.1 in a spectrum of the length‐to‐width ratio of waves, these waves appear to occupy a generally distinct locus in plot of the length versus width of waves (see Figure SI3‐2 in the supporting information).

Other waves are even more distinct. The arrows in Figure 8 show extremely long, closely spaced parallel lines that could be waves. Just as for the wave packets illustrated in Figure 7, both are curved. The pair of lines indicated in the upper part of the figure appear to have no visual association with any nearby feature, although they are situated between the bright (possibly upwelling) spot to the north and the darker region to its south. This darker region is an extension (sometimes called a “festoon”) of a blue‐gray region along the southern boundary of the North Equatorial Belt associated with bright 5‐μm radiances, called a “5‐μm hot spot.” The narrow dark lines indicated in the bottom of Figure 8 are close to the southern boundary of the dark festoon. Although they could simply be long streaks associated with streamlines of flow along the festoon, they appear to be particularly narrow and well defined with sharp edges, particularly at their eastern extents. This differentiates them particularly from far less distinct streaks along the northern boundary of the festoon. They are also accompanied by shorter crests that are aligned perpendicular to the length of the lines. These orthogonal waves are not explicitly indicated in Figure 8 by white grids in order to make the extent of the long lines clearer, but they are illustrated in the same region shown in the supporting information as Figure 20_34a. Orton, Hansen, et al. (2017) detected linear features in the north polar region, but they were associated with the edge of a well‐defined haze region whose boundary could be traced using the 890‐nm “methane” JunoCam filter. JunoCam did not take images of the features indicated in Figure 8 with the 890‐nm filter, and they are below the spatial‐resolution limits of Earth‐based imaging in similar filters. The closest morphological analogies in the Earth's atmosphere might be roll clouds, formerly known as cumulus cloud streets, for example, Yang and Geerts (2006), which are most often detached from but associated with a cumulonimbus base. These are now classified as volutus clouds (https://cloudatlas.wmo.int/clouds-species-volutus.html) by the International Cloud Atlas. Another possibility is that they represent a version of transverse cirrus clouds, identified in upper‐level tropospheric structures in the Earth's atmosphere (Knox et al., 2010).

#### 3.2.3 Wave Packets With Bright Features

Wave packets with bright features appear different from the waves indicated up to this point (Figures 2, 3, 4, 5, 6, 7), which are recognizable by their dark or alternating dark‐to‐light crests. JunoCam has imaged many waves and wave‐like features that are manifested as regular, repeated patterns of bright clouds, visually similar to terrestrial water‐based clouds. We presume that differences between darker and brighter wave crests could be the composition of the material affected. Possibly the waves themselves induce condensation of bright white clouds along their crests, similar to what was seen in the mid‐NEB on much larger scales by Fletcher et al. (2018). This might imply differences in altitude, for example, perturbations of an upper‐tropospheric haze layer near 200–300 mbar (e.g., Braude et al., 2020; Sromovsky et al., 2017) versus those of a condensate cloud, such as a layer of “cirrus” NH_3_ ice particles near the 600‐mbar condensation level. This corresponds to an altitude difference near the equator of roughly 15–20 km, an interval on the order of or less than an atmospheric scale height.

**Figure 9:**
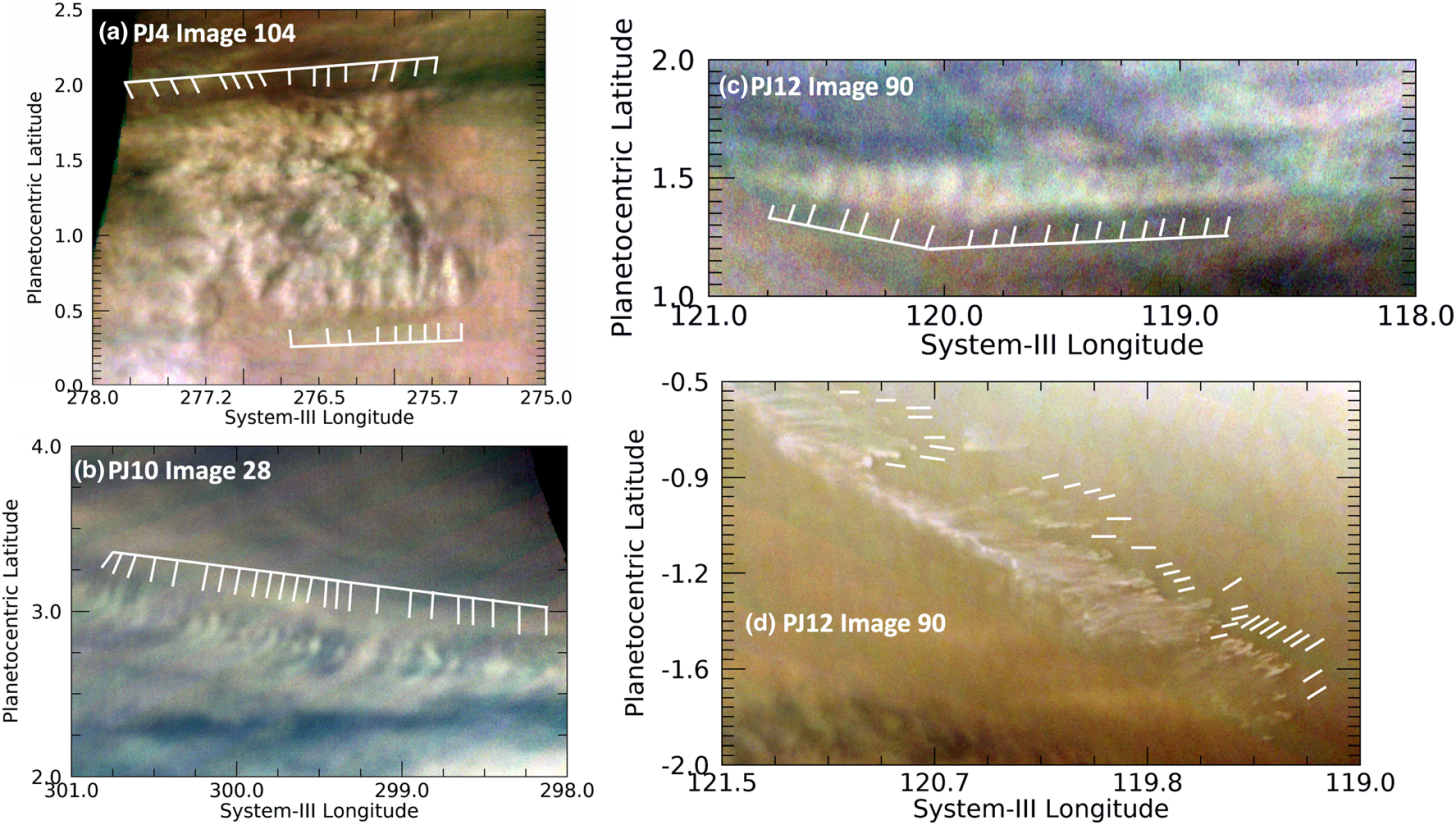
Examples of wave packets defined by bright clouds, all in the Equatorial Zone. (a) A wave packet whose length is roughly equal to its width. Its constituent clouds are clearly higher than their surroundings, given the strong topographic clues from consistent shadowing on their eastern sides. (b) A line of regular clouds with longer, curved southwestern extensions. (c) A similar wave packet of white clouds with narrow wavefronts and a slight curvature. (d) A very curious type of irregular wave‐like feature, extracted from the northeastern portion of Figure PJ12_90a in the supporting information. The feature is reminiscent of ocean foam, with side‐by‐side elongated features that are not uniformly directed and may be higher than the surrounding cloud deck, with a consistent darkening on their eastern sides that might be shadowing.

**Figure 10:**
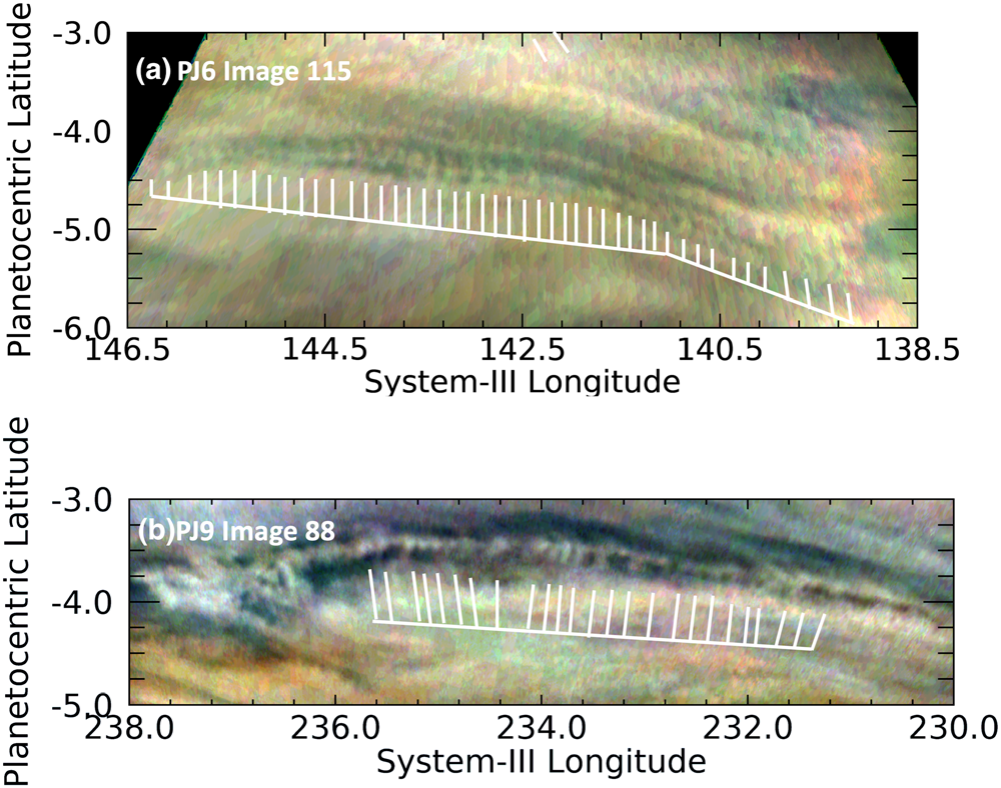
Two detections of regular patterns of relatively bright clouds apparently associated with a fainter central bright region. Both instances involve curved lines. The wave packet in panel a is associated with a similar but fainter pattern to its north, whereas the wave packet in panel b has no such association. Both are in the southern component of the Equatorial Zone.

Figure 9 shows a variety of examples of regular spacings between light‐colored clouds detected by JunoCam. We lack the means to determine whether dark regions adjacent to lighter ones simply represent lower‐albedo regions that are relatively cloudless or actual shadows of the brighter clouds. One likely exception to this are the clouds associated with the wave packet in the upper‐left area of Figure 9a, which appear similar to terrestrial cirrocumulus clouds that have shadows associated with them. (If all of the dark area to the right of the largest dark region is a shadow, then the height of the largest cloud relative to the region around the cloud is on the order of 10 km.) We repeat the caveat of Simon et al. (2015) that such dark features may not be shadows but local regions of aerosol clearing “as atmosphere parcels rise and ices condense out to make the wave crests.” The clouds in the other panels are often arranged in a straight line or a segmented straight line with cirrus‐like wisps trailing away from them. Figure 10 shows other regular patterns of bright clouds that are associated with narrower white features. The narrow meridional extent of these clouds (~150 km or less) is potentially the result of a very meridionally constrained flow. We note that both are curved and could be associated with constraining wind flows.

**Figure 11:**
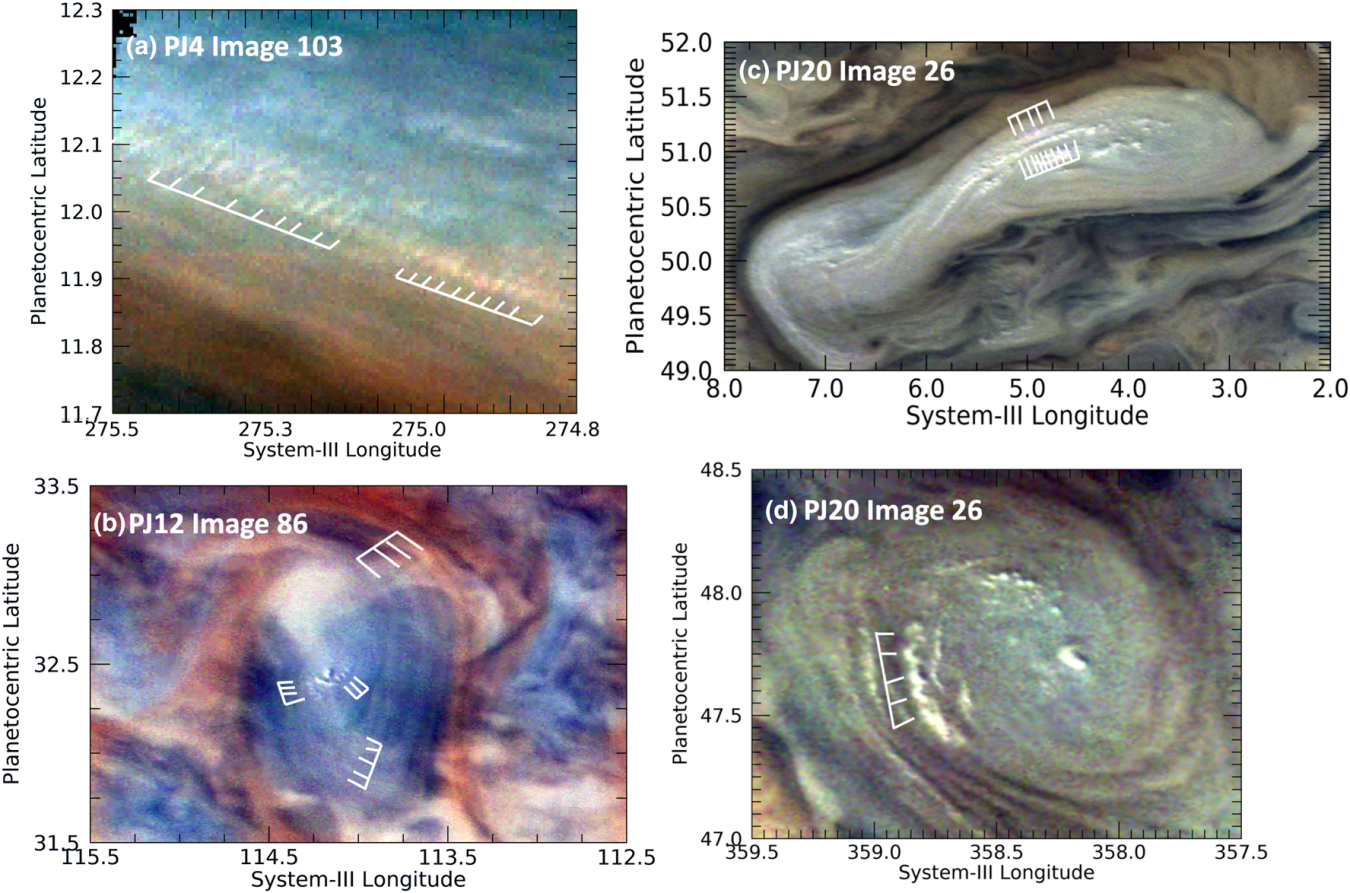
Distinct, very small‐scale white clouds with regular spacing. The spacing and the distinct arrangement along a discrete path suggest a wave‐like structure. (a) An expanded portion of Figure PJ04_103b in the supporting information. (b) A bright cloud, accompanied by short linear arrays of similar bright clouds in an anticyclonic feature in the North North Temperate Belt (NNTB). (c, d) Features from the same image in an area just south of the prograde jet at 53°N. From their morphologies, they are both most likely to be anticyclonic vortices.

Figure 11 shows four instances of very bright, discrete clouds forming regular, extended patterns. These clouds extend to higher altitudes than their surroundings, as evidenced by shadows that often accompany them. Individual clouds such as these appear in various locations elsewhere in the planet, and we will describe and analyze them as a class in a separate report. We include this subset of them in our description of a distinct type of wave. Figure 11a shows a close up of such clouds, an expanded portion of Figure PJ04_103b in the supporting information. A wave packet can be seen that appears to be controlling small, bright cloud features. These are located in a bright patch that is part of a complex system of upwelling disturbances in the North Equatorial Belt (NEB), also known as “rifts.” Figure 11b shows a weak anticyclonic feature, in the center of which is a central bright cloud, accompanied to its southeast through southwest by short linear arrays of similar bright clouds. Two are shown with white grids that indicate individual cloud features that are resolved. Figures 11c and 11d also show individual clouds that comprise a wave packet, similar to the linear packet shown in Figure 11a. In Figure 11c, the clouds appear like balls or small smears, whereas in Figure 11d they appear like C‐shaped arcs. If the dark regions accompanying the clouds in Figures 11b, 11c, and 11d are shadows, it would imply that they are clouds whose tops are higher than the surrounding darker cloud deck. Based on the incident angle of illumination, we estimate from the length of its shadow that the central cloud in Figure 11b is only 3–4 km above the surrounding cloud deck. A similar estimate for the range of shadow lengths associated with various bright clouds in Figure 11c implies that they are 5–12 km above the surrounding cloud deck. From the shadows associated with several C‐shaped arcs in Figure 11d, we estimate that they rise as much as 6–13 km above the background cloud deck. There are other similar features in both Figures 11c and 11d, but they are not fully resolved. Although we cannot determine with absolute certainty that these clouds extend down to the level of the surrounding cloud deck, that is the impression one gets if the accompanying dark regions are interpreted as shadows. If these bright clouds do extend vertically downward to the surrounding cloud deck, then they appear less like linear versions of stratiform clouds on the Earth, than a series of upwelling cumulus clouds in which the intervening spaces between them simply represent regions of compensating subsidence.

#### 3.2.4 Lee Waves

**Figure 12:**
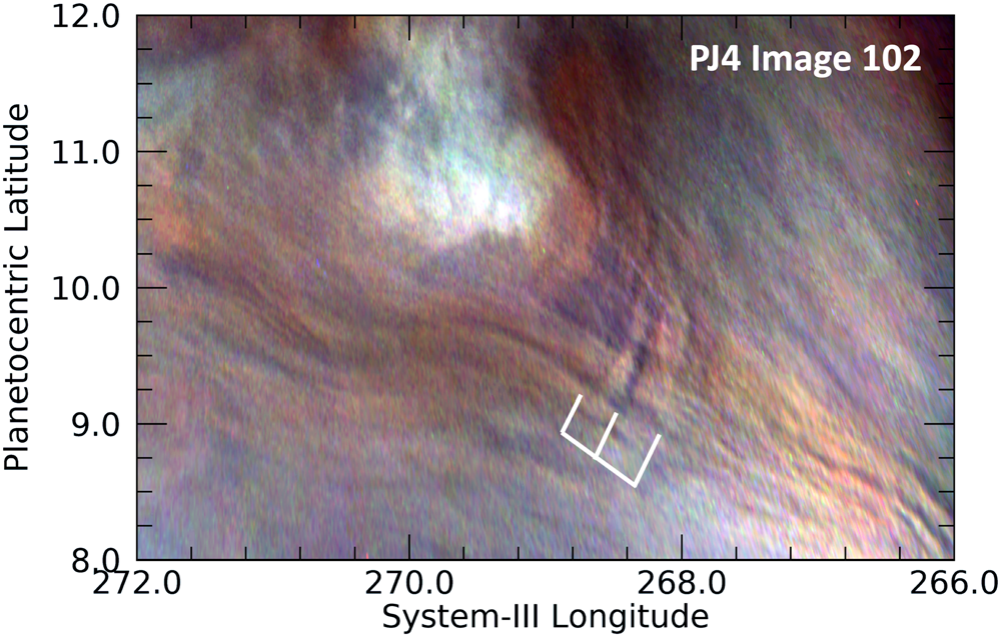
The single unambiguous detection of lee waves in the JunoCam images. We can presume that the long streaks appearing diagonally in this figure are tracking the streamlines of local winds. The lee waves indicated by the white grid lines are orthogonal to the elongated streaks and are likely to be downwind of the bright convective plume in the upper part of this image.

Lee waves are stationary waves generated by the vertical deflection of winds over an obstacle, such as a mountain, a thermal updraft or a vertical vortex. Unlike the Earth, there are no mountains in Jupiter's atmosphere, but there may indeed be the dynamical equivalent. If the long streaks in Figure 12 that stretch diagonally (upper left to the lower right) in the figure are tracking streamlines associated with local winds, and the winds are moving from the northwest to the southeast (upper left to the lower right in the figure), then the lee wave is the three‐wavefront feature indicated by the white grid lines that is orthogonal to the flow. This requires that the local winds are passing not only around the bright upwelling anticyclonic vortex in the upper left of the frame, but also over it, consistent with very subtle streaks seen over the bright vortex. We note that not only the three waves indicated but also the lines that appear to be tracing the wind flow are elevated above the background cloud field, as marked by the shadows on their eastern sides. The most prominent of the shadows is on the eastern side of the central wave, the length of which implies that the peak of the wave is some 10 km about the background cloud deck. This is, in fact, the only example of such a wave in our survey. One reason could be that other atmospheric features are too high to permit flow over them, compared with the relatively young anticyclonic vortex in Figure 12.

#### 3.2.5 Waves Associated With Large Vortices

**Figure 13:**
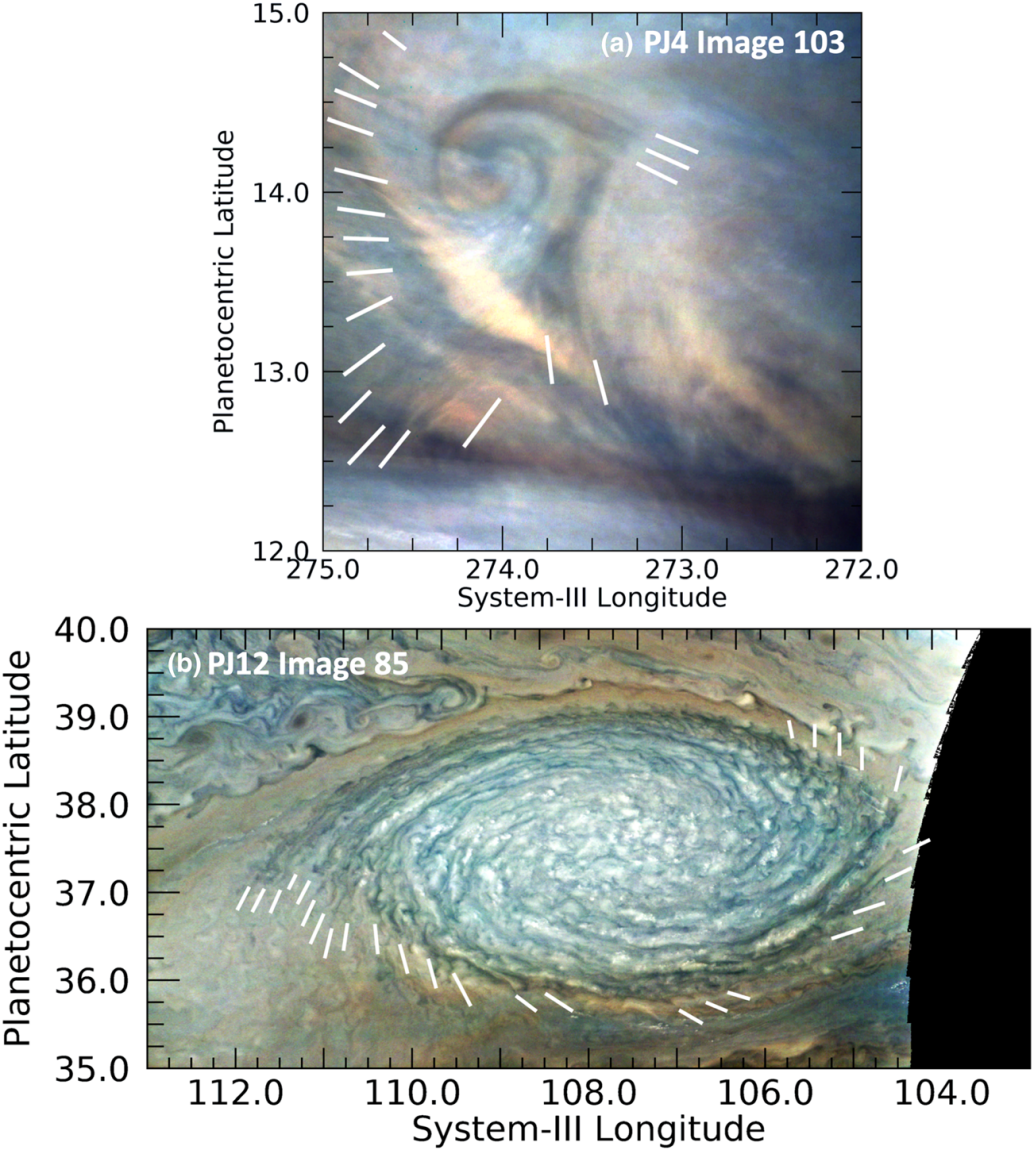
Wave‐like features detected near vortices. (a) A very compact cyclonic feature with a set of extended radial wavefronts in the North Equatorial Belt (NEB). (b) A regular set of dark lines emerging from the ends of the internal spiral structures in an anticyclonic white oval.

Waves associated with large vortices are shown in Figure 13. Figure 13a shows a very compact cyclonic feature with a set of extended radial wavefronts in the North Equatorial Belt. These resemble similar structures in terrestrial cyclonic hurricanes. The waves delineated in Figure 13a show morphological similarities to “transverse cirrus bands” (hereafter “TCB”) identified in upper‐level tropospheric structures on Earth (Knox et al., 2010). TCB are defined by the American Meteorology Society as “Irregularly spaced bandlike cirrus clouds that form nearly perpendicular to a jet stream axis. They are usually visible in the strongest portions of the subtropical jet and can also be seen in tropical cyclone outflow regions.” (American Meteorological Society, 1999). TCBs are also frequently observed in midlatitude mesoscale convective systems (MCS) and in extra‐tropical cyclones. Numerical studies (Kim et al., 2014; Trier et al., 2010) have successfully replicated these cloud features and therefore have provided insight to their formation. Currently, there is no consensus regarding the dynamics responsible for TCB in all their observed forms (Knox et al., 2010). Multiple interacting factors have been implicated in the genesis of these features, including gravity waves, Kelvin‐Helmholtz instabilities, weak or negative moist static stabilities, and vertical wind shears (Dixon et al., 2000; Knox et al., 2010; Trier et al., 2010).

There are some common characteristics that TCB share in the Earth's atmosphere. First, the bands frequently originate in a region of anticyclonic vorticity, positive divergence, and in weak or negative static stability (Trier et al., 2010). Second, the majority of the bands appear in regions of strong relative vorticity gradient and often persist beyond the life of the originating MCS (Lenz et al., 2009). Third, the bands are often oriented along the vertical wind gradient, which provides surprising evidence they share some dynamical characteristics with boundary‐layer horizontal convective roll vortices (Kim et al., 2014, Trier et al., 2010), commonly observed on Earth as cloud streets (Yang & Geerts, 2006). Fourth, there is evidence that gravity waves propagating below the cirrus cloud deck, the release of latent heat within the bands, and longwave cooling above and longwave warming below the bands appear to favor the formation of TCB. In addition to Figure 13a, the wave‐like features shown in Figures 2a, 3d, 4a, 5, 9a, and 9c appear similar to terrestrial TBC. Although it is difficult to know if they are true analogs in the absence of detailed horizontal wind measurements of these clouds (as well as temperature measurements to understand the 3‐D wind gradients), their morphologies are suggestive. If this is the case, then complex small‐scale dynamics may be operating in and below the Jovian ammonia cloud deck not dissimilar to those on Earth.

The wave features in Figure 13a bear some resemblance to similar features found in tropical cyclones. Animations of tropical cyclones show high‐frequency circular gravity waves in the central dense overcast cirrus shield (“CDO,” Molinari et al., 2014) emanating from vigorous convection in or near the eyewall. Perhaps more relevant to the appearance of the features in Figure 13a, radial‐aligned TCB are also commonly observed as “spokes,” which are more or less oriented orthogonally to the gravity waves. In many cases, the circulation of the parent vortex twists the spokes to appear like the teeth of a circular saw blade or as long thin curved filaments. In addition, shallow‐water numerical modeling of vortex dynamics using the Explicit Planetary Isentropic Coordinate (EPIC; Dowling et al., 1998) in Brueshaber et al. (2019) also displays curved wave‐like features similar to those in Figure 13a, but their waves are certainly due to gravity waves formed during the merger of like‐signed vortices for which we have no direct evidence in this figure.

On the other hand, for the much larger anticyclonic white oval in Figure 13b, it is possible that the curved cloud features appearing there to be a manifestation of gravity waves. The spatial resolution of this image is sufficient to see both the internal spiral structure of the white oval and a regular set of dark bands extending to its exterior. Anticyclones on Jupiter, such this one, often feature a high‐speed “collar” surrounding a calmer interior (e.g., Marcus, 1993). The shear of the high‐speed wind against slower winds outside of the vortex may be sufficient to generate a Kelvin‐Helmholtz wave, which may explain the scalloped appearance of the white clouds adjacent to the surrounding red clouds.

#### 3.2.6 Long, Parallel Dark Streaks

**Figure 14:**
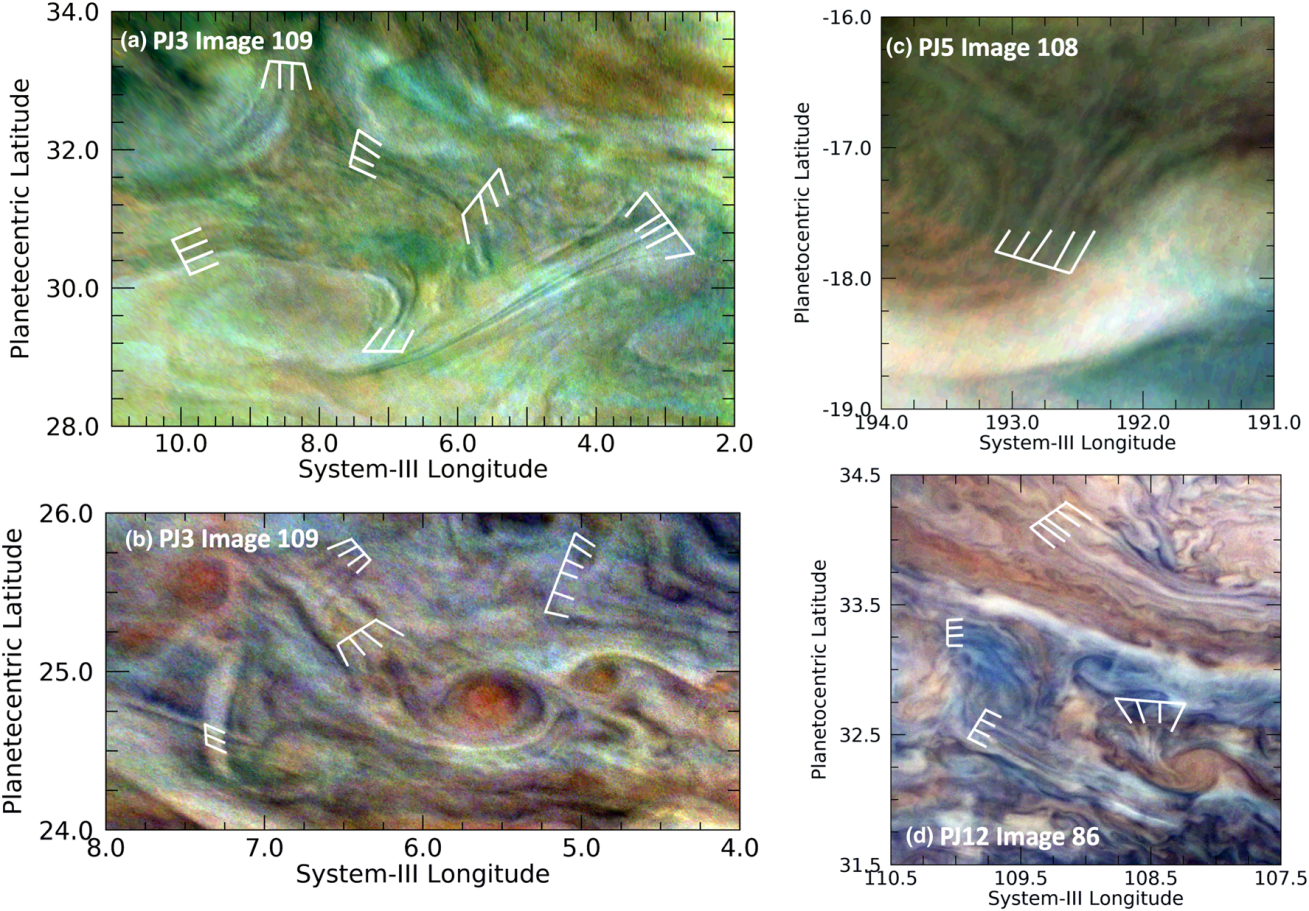
Examples of very long and nearly evenly spaced parallel streaks. (a) Several of these detected in a limited region of the North Temperate Zone (NTZ). (b) A region just south of the area in panel a on the same perijove in the northern component of the North Temperate Belt (NTBn). (c) Parallel streaks located in a pale strip of the southern component of the South Equatorial Belt (SEBs). (d) Multiple examples of parallel cloud streaks in the North North Temperate Belt (NNTB).

Long, parallel dark streaks are detectable at midlatitudes. Long streaks are seen in many areas of Jupiter's cloud system, usually with a non‐uniform and chaotic pattern (e.g., the diagonal ones in Figure 12). But, as shown in Figure 14, some are seen in very regularly spaced parallel bands. In several cases, the parallel banding is not only regularly spaced but sinusoidal in behavior, with a distance between crests ranging between 280 and 360 km. All such features are detected far from the equator. Their orientation suggests that they are tracing out the direction of flow on streamlines, in often complicated patterns, with lengths from 500 to 3,800 km (an upper limit that may be constrained by JunoCam's field of view). Almost all of the parallel streaks in the examples shown in Figure 14 are associated with larger atmospheric features, although those features do not appear to be located where the streaks originate. In Figure 14a, one set of these appears to be “flowing” around an anticyclonic vortex in the lower left. It and a set of streaks in the center of the feature have topography, with shadows apparent on their eastern sides. In Figure 14b, long streaks possibly are associated with streamlines “flowing” around small, red anticyclonic vortices. The North Temperate Belt (NTB) was very turbulent at the time of these observations, following a great disturbance in the preceding months (see Sánchez‐Lavega et al., 2017). A semi‐transparent triplet of short, dark bands in the top left of this figure can be seen lying across longer bands that appear to be tracing wind flow. Figure 14c shows parallel streaks located between a weak cyclonic eddy on the left and a bright wave‐like streak aligned with the SEBs retrograde jet, at the bottom edge of the panel. Figure 14d shows several parallel cloud streaks in this turbulent part of the North North Temperate Belt (NNTB). Some are associated with the small cyclonic vortex in the lower right side of the panel. Often, the streaks appear to be on top of other features, implying that they represent flow that is manifested in a haze layer overlying deeper cloud layers. The best analog to these features lies not in the Earth's atmosphere but in Saturn's. Ingersoll et al. (2018) examine high‐resolution images of Saturn's clouds taken during the Cassini mission's “proximal orbits.” Their Figure 3 shows a flow around a vortex that is very similar to one around the vortex in Figure 11a. For their similar “thread‐like filamentary clouds,” they suggest that the implied laminar flow implies extremely low values of diffusivity and dissipation, which further quantitative analysis of these observations may verify is the case for these scales in Jupiter, as well.

#### 3.2.7 Unusual Features

**Figure 15:**
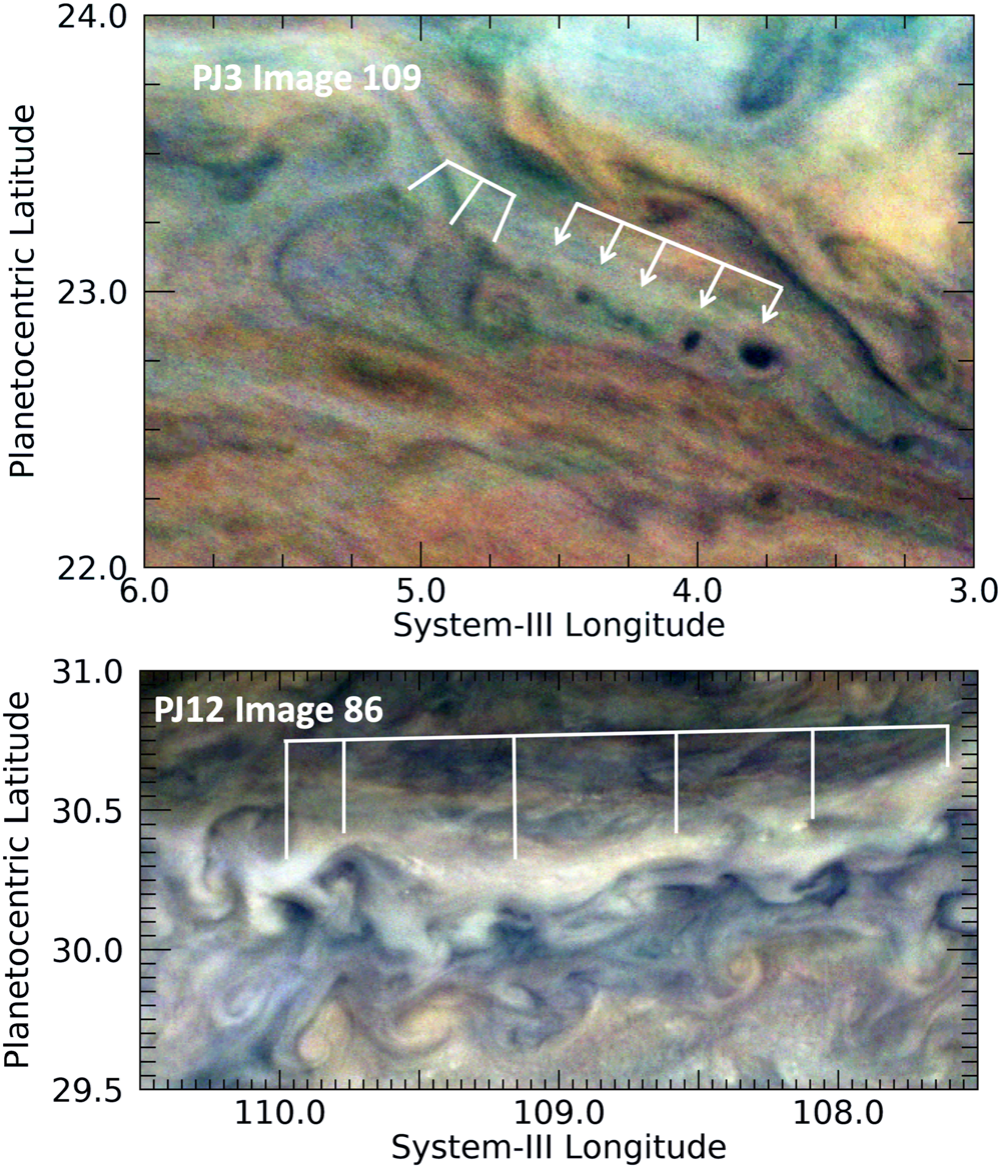
Unusual quasi‐wave‐like features in the northern hemisphere. (a) Three curved wavefronts next to relatively dark circular features indicated by the arrows. (b) A series of repeated patterns in the North Temperate Zone.

Unusual features are shown in Figure 15, which we might classify as waves only in the most general sense. Figure 15a shows a series of features with a regular spacing: three curved wavefronts next to an unusual series of relatively dark ovals indicated by the arrows. The dark ovals may be connected dynamically to the wavefronts, because they continue in the same direction and have roughly the same wavelength. The morphology of the three wavefronts implies that flow is from the northwest. We do not see an array of short, dark, curved lines elsewhere, so their spatial association with each other is extremely unusual. They are located near the boundary between the turbulent northern component and the smooth, orange southern component of the North Temperate Belt. Figure 15b shows a limited series of repeated patterns along the southern edge of an unusual white band located at the turbulent boundary between the northern and southern components of the North Temperate Zone. This short sequence bears some resemblance to a Karman vortex street, although one that may be dissipating or disrupted.

### 3.3 Quantitative Measurements of Wave Properties

#### 3.3.1 Measurements of Meridional Distribution and Size Properties

Measurements were made of physical properties of all of the waves and wave‐like features discussed. A table of all of these is available in the supporting information. Features are identified by Perijove and File number. Measured quantities are the number of waves, the mean System‐III longitude, mean planetocentric latitude, length and width of the wave train, the mean wavelength (distance between crests), and the tilt of the wave with respect to the orientation of the wave packet.

**Figure 16:**
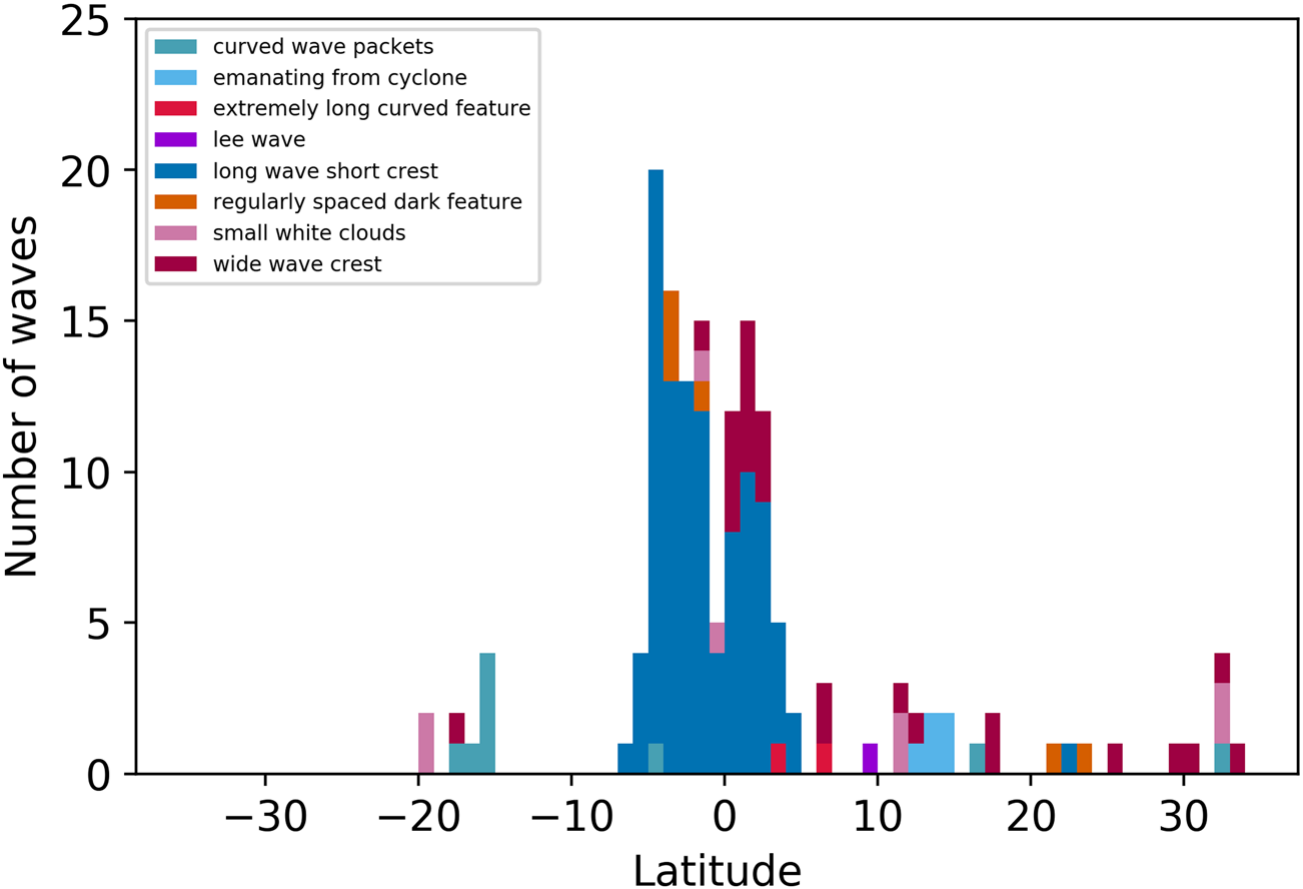
Histogram of waves and wave‐like features detected in PJ1‐20 by JunoCam. Different types of waves and wave‐like features are denoted by different colors and identified by the key. Each corresponds to a different wave morphology as discussed in section 3.2. The bin size is 1° in latitude.

**Figure 17:**
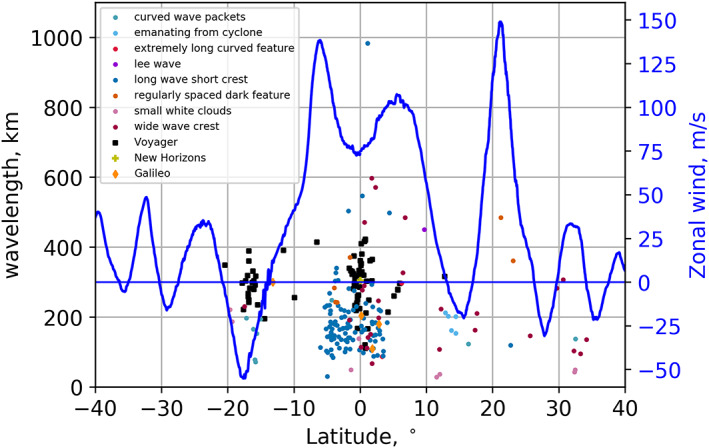
Wavelengths of waves and wave‐like features detected in PJ1 and PJ3‐PJ20 by JunoCam. Measurements of different types of wave morphologies are color‐coded as in Figure 15. Mean zonal wind velocities for 2017–2018 (Wong et al., 2020) are plotted in blue. Values for Voyager, New Horizons, and Galileo are taken from their respective references in Table 1. Wavelengths for wave packets detected by HST and ground‐based images (Fletcher et al., 2018; Simon et al., 2018) are greater than 1,100 km and clustered around 14.5°N (see Table 1).

Figure 16 shows a histogram of the occurrence of waves as a function of latitude. In order for the reader to distinguish between different classes of wave‐like features, some of which are arguably not propagating waves, we have separated out the different types of waves by morphology as discussed in the preceding sections. Table 2 shows our count of the different categories of waves. The overwhelming majority of wave‐like features are clustered between 7°S and 6°N latitude, the relatively bright EZ. These features are dominated by long wave packets with short wave crests, the type of waves detected by Hunt and Muller (1979) and discussed by Simon, Li, and Reuter (2015) as mesoscale waves observed at low latitudes by previous imaging experiments. These waves fall within the relatively bright EZ and appear to be subclustered with fewer waves between 1°S and the equator than between either 7°S and 1°S or the equator and 6°N. The next most populous category is waves with short packet lengths and long crests, which appear to be distinct not only because they appear to be clustered differently in length versus width ratios but also because they mostly populate latitudes between 1°N and 3°N. Waves that are generally associated with or influenced by larger features, most often associated with curved wave packets, are the next most abundant feature. These include the curved wave packets at the northern boundary of the GRS (Figure 5a), the wave packets on the southern edge of a cyclonic circulation in the SEB (Figure 5b) and on the southern edge of an anticyclonic eddy (Figure 5c), wave packets associated with the lobate feature in the chaotic region west of the GRS (Figure 5d), and parallel stripes near a weak eddy (Figure 14c). All are located in regions of retrograde flow, as shown in Figure 17. All other types of features are detected less frequently (Table 2) and are scattered in the northern hemisphere. No waves of any type were detected south of 7°S other than the ones between 17°S and 20°S that are associated with larger features. There may be a small selection effect associated with the observations, since latitudes in the northern hemisphere are observed with an average spatial resolution that is higher than in the southern hemisphere, arising from the fact that the Juno spacecraft perijove is in the northern hemisphere and moving northward by about a degree of latitude for each successive, highly elliptical orbit. Perijove latitudes ranged from 3.8°N for PJ1 to 20.3°N for PJ20. Arguing against this is the fact that waves were detected in the southern hemisphere with wavelengths between 70 and 200 km, meaning that waves of this size range would have been detectable elsewhere if they were present. Such waves might, in fact, be present but undetectable if the hazes making them visible in the northern hemisphere were not present in the southern hemisphere outside the EZ, for some reason.

Is the observed distribution of waves associated with other indicators of upwelling or turbulence? Clearly the preponderance of waves in the EZ is not correlated with the frequency of lightning detections, as no detections of lightning have been associated with that region, either historically (e.g., Borucki & Magalhães, 1992; Dyudina et al., 2004; Little et al., 1999) or in the broad survey by the Juno Microwave Radiometer (Brown et al., 2018) that is sensitive to lightning discharges in the EZ (Juno's Waves instrument, Imai et al., 2018, could not detect lightning in the EZ because the field lines do not reach Juno's orbit.). The presence of water ice is one indirect measure of upwelling, and its detection from Voyager IRIS data by Simon‐Miller et al. (2000) revealed a distribution that included the EZ but was significantly higher at latitudes south of ~10°S. This is consistent with our results only in the limited sense that several waves were associated with the GRS and its surroundings. Another indirect measure is the presence of pristine ammonia ice, as measured most recently by New Horizons (Reuter et al., 2007), which determined that spectrally identifiable ammonia clouds (SIACs) occurred “near active storms or upwelling regions,” which includes some regions in the EZ and is more broadly consistent with several of our specific observations at higher latitudes. New Horizons did not detect SAICs near the GRS, as the typically chaotic region to its northwest was not active during the New Horizons encounter. From the Juno mission itself, the striking deep column of concentrated ammonia at 2°N to 5°N detected by the Microwave Radiometer (MWR) instrument implies upwelling (Bolton et al., 2017; Li et al., 2017), which is consistent with the concentration of waves there. This is consistent with contemporaneous ground‐based observations (de Pater et al., 2019; Fletcher et al., 2016, 2020). However, we detected an equal number of waves in the southern component of the EZ, where there was not nearly as great a concentration of ammonia gas, so this particular correlation is imperfect. We note that from studies of cloud properties from reflected sunlight, the full EZ is known as a region in which tropospheric clouds and hazes extend higher than other locations on the planet outside the GRS, as evidenced by the general concentration of upper‐atmospheric opacity historically (e.g., West et al., 1986) and in more recent work (see Figures 4 and 12 of Sromovsky et al., 2017, and Figure 13B of Braude et al., 2020) or by the distribution of disequilibrium constituents (see Figure 4 of Orton et al., 2017). This is consistent with the entire EZ being a region of general upwelling.

Figure 17 plots the distribution of mean wavelengths for different types of waves and wave‐like features as a function of latitude, co‐plotted with mean zonal wind velocity. The minimum distance between crests is 29.1 km for the spacing between the discrete white features shown in Figure 10a. Not significantly larger is the 30.9 km between crests of waves in a low‐latitude long wave packet with short crests. These values are available in a table in the supporting information. The variability of wavelengths within a single packet is typically no greater than 20–30%. The equatorial waves with long packets and short crests in the EZ have wavelengths that are clustered between 30 and 320 km, with most between 80 and 230 km in size. The bimodal appearance of the distribution of EZ waves is not consistent with the distribution of waves detected from Voyager (Simon, Li, & Reuter, 2015), which also has several wave packets distributed at latitudes south of the EZ (see Figure 17). Similar to our study, most of these are associated with the GRS. Similar to Voyager, all the waves detected in JunoCam images in regions of retrograde flow are associated with discrete atmospheric features, such as the GRS. The virtual absence of waves observed in Voyager images covering the northern hemisphere is ostensibly the opposite of what we observe with JunoCam, although the key in Figure 16 shows that many of the wave‐like features in the northern hemisphere might not have been categorized as waves by Voyager investigators.

#### 3.3.2 Measurements of Wave Phase Speed

The most diagnostic criterion between different types of waves is the propagation speed. The waves in the EZ were discovered by Voyager 1 and described by Hunt and Muller (1979), who found them to have low or zero speeds relative to their surroundings (whether in a plume tail or equatorial clouds). Simon, Wong, and Orton (2015) also found little relative motion for these waves in Voyager 2 and Galileo Orbiter images. Arregi et al. (2009), studying Galileo Orbiter images, likewise found no measurable relative motion for waves on the equator, but a phase velocity of 35 (+/−8) m/s for waves at 3°S. Simon, Wong, and Orton (2015) adopted the conclusions of Flasar and Gierasch (1986), Bosak and Ingersoll (2002), and Arregi et al. (2009) that these waves detected by Voyager and Galileo images were best classified as inertia‐gravity (IG) waves, a conclusion we do not revisit here. On the other hand, Simon, Wong, and Orton (2015) differentiated the waves detected by New Horizons as Kelvin waves from those by Galileo and Voyager as IG waves on the basis of their phase velocity, crest length, and location; they measured a non‐zero velocity (80 ± 5 km/s) relative to the local zonal wind for the Kelvin waves that are confined to the equator compared with the IG waves, which are near stationary (upper limits to the phase velocity of 40 m/s or less). Unfortunately, the Juno spacecraft and orbit configuration that provides such close‐up observations of Jupiter's clouds strongly limits our ability to determine velocities, and regions are rarely observed at adequate spatial resolution more than once per perijove. Subsequent perijoves typically observe longitudes that are far from the preceding one. Observations of the Great Red Spot in PJ7 are one exception (Sánchez‐Lavega et al., 2018), as noted above. Another is the circulation associated with a large cyclonic feature observed by both JunoCam and ground‐based facilities (Iñurrigarro et al., 2020).

We made another attempt in PJ20 to observe one region several times during a perijove, focusing on the northern component of the EZ, Images 33 through 37 (formally JNCE_2019043_20C00033_V01 through JNCE_2019043_20C00037_V01). We examined these quite carefully using a recently developed upgrade in our geometric calibration, which used limb‐crossing times to correct for otherwise undetected errors in the data‐acquisition timing. The results showed no change in the location of waves (marked in Figure PJ20_34a in the supporting information near 27.5°W longitude and 0.5°N latitude) over the 6‐min,4‐s interval between the first and last images of this sequence. We quantify this using a very conservative standard of 2 pixels for the pointing uncertainty, equivalent to 14 km for Image 33 and 18 km for Image 37—a linear dependence on the distance of the spacecraft from the waves. Using 16 km as an estimate of the mean displacement, this is equivalent to an upper limit for the phase speed of 44 m/s, a value consistent with a supposition that these are IG waves.

Moreover, based on morphology alone, the New Horizons waves were slightly curved, had a consistent distance between wavefronts of 305 ± 25 km, a wave train that spanned the entire visible equator (more than 200,000 km in packet length), and were centered at the equator, spanning ±2° in latitude (see Figures 1 and 2 of Simon, Wong, & Orton, 2015). The waves that we detected here have a broad range of wavelengths and crest lengths and are located at latitudes significantly far from ±1.5° of the equator, and many wave packets are very short. Therefore, we suggest that these types of waves detected in JunoCam images, more similar to those seen in Voyager and Galileo observations, are most likely to be IG in origin.

## 4 Conclusions and Future Work

Juno's public‐outreach camera, JunoCam, detected a plethora of waves or wave‐like features in its first 20 perijove passes. Of these 157 features, 100 are waves with long, somewhat linear packets and short crests, identified as mesoscale waves in earlier studies. Many of these have wave crests that are nearly orthogonal to the wave packet orientation, although others that were tilted compared with this orientation. Another 25 wave packets were detected with short packets and long crests. As a group, they are likely to be features that are truly propagating waves. They are more in number than was detected by Voyager imaging in 1979, and they include waves that are smaller in wavelength than any detected by previous missions. These waves form the vast majority of features detected in this study, and they are concentrated in a latitude range between 5°S and 7°N. Short wave packets often appear in several different orientations and sometimes overlap one another. Almost none of these appear to be associated with other features except for waves that appear to be oriented in lines of local flow, including packets with crests that appear darker than the local background or with bright features. These bright features appear both as discrete, tall clouds with shadows that imply they are higher than the background darker cloud deck and simply as brighter features that have wispy “tails” and are connected to one another by an equally bright but narrow, elongated cloud. The difference between wide and narrow packets is presumably related to the width of the flow that is responsible for the wave. There were fewer waves in the EZ between the equator and 1°N than there were immediately north and south of this band, which was different from the waves detected by Voyager imaging in 1979 that were more equally distributed.

Other waves, prominently those outside the EZ, are clearly associated with or influenced by other features. These include short‐crested packets following the slightly curved path at the northern extent of the GRS, others associated with an anticyclonic eddy in the NEB and a cyclonic circulation in the SEB, and one associated with the turbulent flow west of the GRS. Three lee waves were detected in the wake of an upwelling anticyclonic vortex that were some 10 km above the surrounding cloud deck. More features were detected that had repeated, wave‐like features but may not represent propagating waves. Some of the linear arrangements of discrete white clouds followed the edges of vortices; although regular in spacing, these features may not represent propagating waves so much as alternating positions of upwelling and subsiding vertical flows. Several features appeared within or emanating from vortices. Two sets of extremely long, curved features were detected near the edges of a southwestern extension of a dark blue‐gray region associated with high 5**‐**μm radiances at the southern edge of the NEB. Long, sinuous parallel streaks were detected, some with nearly sinusoidal lateral variability, that were analogous to features observed by the highest‐resolution imaging of Saturn's atmosphere by Cassini (Ingersoll et al., 2018). No waves were detected south of 7°S that were not associated with larger vortices, such as the GRS. No waves or wave‐like features were detected in regions of retrograde mean zonal flow that were not associated with larger features, similar to the waves detected by Voyager imaging.

We had limited opportunities to classify waves on the basis of phase speed. Sánchez‐Lavega et al. (2018) determined that the waves located at the northern extent of the GRS were internal gravity waves from their propagation speed with respect to the local flow, based on a displacement over a 9‐min interval between initial and final images. (Internal gravity waves are similar to IG waves but where Coriolis forces are not considered to be important.) JunoCam seldom observes features more than once and usually with insufficient time to note a displacement. Our attempt to observe features in the EZ on PJ20 resulted in a 6‐min interval over which no motions were detected for equatorial features, providing an upper limit of wave motions that was not inconsistent with inertia‐gravity waves. However, the waves detected in the EZ were not located directly at the equator, which bounded the Kelvin waves detected by New Horizons imaging (Simon, Wong, & Orton, 2015). Otherwise, the waves detected in the EZ are morphologically similar to those detected by Voyager, which Simon, Wong, and Orton (2015) classified as inertia‐gravity waves. These waves may well be associated generally with the upwelling winds that characterize the EZ.

Work will continue to document and detect waves and wave‐like features in Jupiter's atmosphere, including further attempts to examine regions over longer time intervals, although we note that observations of waves in the EZ will be lower in spatial resolution as the latitude of successive perijoves migrates northward by about 1° per perijove. We will also look for simultaneous measurements of waves in the near infrared by the JIRAM experiment to provide some constraints on the altitude of these features, which were otherwise only loosely constrained by occasional measurements of associated shadows. Furthermore, we expect that we and others will use these observations as a motivation to engage in comparisons with terrestrial analogs and numerical simulations that will further our understanding of the origin of these features and their implications for the dynamics of Jupiter's atmosphere at these small scales and their relation to the larger picture of planetary dynamics at depth.

## Supporting information



Supporting Information S1
