# Supplementary material for: A Survey of Small‐Scale Waves and Wave‐Like Phenomena in Jupiter's Atmosphere Detected by JunoCam
Source: J Geophys Res Planets. 2020 Jun 28;125(7):e2019JE006369. doi: 10.1029/2019JE006369 (PMC7380317; doi:10.1029/2019JE006369)
Supplement: Supplementary file 1 — Supporting Information S1 [file JGRE-125-e2019JE006369-s001.docx]

**Supplemental Information for**

**A Survey of Small-Scale Waves and Wave-Like Phenomena in Jupiter’s Atmosphere Detected by JunoCam**

Glenn S. Orton^1^, Fachreddin Tabataba-Vakili^1^, Gerald Eichstädt^2^, John Rogers^3^,

Candice J. Hansen^4^, Thomas W. Momary^1^, Andrew P. Ingersoll^5^, Shawn Brueshaber ^6^, Michael H. Wong^7^, Amy A. Simon^8^, Leigh N. Fletcher^9^, Michael Ravine^10^, Michael Caplinger^10^, Dakota Smith^11^, Scott J. Bolton^12^, Stephen M. Levin^1^, James A. Sinclair^1^, Chloe Thepenier^13^, Hamish Nicholson^14^, Abigail Anthony^15^

^1^Jet Propulsion Laboratory, California Institute of Technology, Pasadena, California, USA

^2^Independent scholar, Stuttgart, Germany

^3^British Astronomical Association, London, UK

^4^Planetary Science Institute, Tucson, Arizona, USA

^5^California Institute of Technology, Pasadena, California, USA

^6^Western Michigan University, Kalamazoo, Michigan, USA

^7^University of California, Berkeley, California, USA; SETI Institute, Mountain View, California, USA

^8^NASA Goddard Space Flight Center, Greenbelt, Maryland, USA

^9^University of Leicester, Leicester, UK

^10^Malin Space Science Systems, San Diego, California, USA

^11^ National Center for Atmospheric Research, Boulder, Colorado, USA

^12^Southwest Research Institute, San Antonio, Texas, USA

^13^Glendale Community College, Glendale, California, USA*

^14^Harvard College, Cambridge, Massachusetts, USA

^15^Golden West College, Huntington Beach, California, USA**

*currently at the University of California, Davis

**currently at the University of California, Berkeley

SI1. Catalog of all detected waves and wave-like phenomena.

This Supplemental Information file illustrates all of the waves and wave-like phenomena, that were detected in this survey, with visual guides to their locations and properties. As in the main article, they are shown in cylindrically projected format. The maps are adjusted to compensate for illumination variations across the field and rendered in a format of 180 pixels per degree. The scale of the map excerpts shown below has been rendered to the nearest half degree. For ease of identification, the maps are stretched to the second power of the signal. Further stretching of each color has been used in the images shown both in the primary article and those shown below. We took a radiometric point at or near the minimum of the radiance histogram and set it to zero and a radiometric point at or near the maximum of the radiance histogram and set it to 255. *No attempt was made to make color or hue of the map excerpts look “Jupiter-like.”* Some mitigation of this extreme approach was necessary in cases when strong camera artefacts or otherwise strong coloration resulted that would distract the reader from the wave patterns we intended to illustrate. When necessary, other approaches to illustrating the waves were used, such as unsharp masking; those instances are noted in the figure captions.

White lines or arrows are used to indicate most of the waves we detect in each figure. The figure captions refer to these as grids. For some images, the waves are so abundant in the field that not all were identified, because the grids themselves would obscure many of the waves.

Figure numbers are identified using the perijove (PJ) number and a short version of the image number within the perijove, with features shown chronologically – typically following northern to southern latitudes, following the sequence of an image set. Full image file identifiers are also provided, as shown for these images on the Mission Juno web site metadata files (for example, see

<https://www.missionjuno.swri.edu/junocam/processing?source=all&ob_from=&ob_to=&perpage=16>. )

So the first image, from perijove 1, is titled Figure PJ01_6171, whose image file is JNCE_2016240_00C06171_V01.

There are instances in which the same region is observed in more than one image. For economy of space, we illustrate only a single example here.

In the interest of thoroughness, we include several examples of repeated patterns whose identification as waves or even wave-like might be marginal. We note these in the captions and suggest that this image set as a whole might generate discussions beyond the otherwise narrow scope of this article. We identify the context of the region illustrated using traditional nomenclature (see Rogers 1995).

No images were taken during PJ2 due to a spacecraft safing event, and the limited observations by JunoCam that were possible during PJ19 were insufficient to resolve any waves or wave-like features.

Here and in the main article the term ‘PJ’ refers to each orbit’s “perijove”, or closest approach to Jupiter, when is when all JunoCam images of relevance were made.

Additional contextual information about all of the JunoCam images from which these observations were made are available in summary reports and images by co-author J. Rogers for

PJ1-PJ10 at https://britastro.org/node/7982,

PJ11-PJ17 at <https://britastro.org/node/12137>, and

PJ18-onward at https://britastro.org/node/17650.


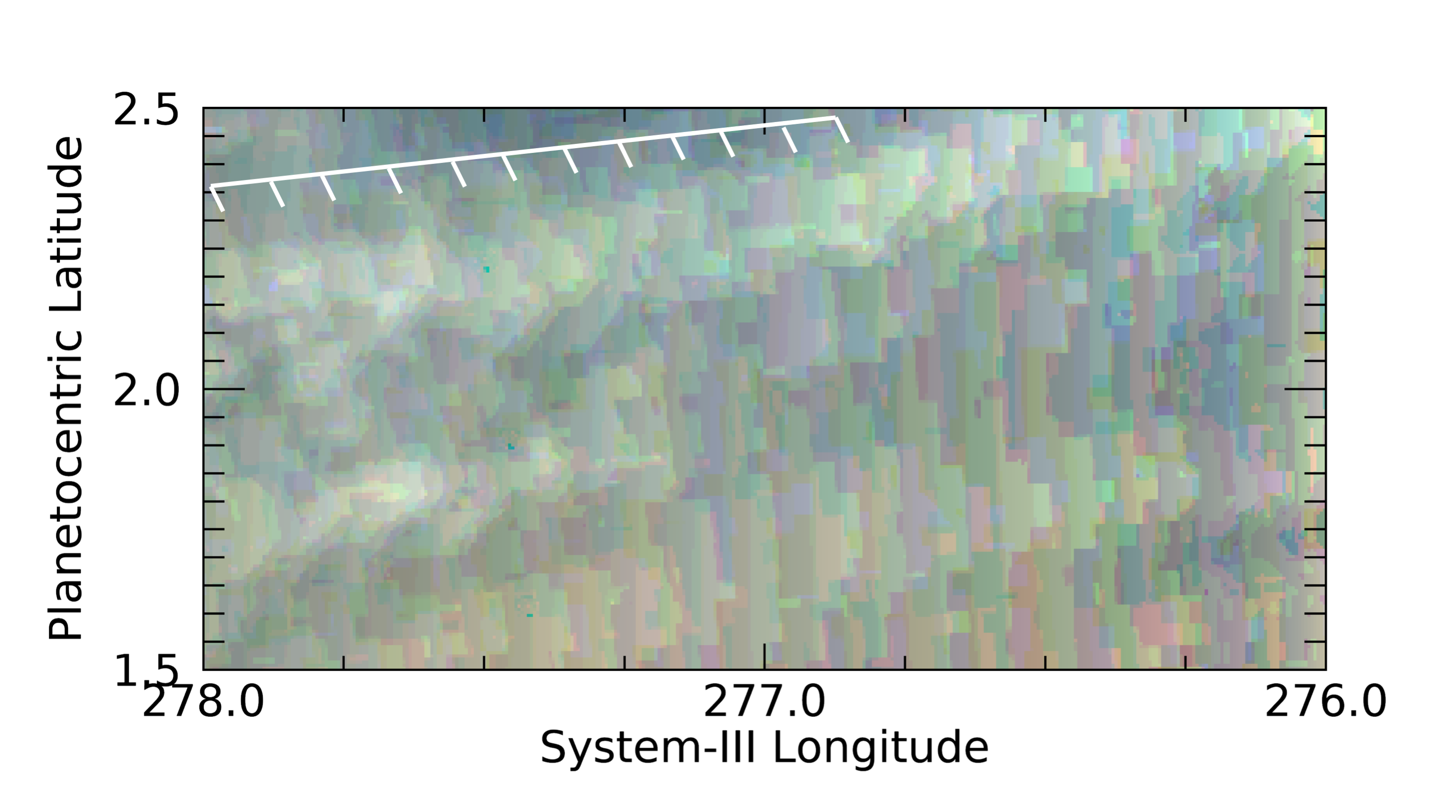


Figure PJ01_6171. JNCE_2016240_00C06171_V01. Note that PJ01 was a test PJ, not a science PJ, and many JunoCam images had image-compression artefacts, including this one. Nonetheless, a regular pattern of waves can be identified in all three colors. A repeatable pattern in the Equatorial Zone (EZ) is indicated by the white grid.


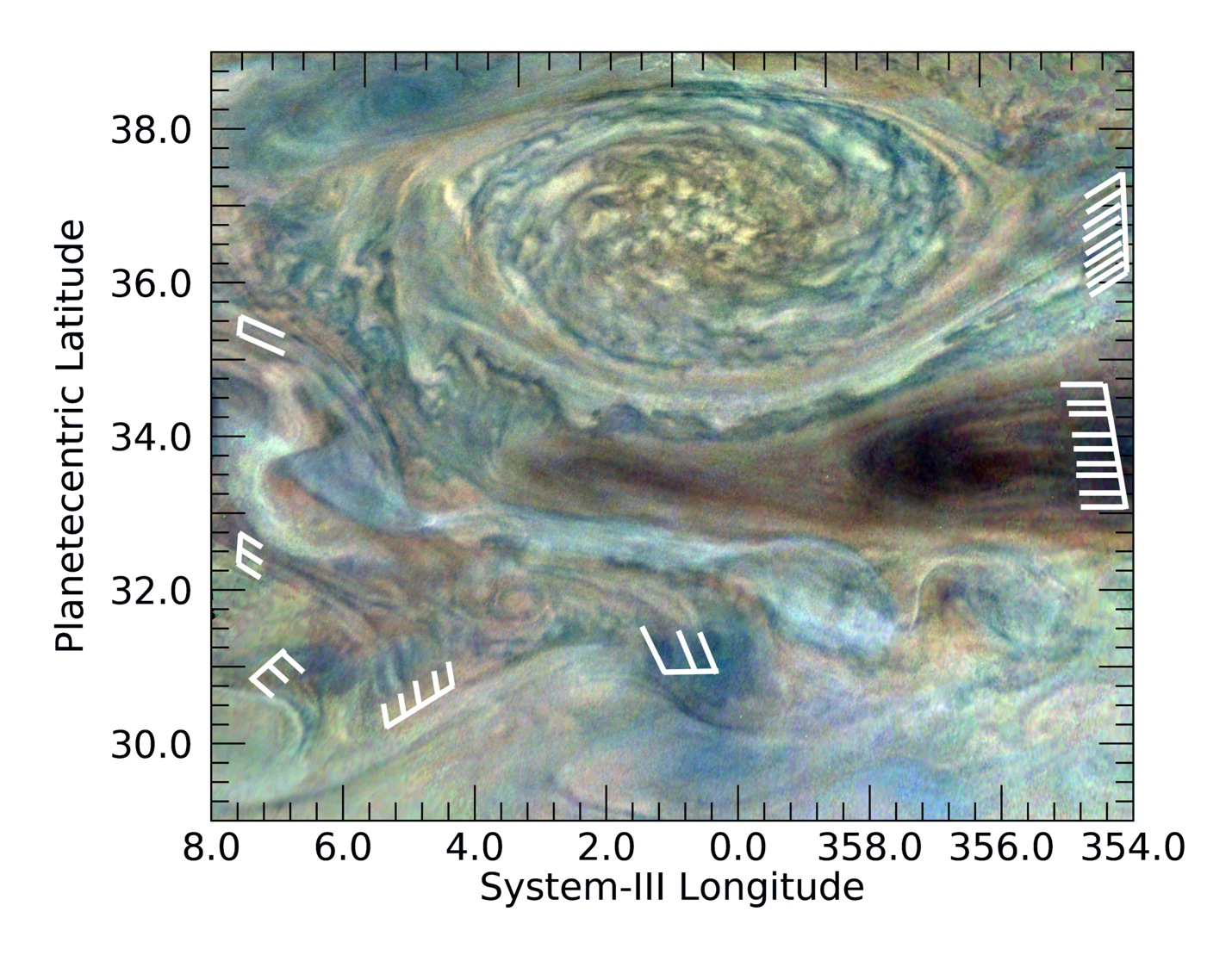


Figure PJ03_107. JNCE_2016346_03C00107_V01. There are many linear and curved streaks in this region of Jupiter that are associated with flow to the south of a reddish anticyclonic vortex, identified in co-author Rogers’ reports as ‘NN-LRS-1’ (for North-North Little Red Spot #1). Some of the curved feature in the mid-to-lower left of this figure rise above the background cloud deck, as shown by the shadows they cast. Wave-like lines extend out from the NN-LRS-1 to its southeast and parallel some in the North North Temperate Belt (NNTB), the dark region to its south. The lighter region to the south of the NNTB is the northern component of the North Temperate Zone (NTZ).


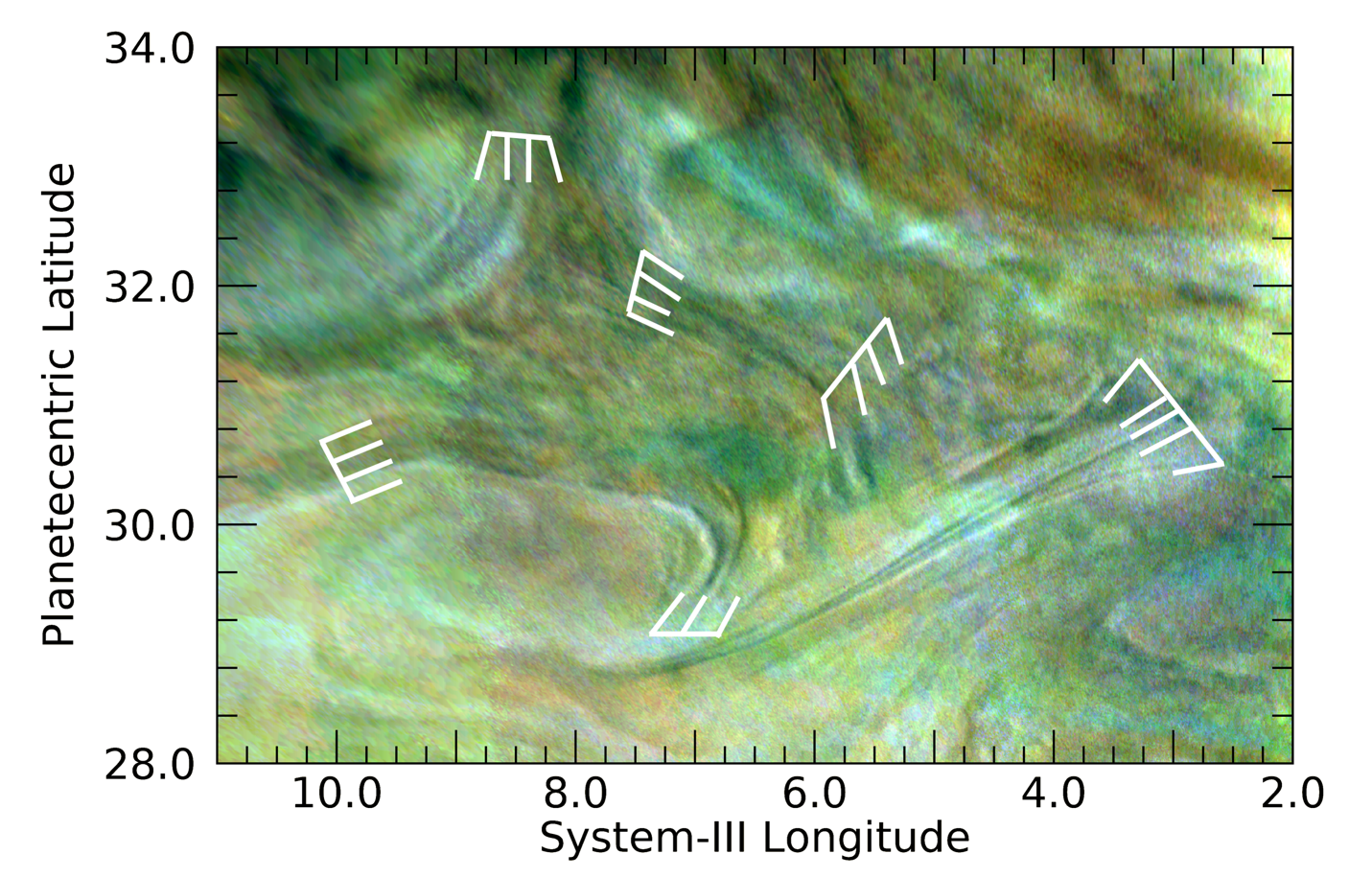


Figure PJ03_109a. JNCE_2016346_03C00109_V01. There are curved and linear cloud banks, identified by their shadows, associated with an anticyclonic vortex (lower left) in the North Temperate Zone. They are part of a circulation around the vortex, which is the central one of three appearing in Image 109; part of the eastern one can also be seen in this figure (lower right). These vortices are associated with the North North Temperate Belt south (NNTBs) jet at 31.7°N and may be moving with it. Other associated lines that probably trace atmospheric flow are also indicated. The color scheme has been modified to minimize artefactual green and red colored bands; in doing so, some whitish-colored haze bands can also be seen that may be associated with the flow.


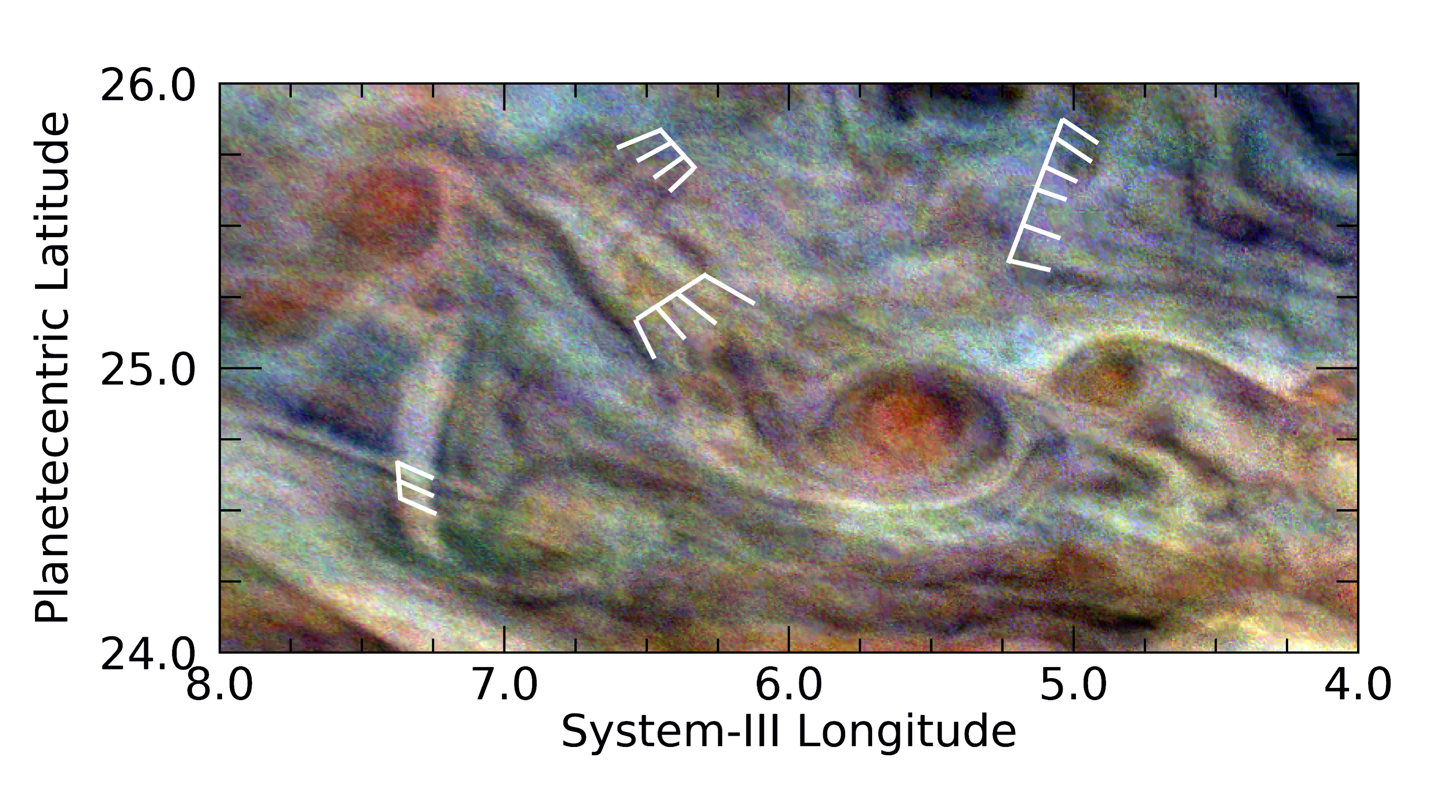


Figure PJ03_109b. JNCE_2016346_03C00109_V01. This figure illustrates many cloud features that include several sets of what appear to be lines of atmospheric flow between and around small anticyclonic vortices that appear reddish in the northern component of the North Temperate Belt (NTBn). The NTB was very turbulent at the time of these observations, following a great NTB disturbance in the preceding months (see Sanchez-Lavega et al. 2017). A triplet of short, dark bands in the top left of this figure can be seen lying across longer bands that appear to be associated with winds.


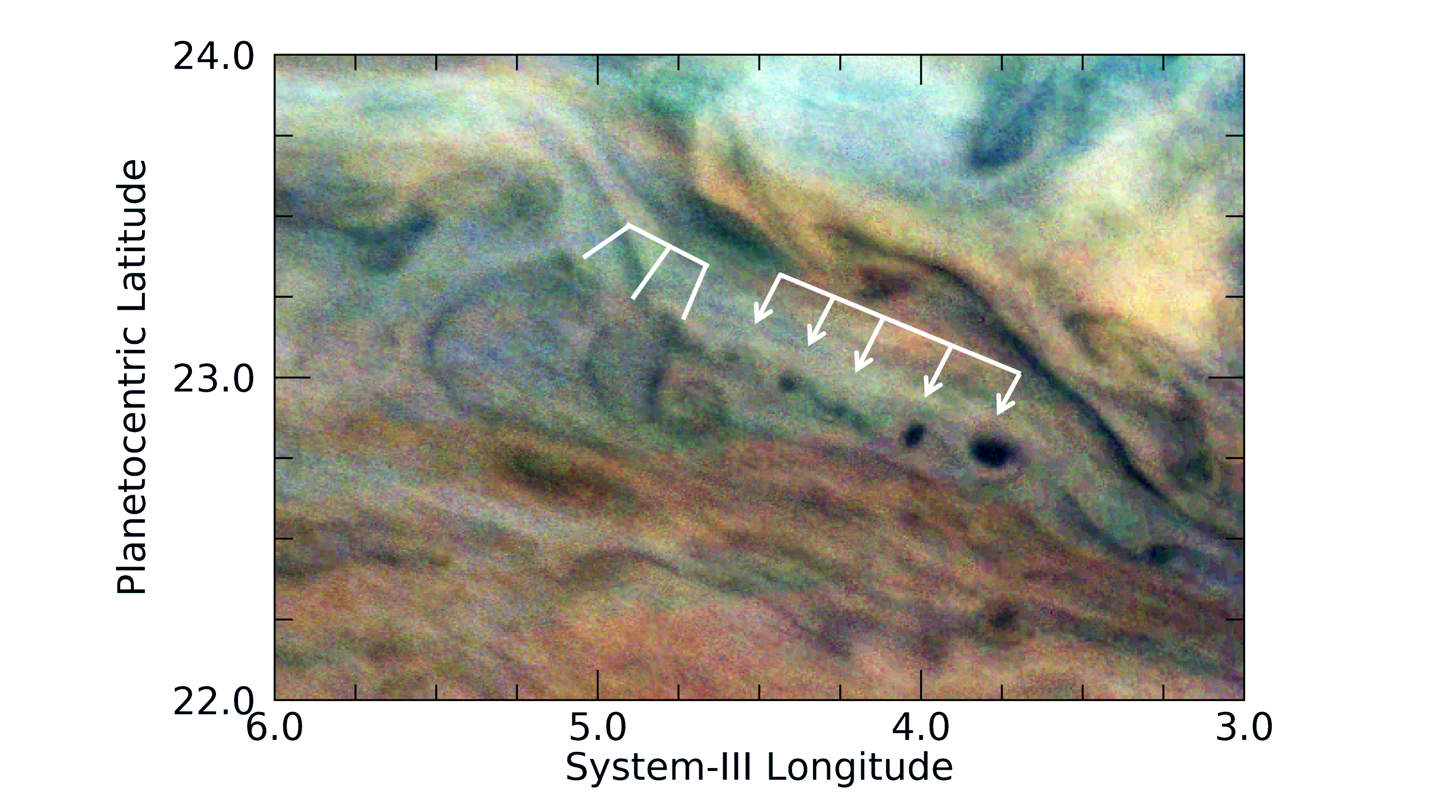


Figure PJ03_109c. JNCE_2016346_03C00109_V01. Three curved wavefronts are indicated. The arrows point to an unusual series of relatively dark circular features, possibly connected dynamically to the wavefronts, because they continue in the same direction and have roughly the same wavelength. These features are located near the boundary between the turbulent northern component and the smooth, orange southern component of the North Temperate Belt.


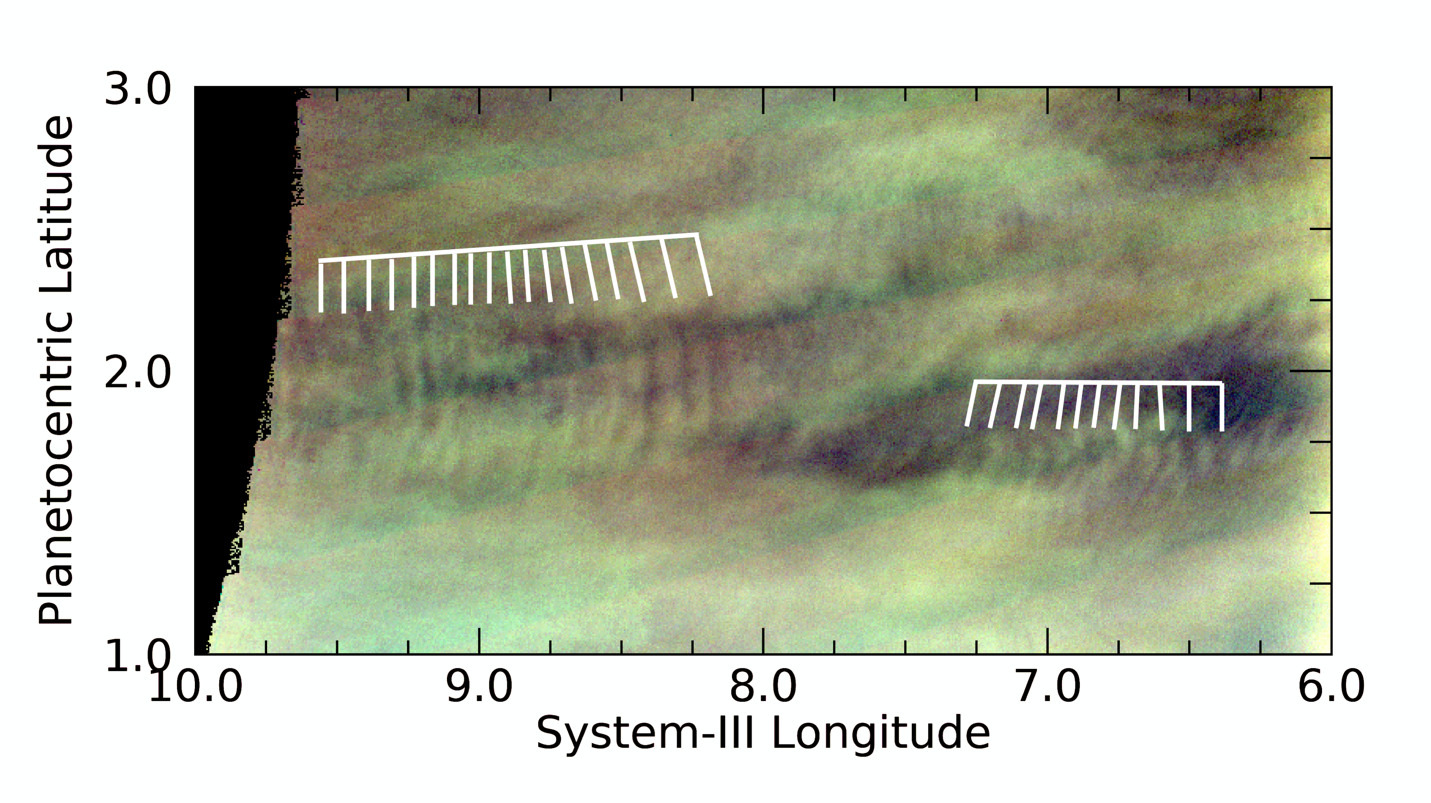


Figure PJ03_111a. JNCE_2016346_03C00111_V01. Two sets of waves are detectable in this figure. In order to see both sets of waves where the background illumination is strongly varying, adjusted histogram equalization has been applied to the image. Some image blockiness due to compression artifacts remains. Nevertheless, the wave packet on the lefta is one of our clearest illustrations of wave fronts that are orthogonal to the direction of the wave train.


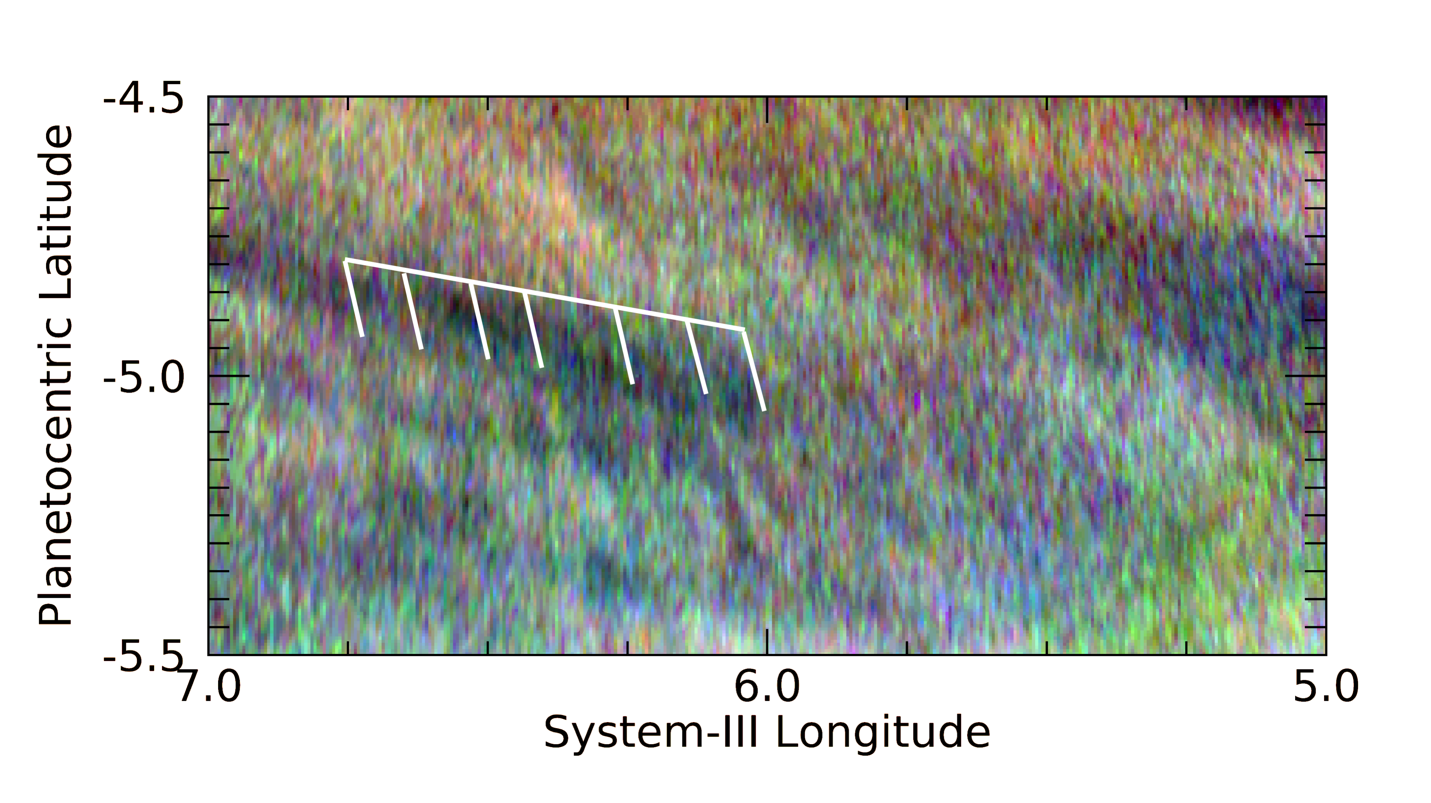


Figure PJ03_111b. JNCE_2016346_03C00111_V01. Some very subtle waves in the Equatorial Zone are detectable above the noise in this image.


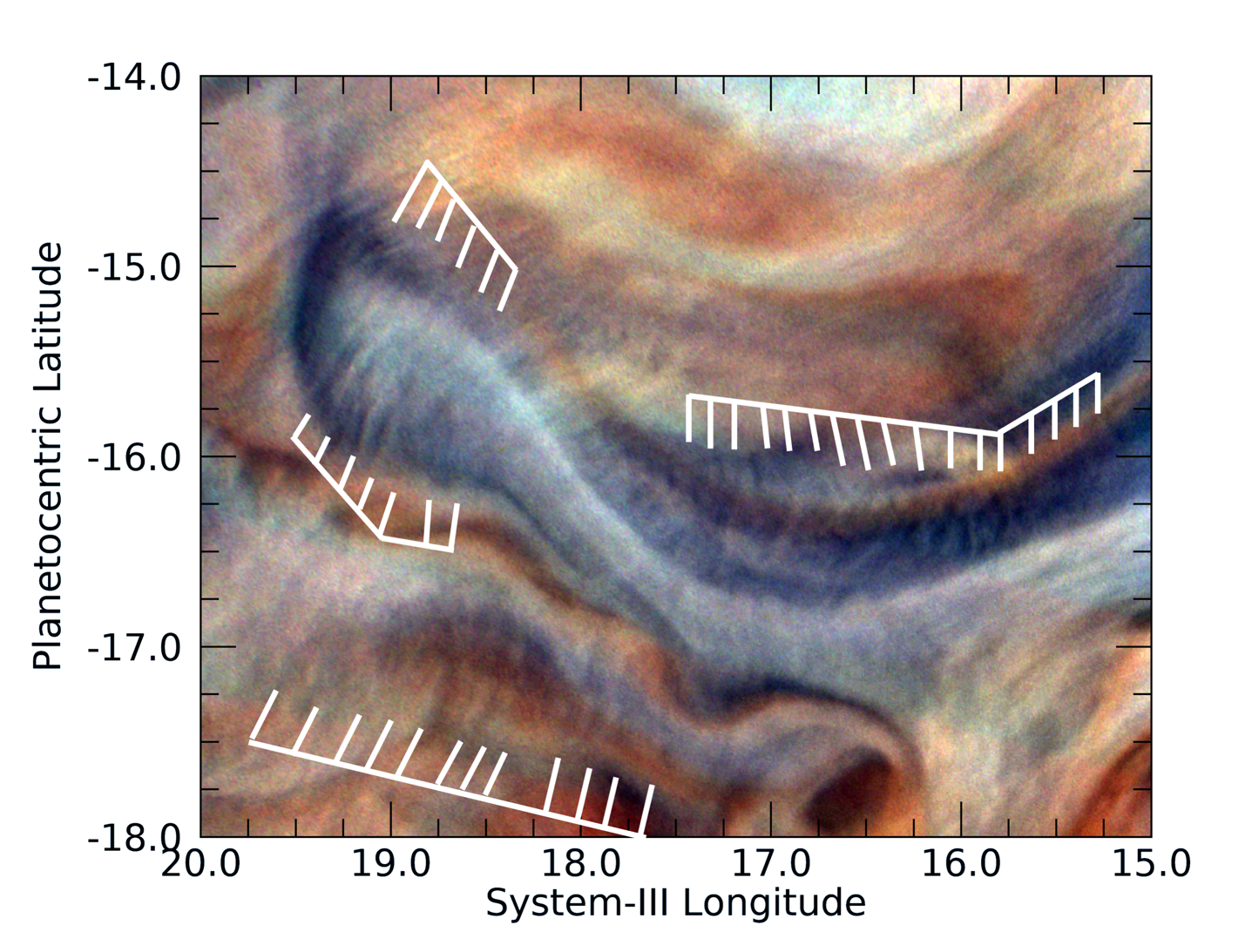


Figure PJ03_114. JNCE_2016346_03C00114_V01. Wave-like features appear along and extending from the curves periphery of this darker feature in the South Equatorial Belt (SEB).


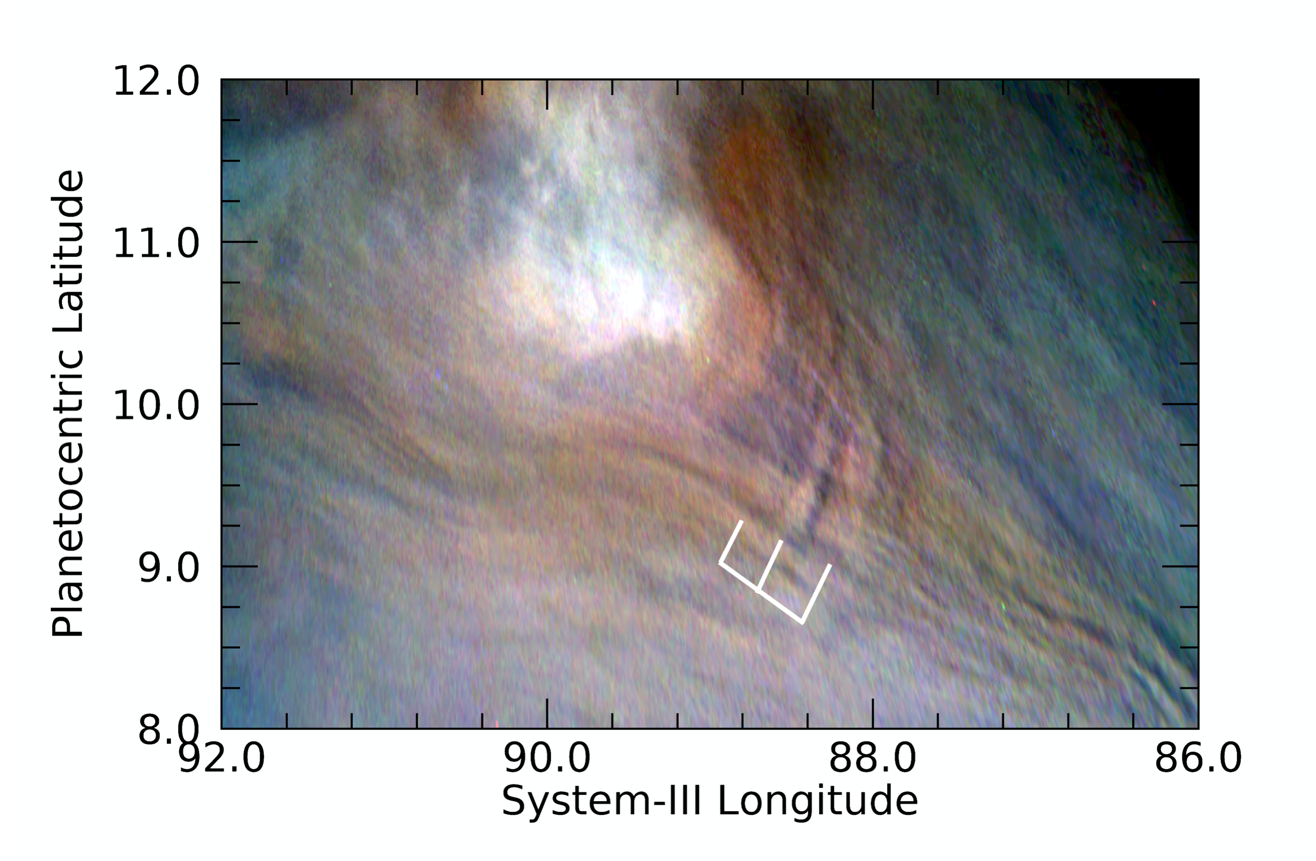


Figure PJ04_102. JNCE_2017033_04C00102_V01. Waves are prominent topographically from their prominent shadows, appearing orthogonal to the dark and light streaks that are presumed to be lines of flow that are aligned with the cyclonic wind gradient in the North Equatorial Belt (NEB). The dark lines that appear to trace atmospheric flow appear to go not only around the bright feature, which is likely to be a convective plume, but over it. Thus, the prominent orthogonal waves are most likely to be only example of a true downslope lee wave we have detected in the atmosphere of Jupiter from JunoCam.


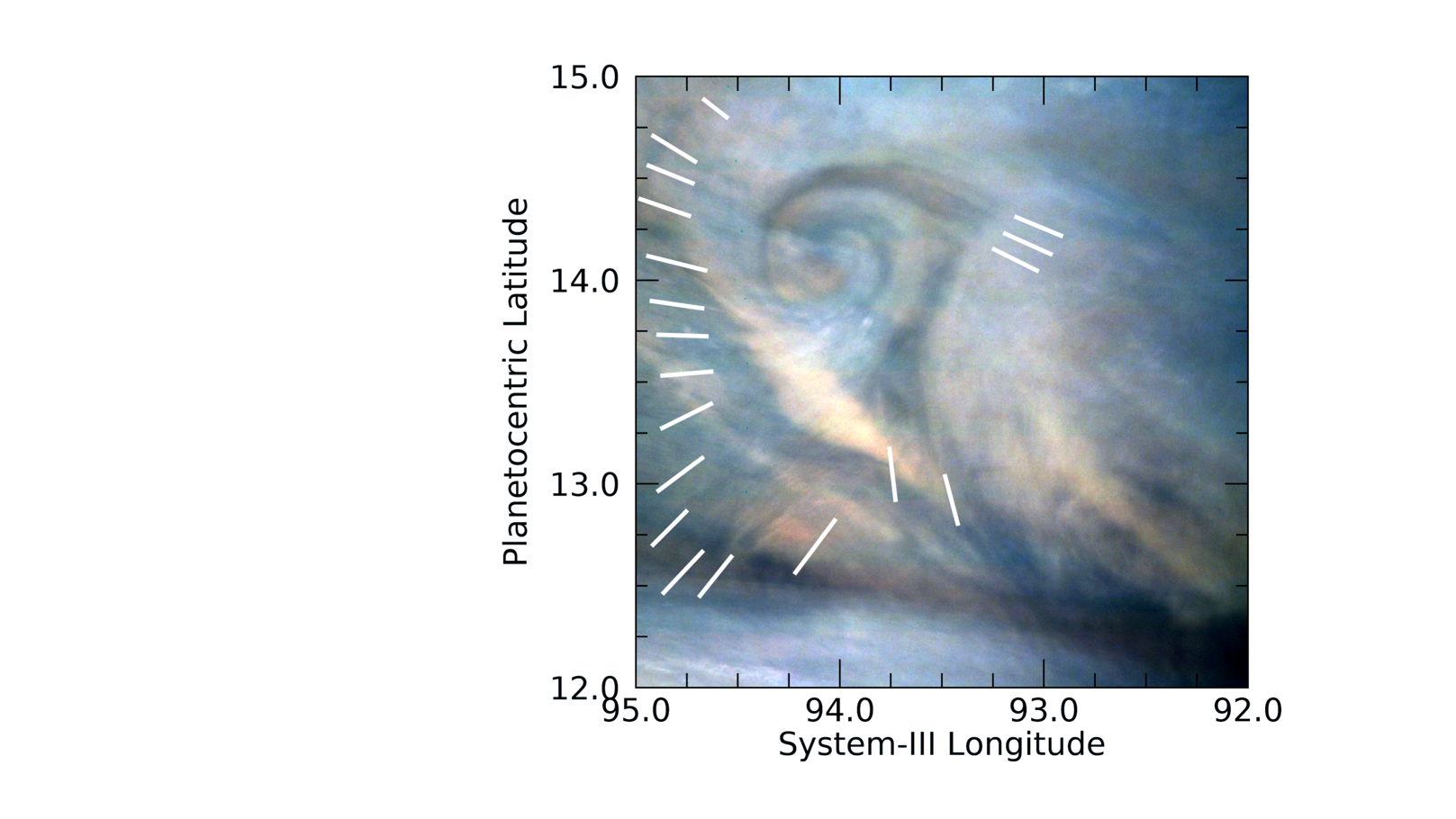


Figure PJ04_103a. JNCE_2017033_04C00103_V01. Wave fronts are apparent in the periphery of a remarkable cyclonic spiral. The spiral is a bright feature in the complex system of disturbances (‘rifts’) in the North Equatorial Belt.


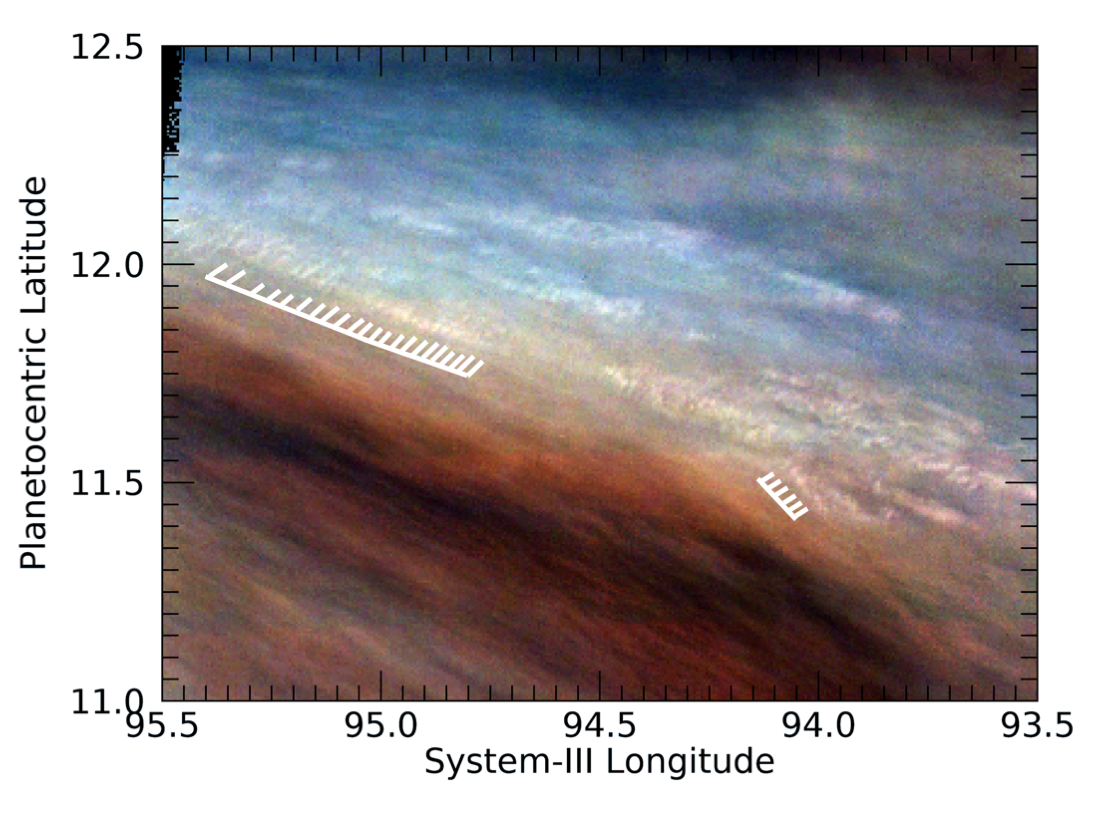


Figure PJ04_103b. JNCE_2017033_04C00103_V01. This figure reveals extensive wave packets controlling small, bright cloud features in a bright patch of the complex ‘rift’ system in the North Equatorial Belt (NEB). White grids identify packets in which the individual cloud features are resolved clearly. These packets are aligned with the cyclonic wind gradient across the NEB and roughly orthogonal to the waves; the wind gradient is also suggested by many faint striations with similar alignment in the surrounding bright area and in the dark brown area to the south

(see the report on JunoCam images from PJ4 by co-author Rogers at

<https://britastro.org/sites/default/files/Juno-PJ4_Report_JHR_0.pdf>).


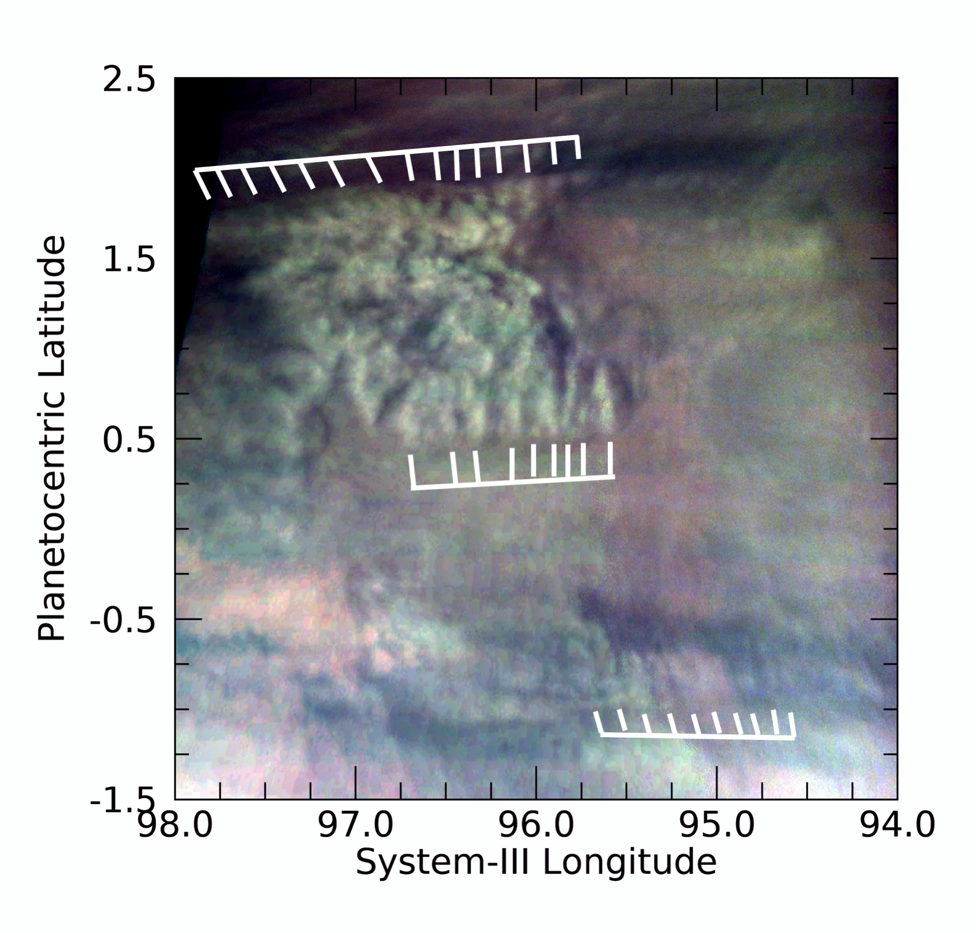


Figure PJ04_104a. JNCE_2017033_04C00104_V01. Multiple sets of wave trains can be identified in this image of a region in the EZ. (Note: this color scheme was adopted to minimize the appearance of artefactual green and red stripes across the frame, although a residual still remains.)


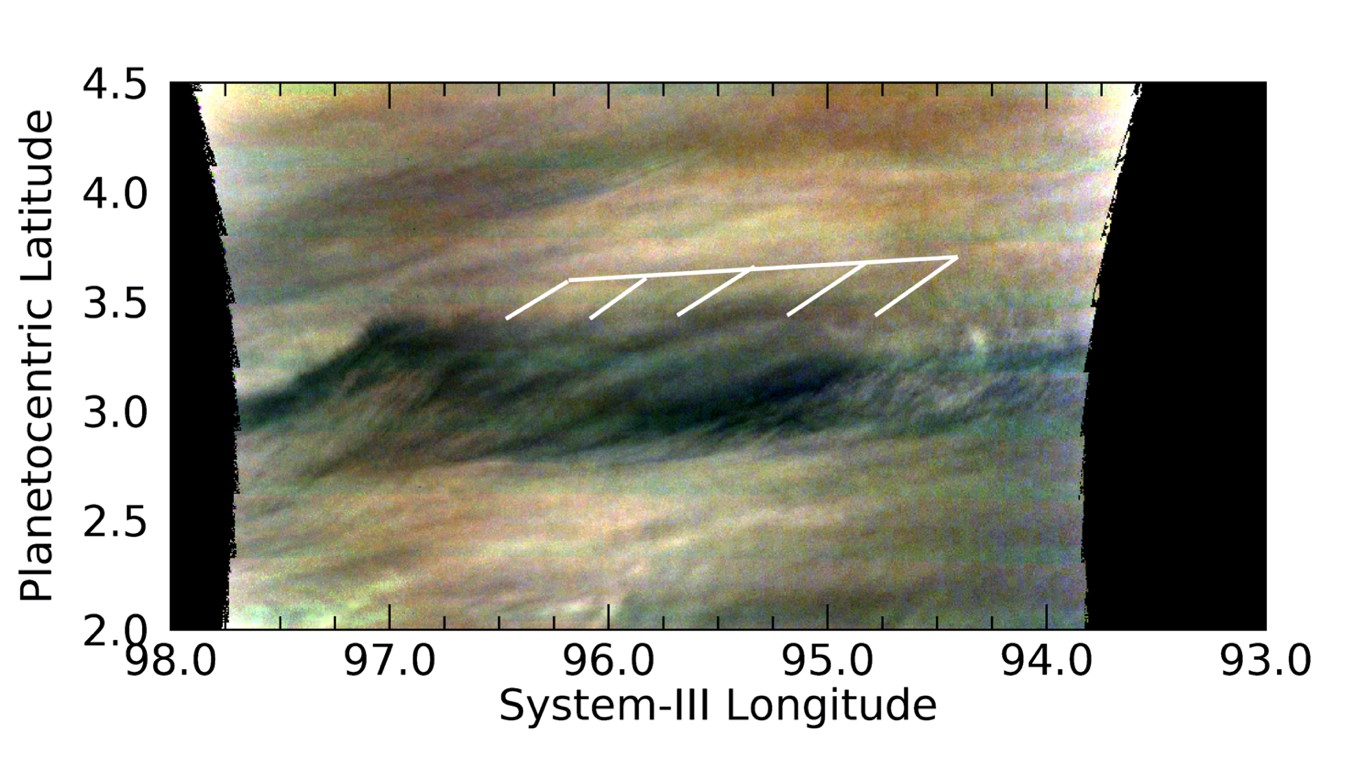


Figure PJ04_104b. JNCE_2017033_04C00104_V01. Long, wave-like patterns can be seen in this image overlapping a darker feature in the Equatorial Zone.


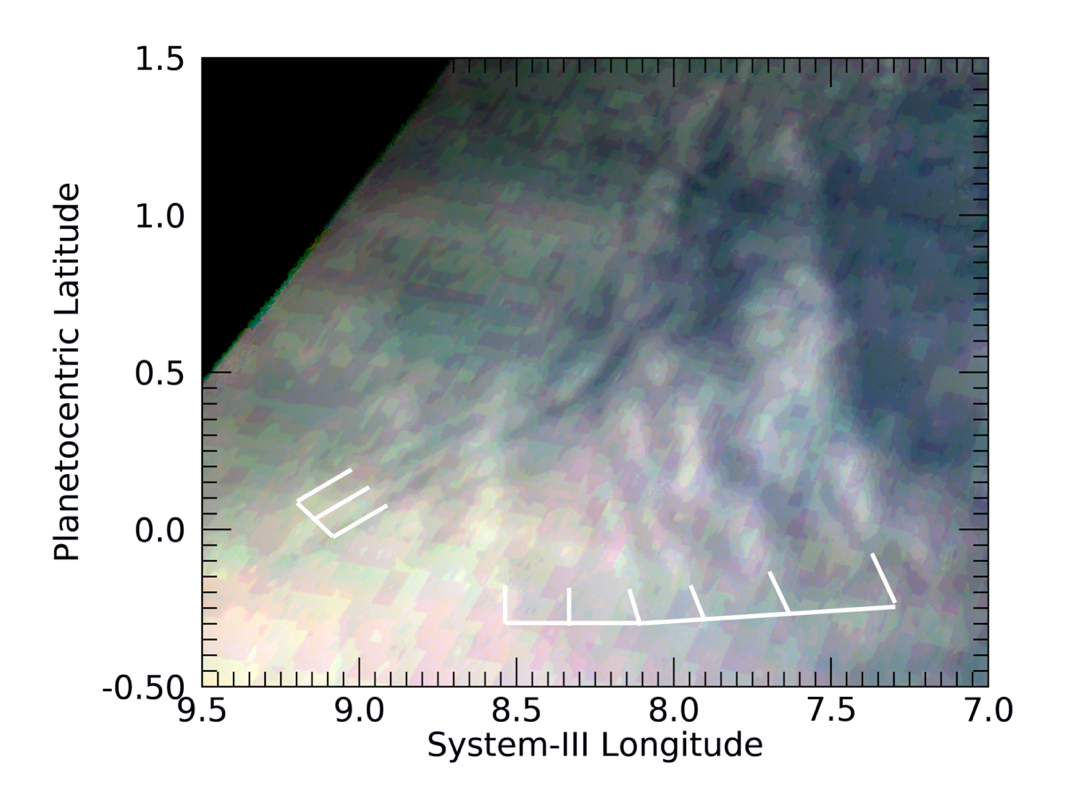


Figure PJ05_107a. JNCE_2017086_05C00107_V01. Two sets of overlapping waves are detected in the Equatorial Zone, both with wavefronts that are longer than the wave packets. (Note: as in Figure PJ04_104a, this color scheme was adopted to minimize the appearance of artefactual green and red stripes across the frame.)


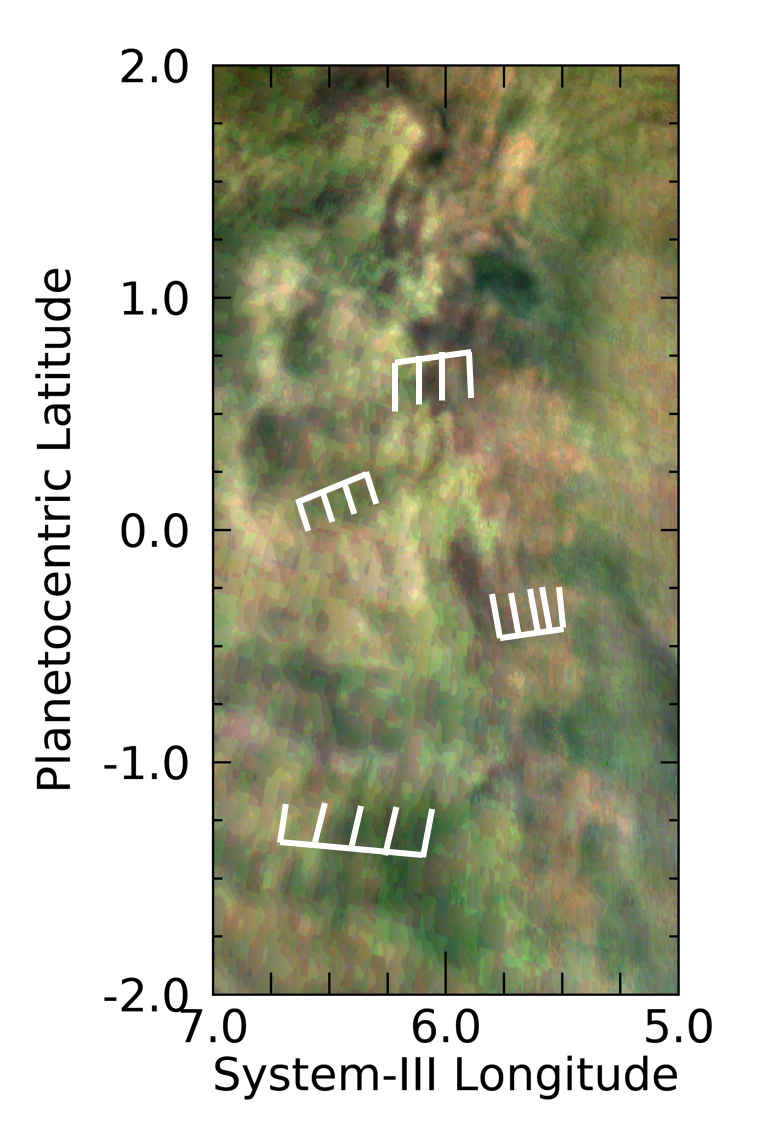


Figure PJ05_107b. JNCE_2017086_05C00107_V01. Several sets of waves can be detected in this area of the Equatorial Zone.


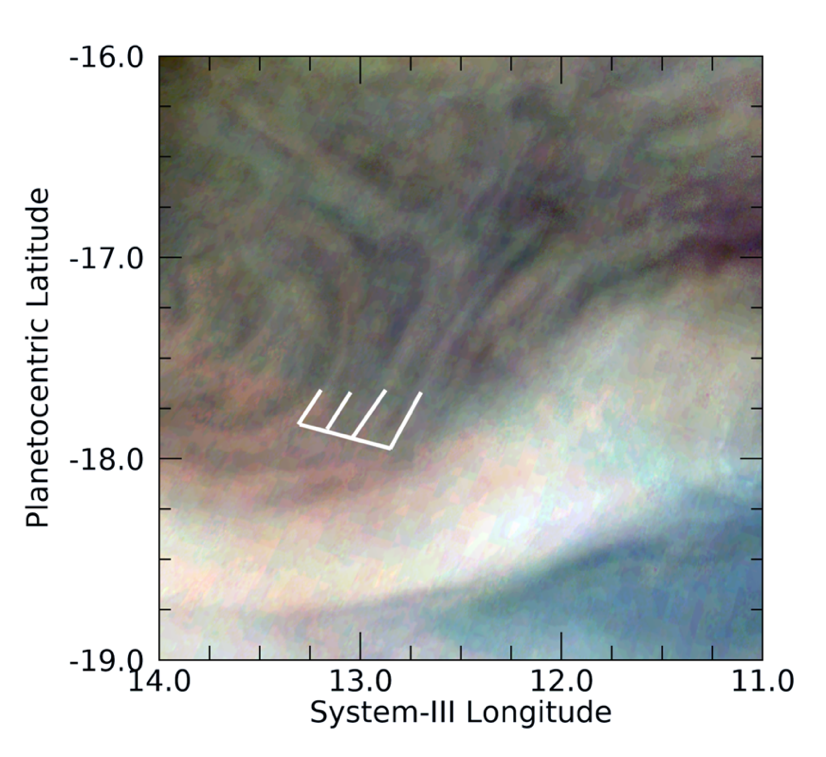


Figure PJ05_108. JNCE_2017086_05C00108_V01. Parallel bands, likely representing a flow pattern, are located in a pale strip of the southern component of the South Equatorial Belt (SEBs). They are located between a weak cyclonic eddy on the left and a bright wave-like streak aligned with the SEBs retrograde jet, running near the bottom edge of the figure. These bands are similar to those in the Figure PJ03_109b.


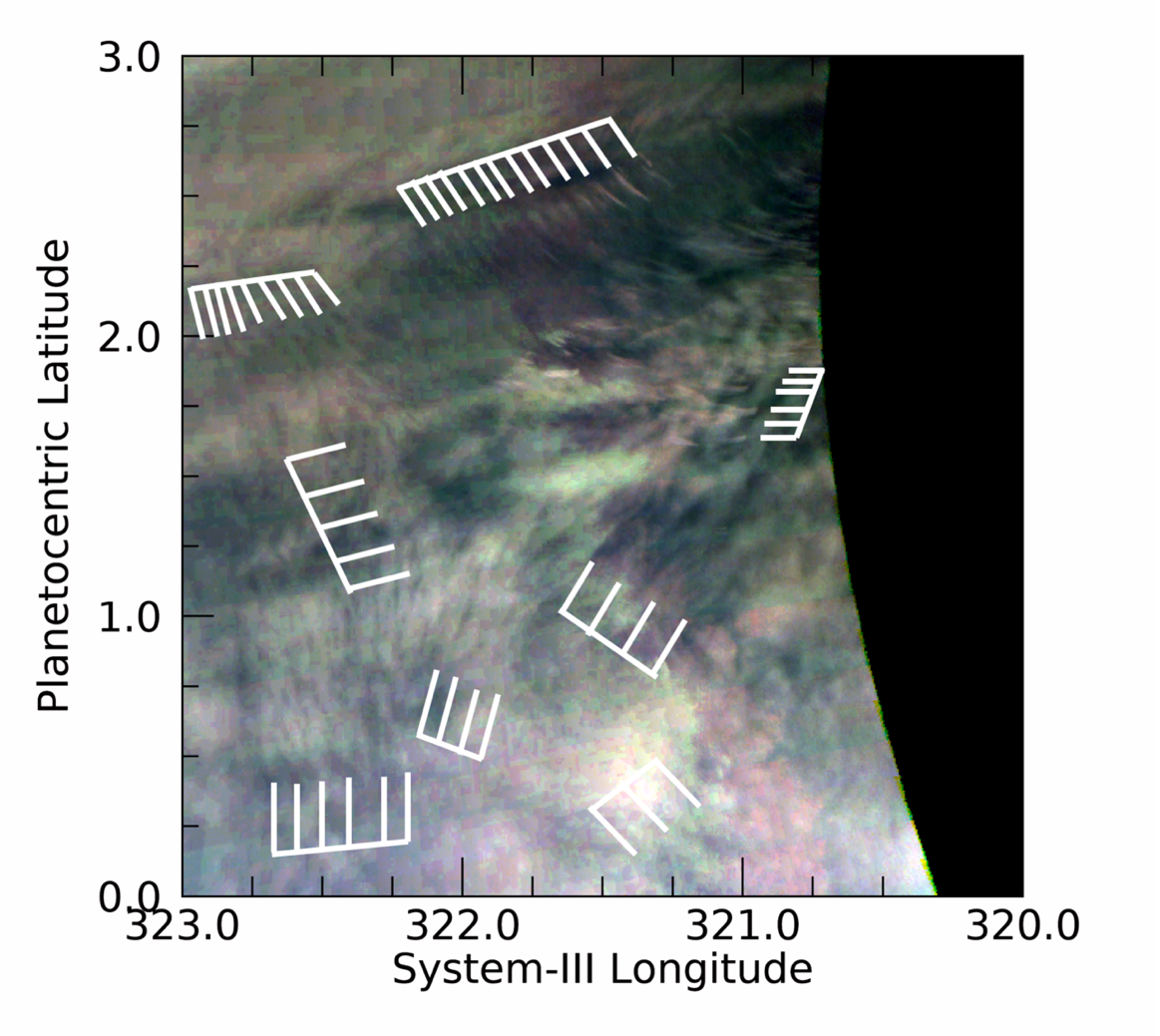


Figure PJ06_115. JNCE_2017139_06C00115_V01. This figure illustrates multiple sets of often overlapping and orthogonal waves present in the Equatorial Zone. (Note: as in Figures PJ04_104a and PJ05_107a, this color scheme was adopted to minimize the appearance of artefactual green and red stripes across the frame.)


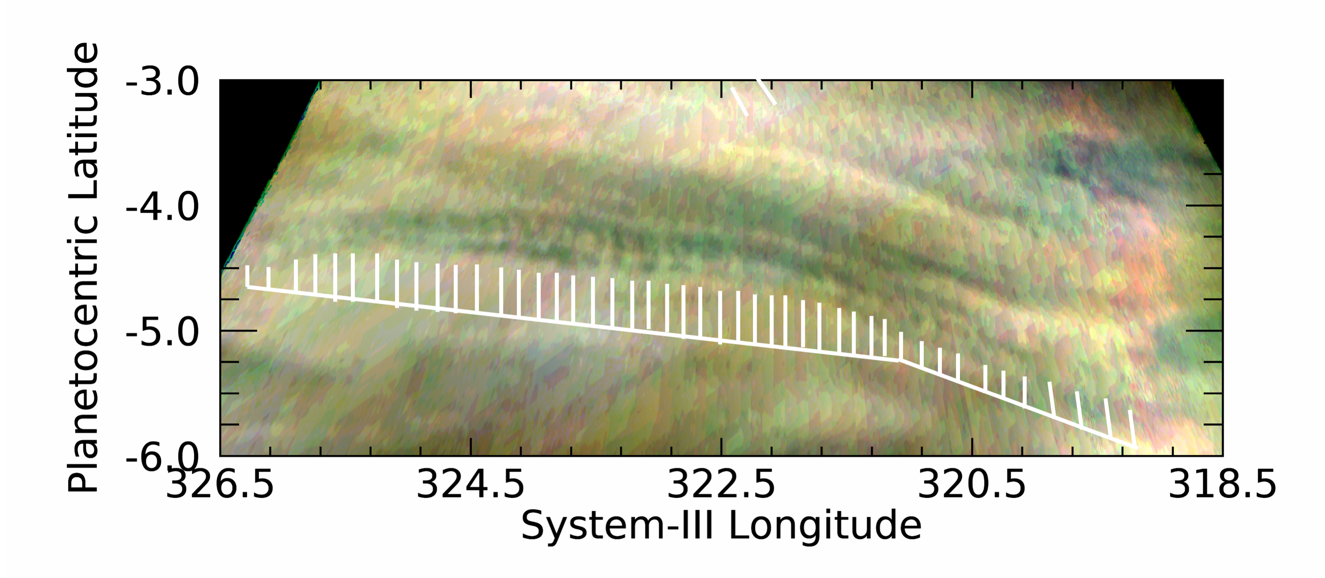


Figure PJ06_115. JNCE_2017139_06C00115_V01. This figure illustrates a series of waves appearing most prominently as “knots” on a narrow, linear bright cloud bank in the Equatorial Zone.


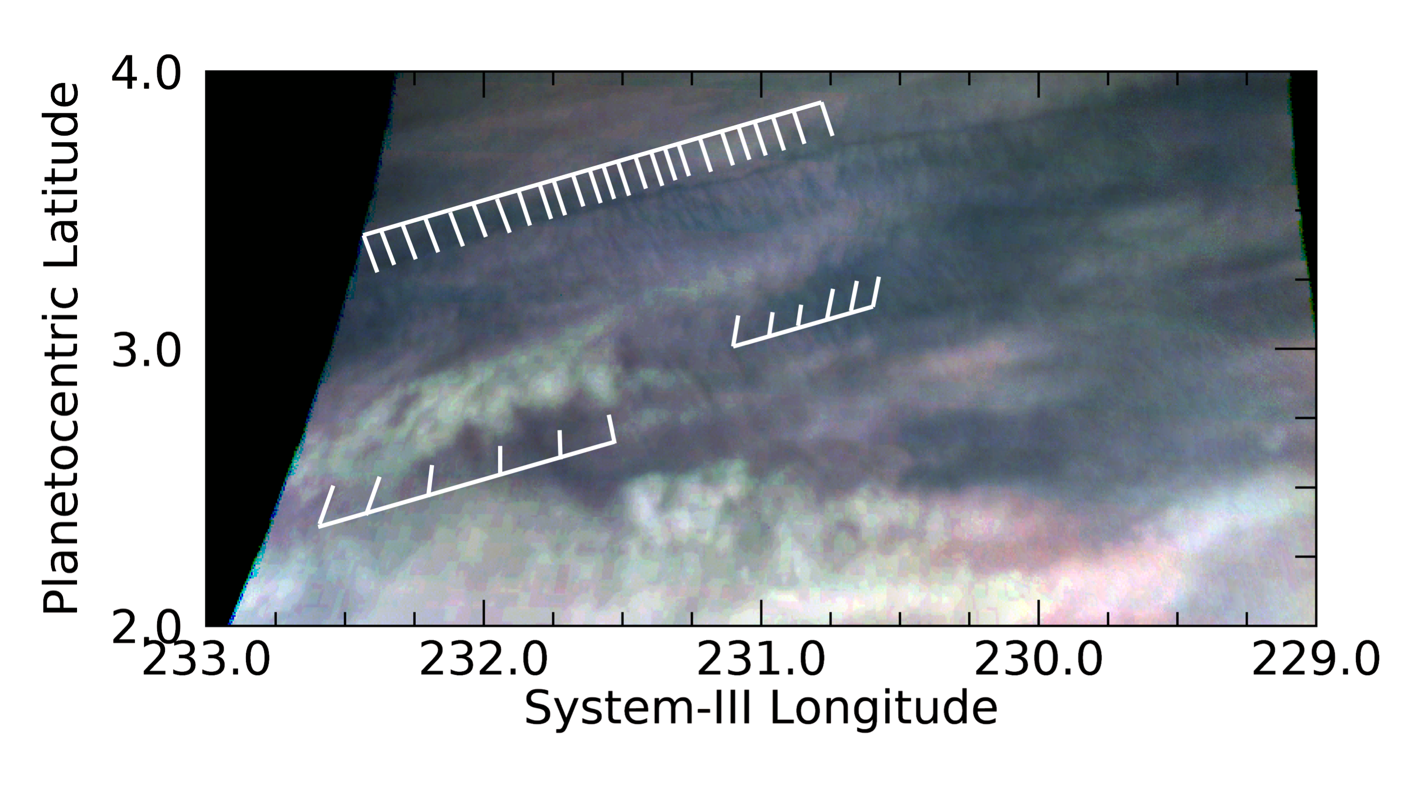


Figure PJ07_57. JNCE_2017192_07C00057_V01. Several wave packets can be identified in this area of the Equatorial Zone.


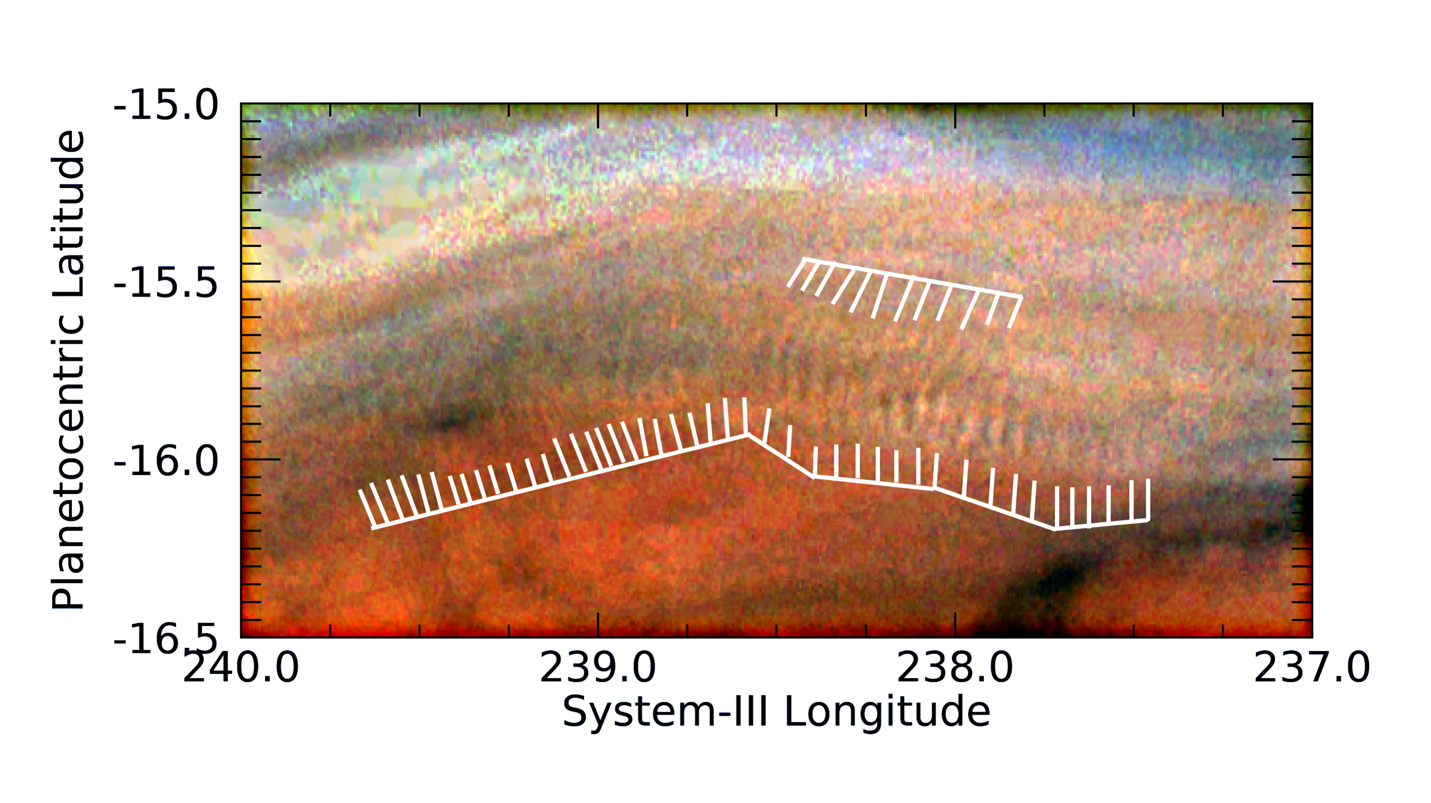


Figure PJ07_60. JNCE_2017192_07C00060_V01. This shows two interfering sets of waves in the northern part of the GRS. This is the same wave feature as shown by Sanchez-Lavega et al. (2018) in their Figure 8. Besides being color-stretched, this image was also unsharp-masked


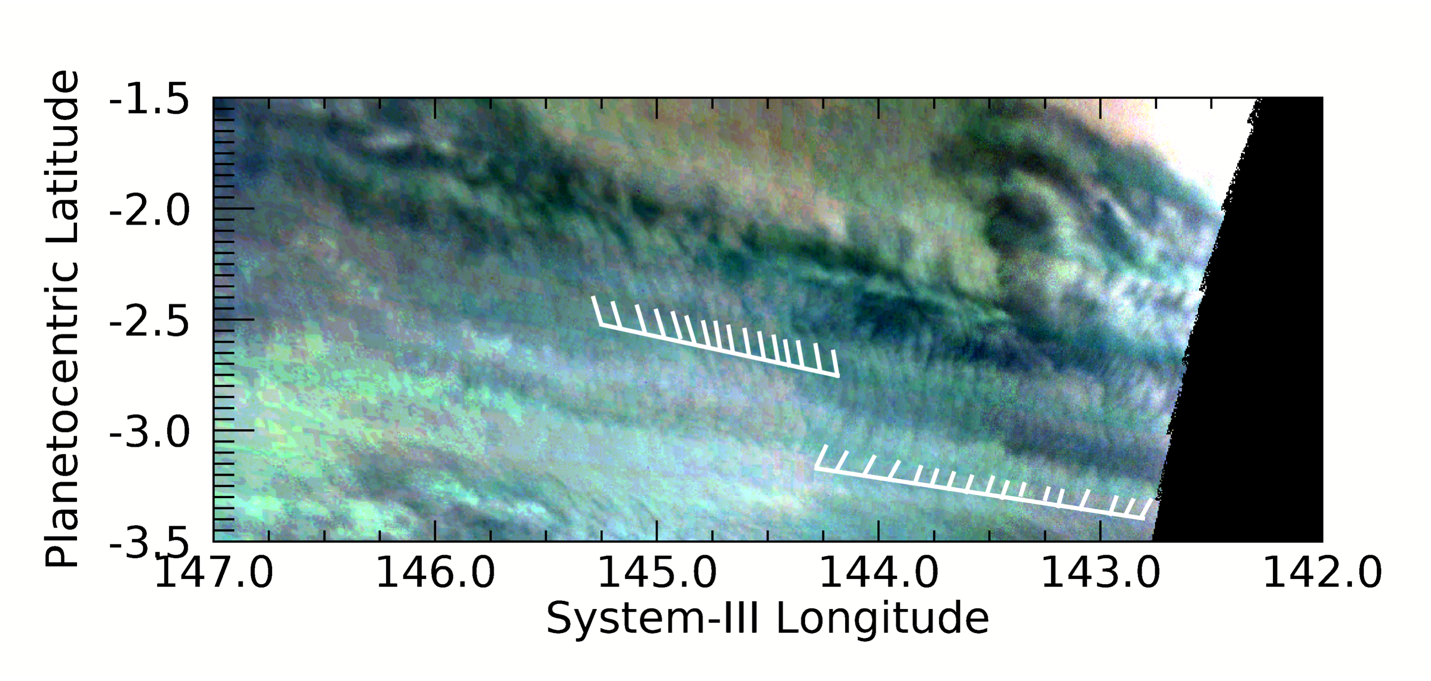


Figure PJ08_116a. JNCE_2017244_08C00116_V01. This image shows evidence for multiple sets of waves, the most prominent two of which are illustrated by the grids.


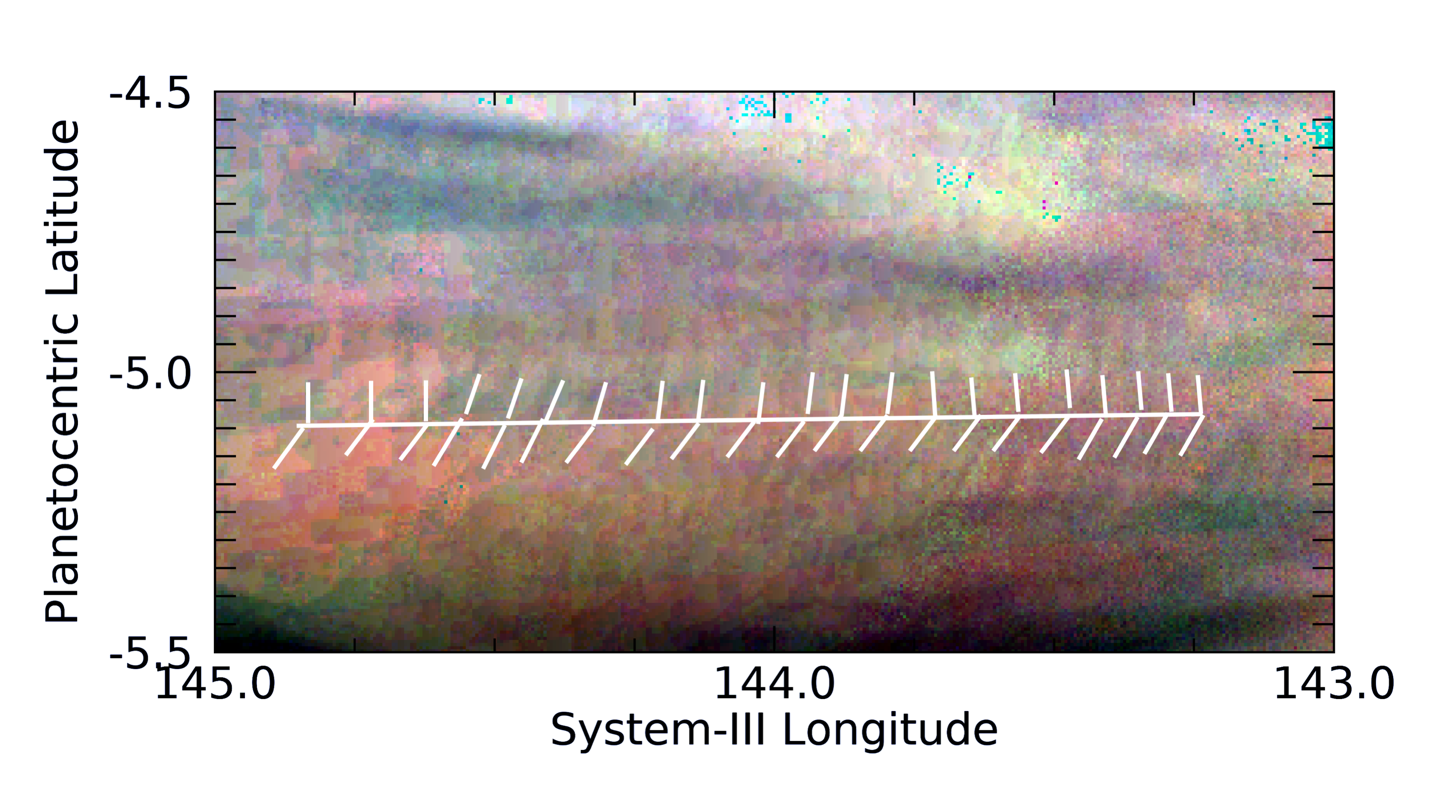


Figure PJ08_116b. JNCE_2017244_08C00116_V01. Two tilts appear to be the characteristic of these very subtle waves that are just detectable above the compression noise of this image.


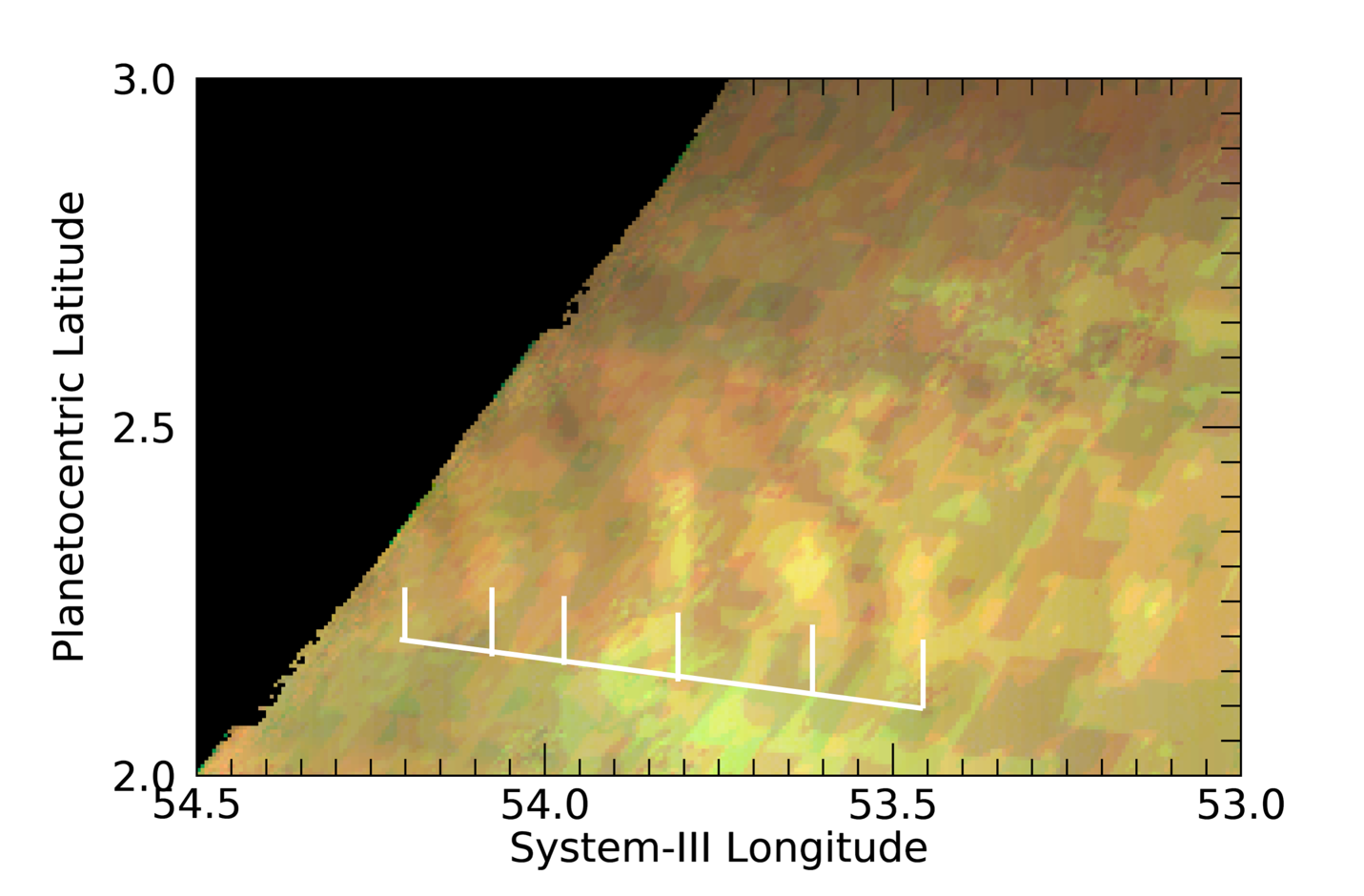


Figure PJ09_85. JNCE_2017297_09C00085_V01. Waves can be detected above the compression noise of this image.


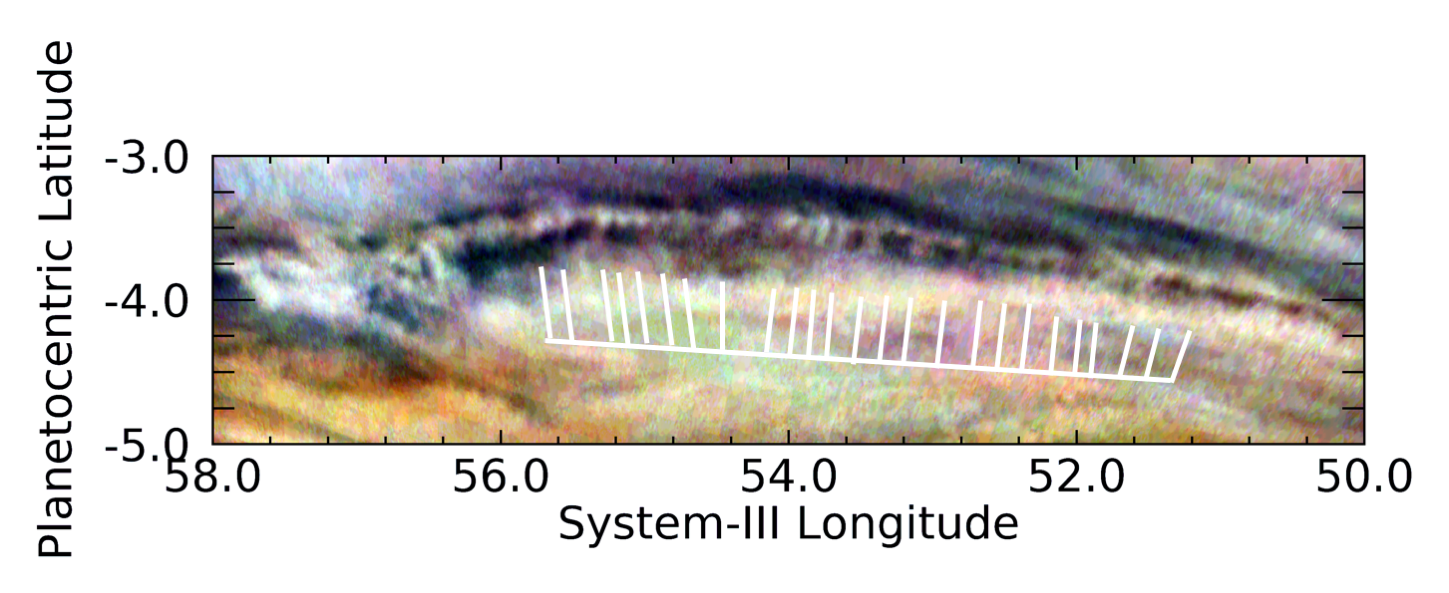


Figure PJ09_88. JNCE_2017297_09C00088_V01. The grid points to discrete white clouds that appear like ‘knots’ in the narrow ‘rope’ that forms a series of waves that follow a long, curved wave packet.


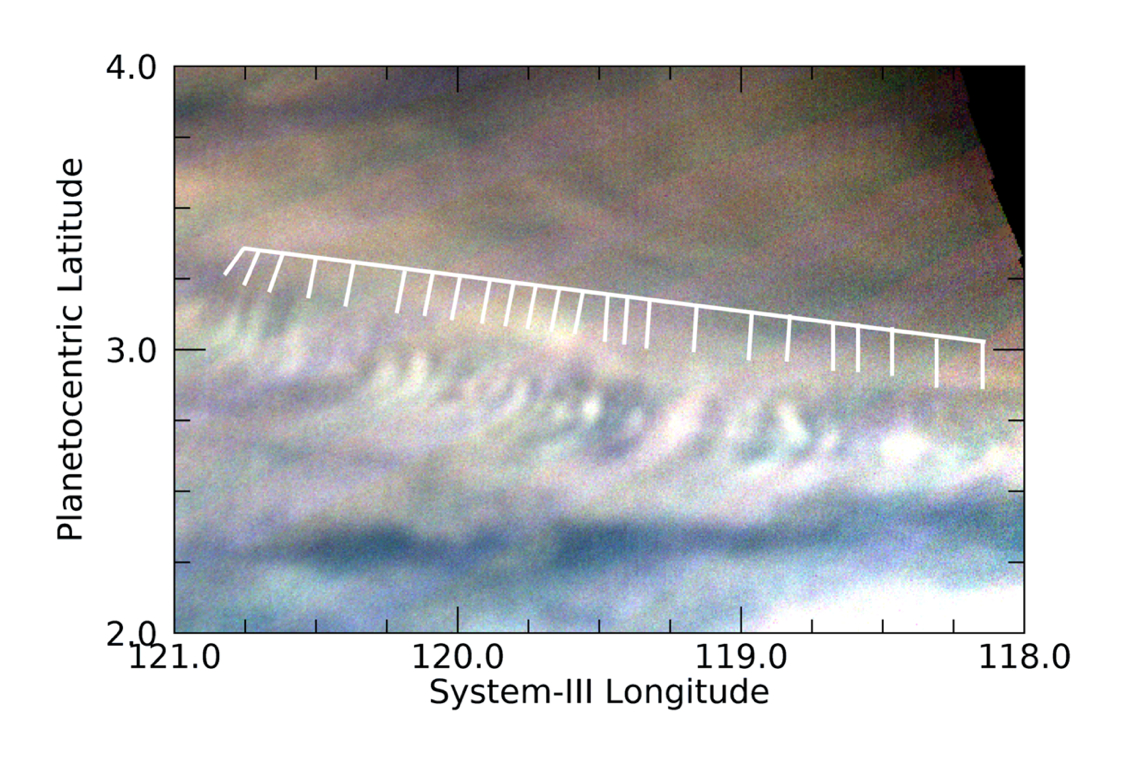


Figure PJ10_28b. JNCE_2017350_10C00028_V01. A series of discrete, bright cloud features comprises a wave packet in the Equatorial Zone. Unlike the wave packet shown in Fig. PJ09_88, there is not such an obvious bright ‘line’ connecting them


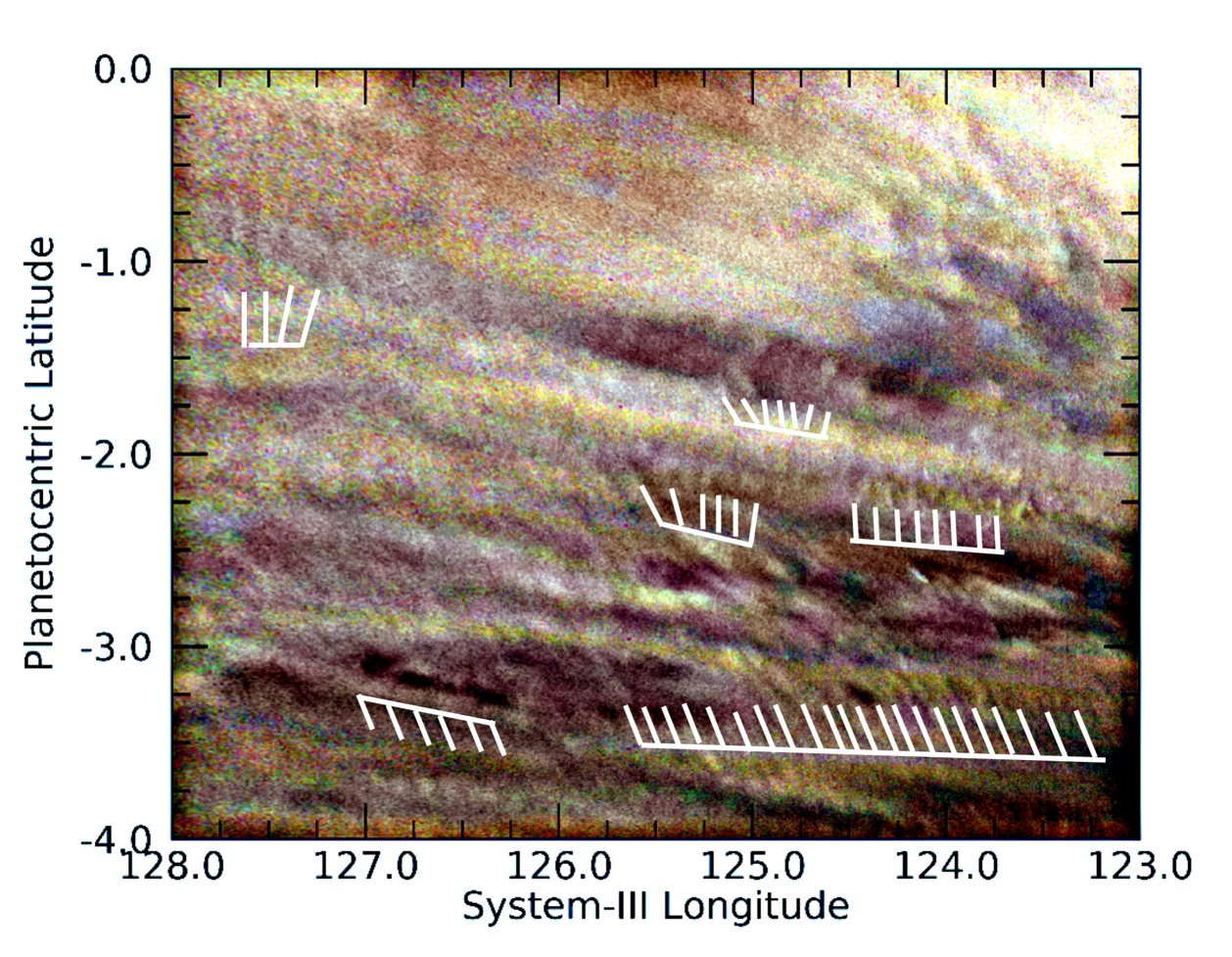


Figure PJ10_30a. JNCE_2017350_10C00030_V01. Several wave trains are detectable above the noise in this image, some of which are indicated by the white grid lines. We have numerically suppressed artificial colored striping across the field. (In order to show waves in both the light and dark regions of this image, we have used unsharp masking.)


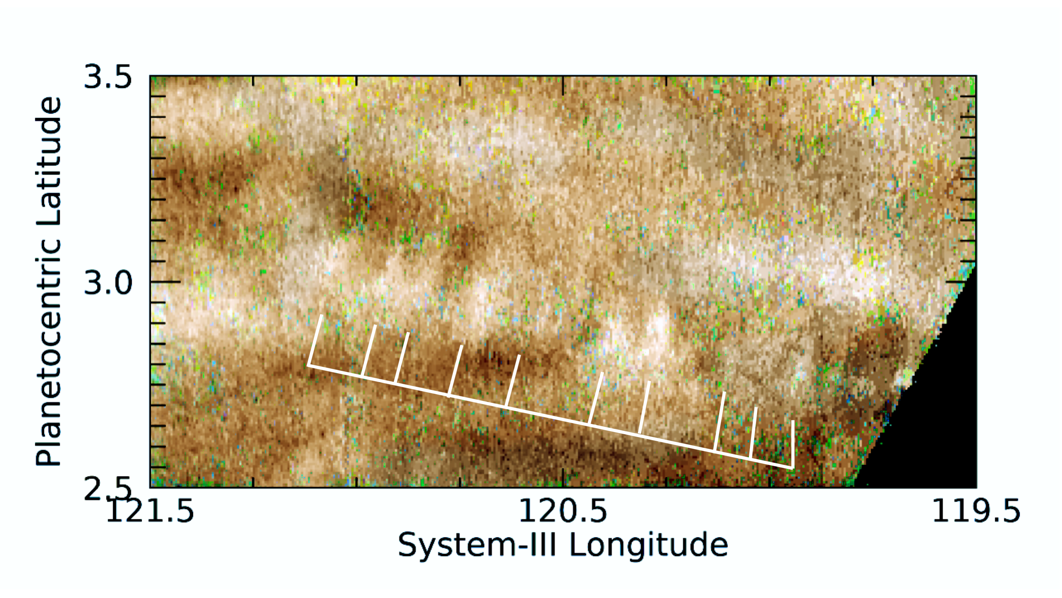


Figure PJ10_30b. JNCE_2017350_10C00030_V01. Some very subtle waves remain detectable in this image after substantial processing to remove green striping artefacts.


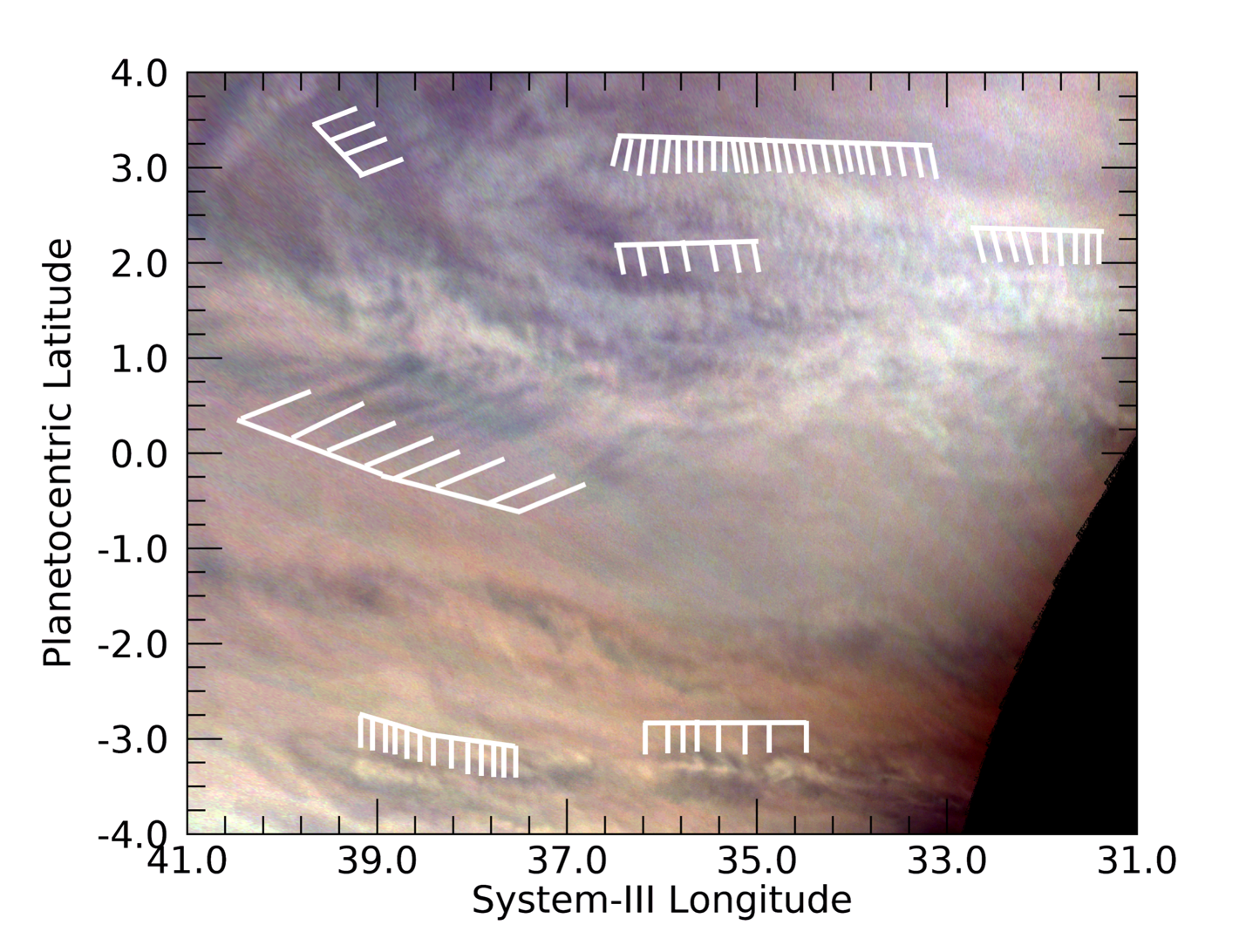
Figure PJ11_20. JNCE_2018038_11C00020_V01. Several waves trains, some of which are overlapping, are evident in this image. We identify several of these by grids of white lines.


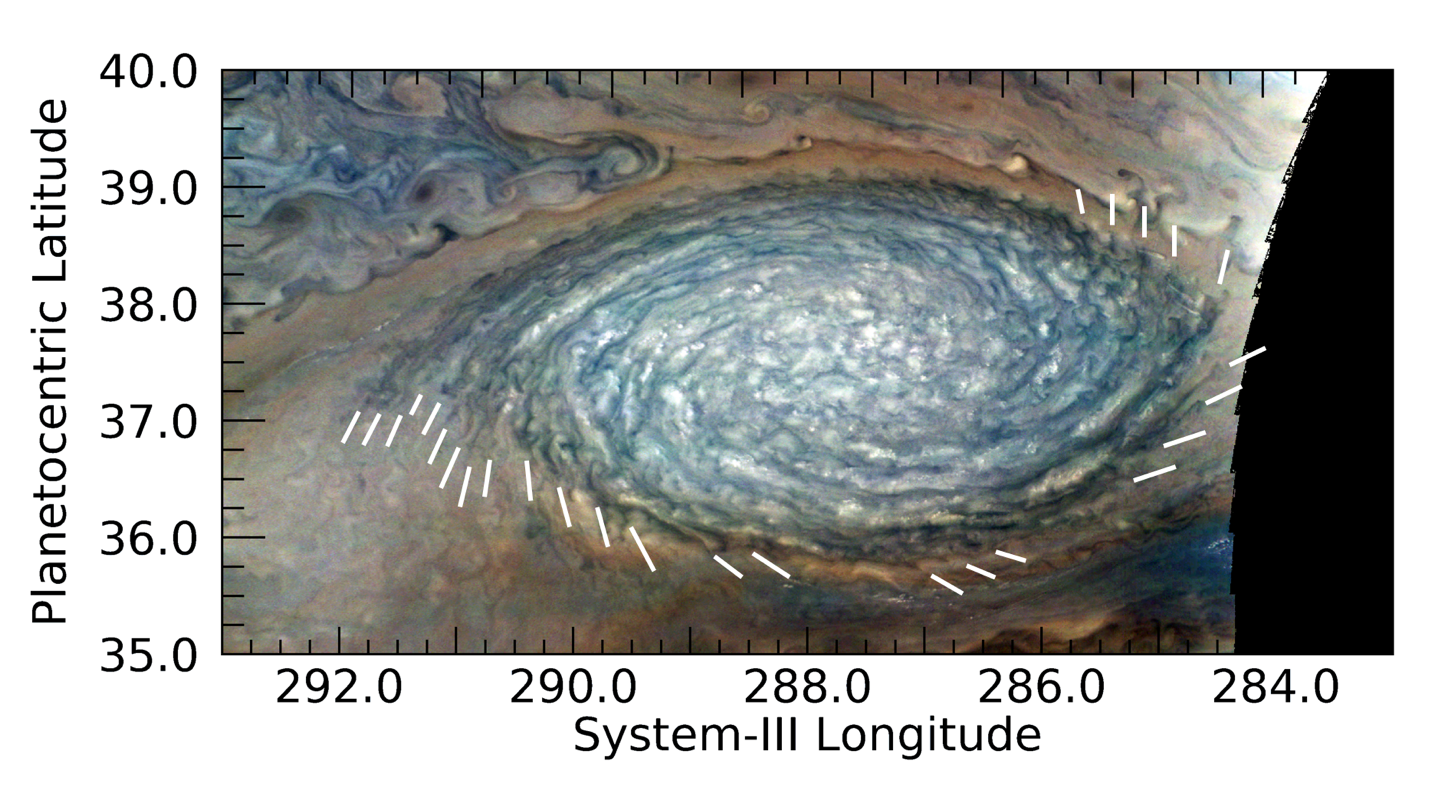


Figure PJ12_85. JNCE_2018001_12C00085_V01. A series of bands can be detected that are associated with the ends of the internal spiral structures within a prominent anticyclonic white oval. Reminiscent of bands of clouds in terrestrial hurricanes, they are more or less regularly spaced. Some individual discrete white clouds can be seen in the ridges that define the spiral structure, but they are insufficiently resolved to determine whether a regular spacing exists between them.


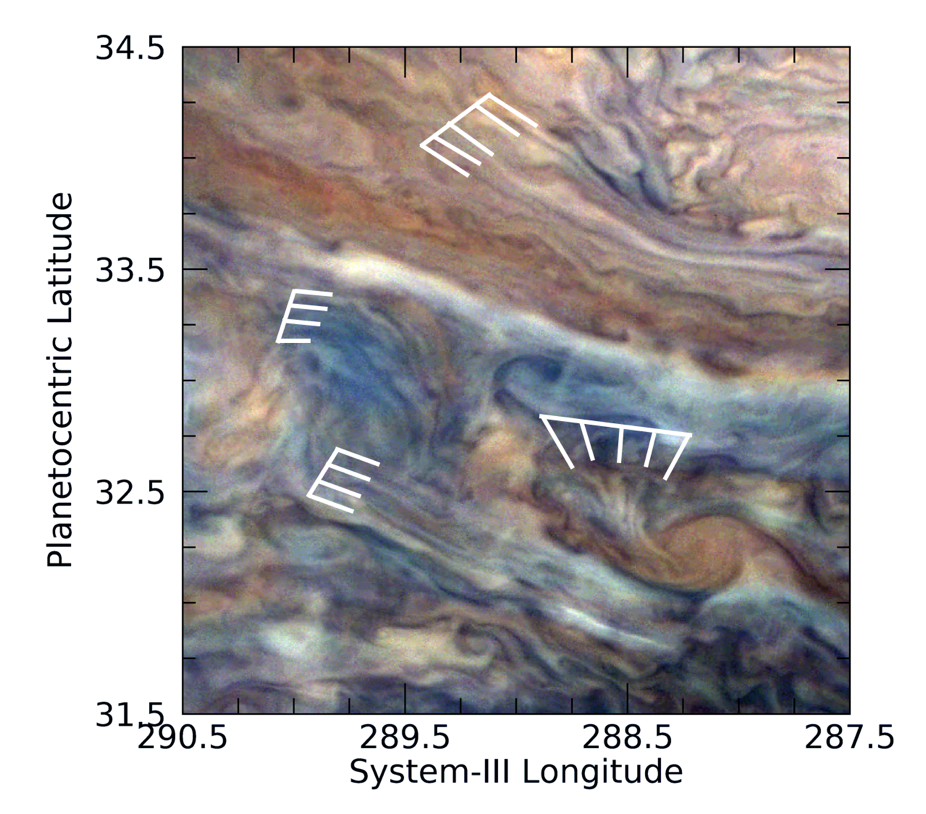


Figure PJ12_86b. JNCE_2018001_12C00086_V01. Multiple examples of aligned cloud streaks with very different orientations are visible in this turbulent part of the North North Temperate Belt. These are similar to those, for example, in Figs. PJ3_109c and PJ5_108, showing that they are common.


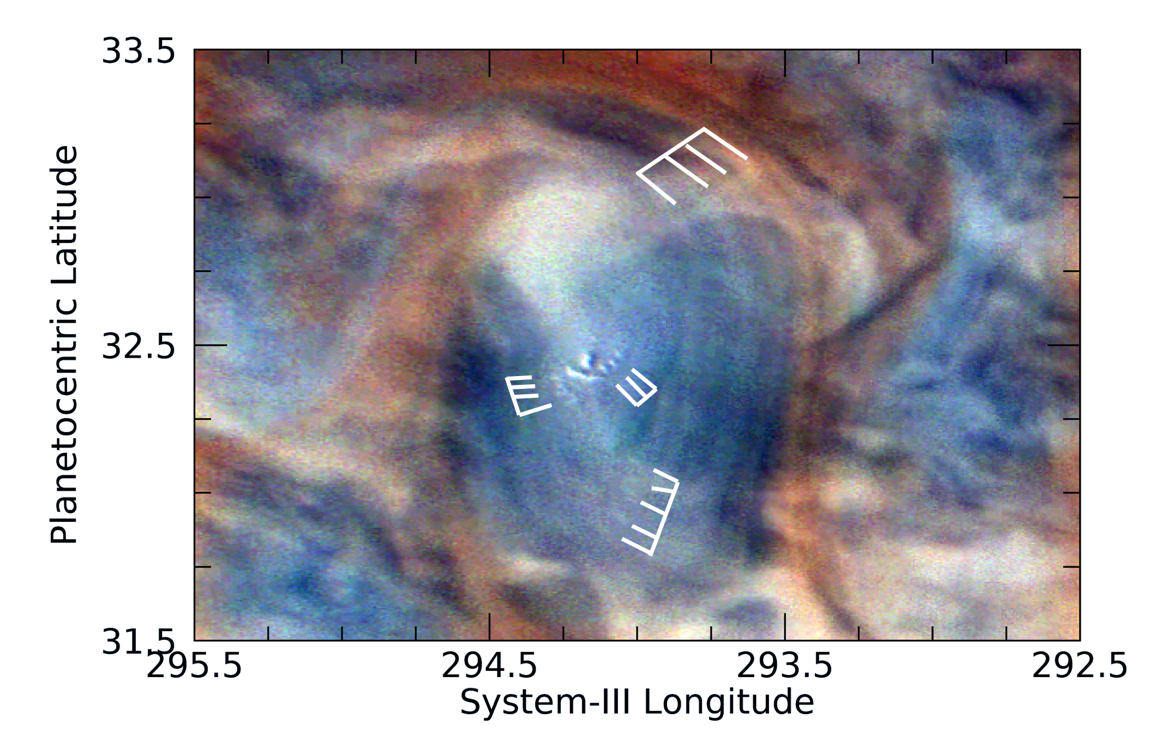


Figure PJ12_86c. JNCE_2018001_12C00086_V01. Close examination of this region, also in the North North Temperate Belt and due west of the area in Fig. PJ12_86a, shows not only cloud streaks but also much finer-scale features. The most unexpected were the few small, bright clouds, all of which subtend shadows and extend higher than the surrounding darker region. Verifying that they are not an artefact in the imaging, we note that they are present – although at lower resolution – in the image taken prior to this one (JNCE_2018001_12C00085_V01).


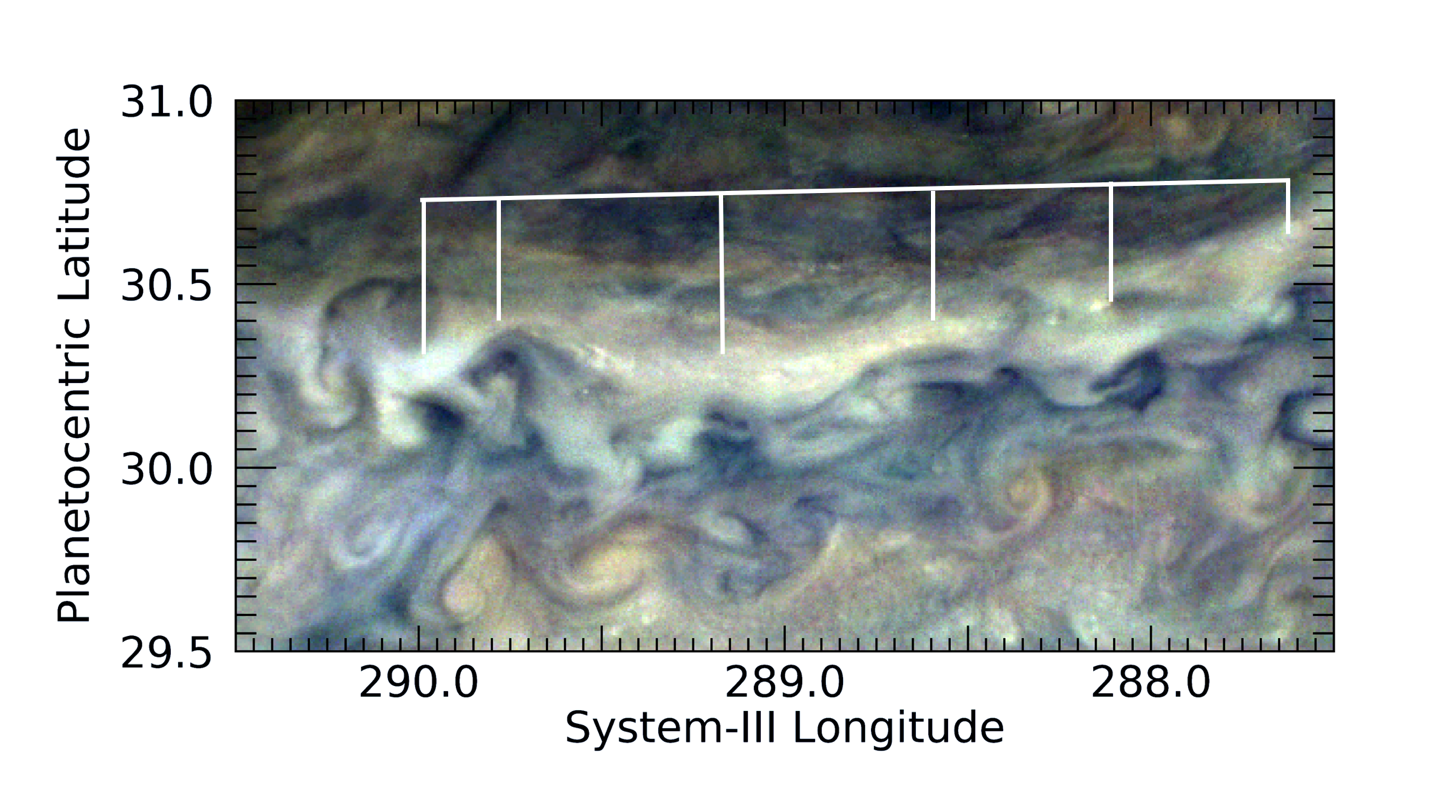


Figure PJ12_86d. JNCE_2018091_12C00086_V01. Prominent repeated patters are seen along the southern edge of an unusual white band located at the turbulent boundary between the northern and southern components of the North Temperate Zone. It is not clear that these can be classified as waves.


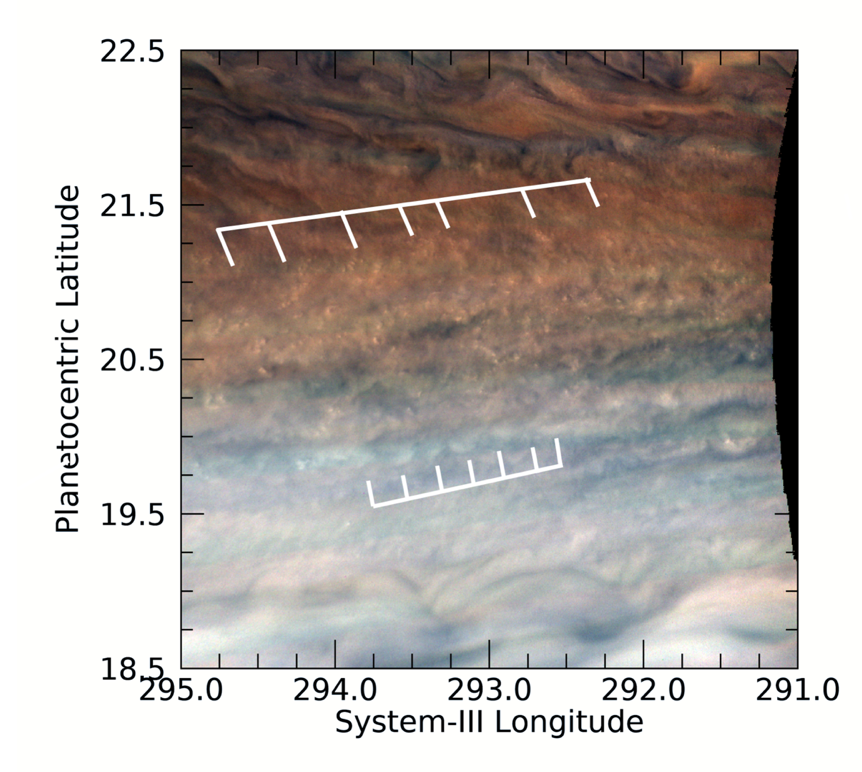


Figure PJ12_87b. JNCE_2018091_12C00087_V01. Repeated wave-like features (lower grid) and regular clusters of clouds (upper grid) are detectable in this image. The latter probably represent a pattern that barely meets our criterion for consideration as a wave-like feature.


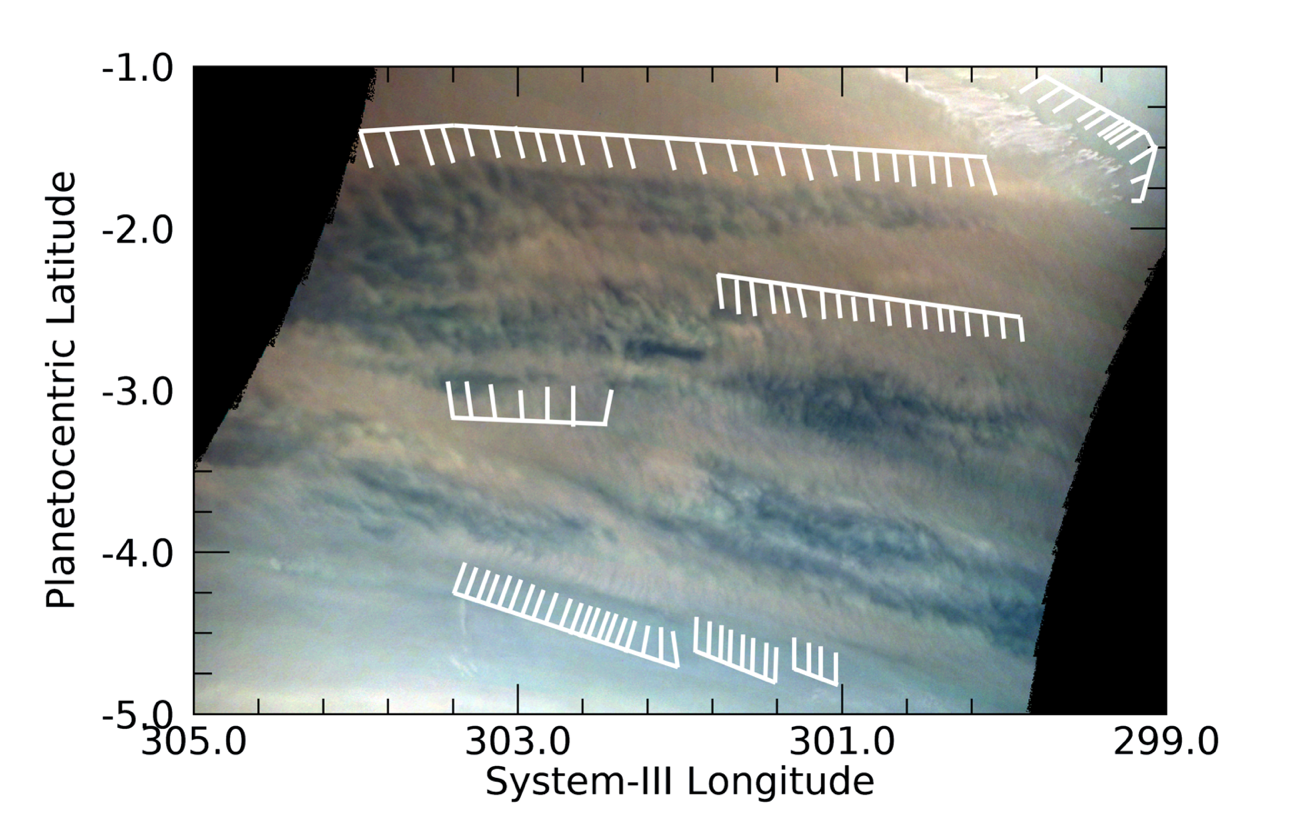


Figure PJ12­_90a. JNCE_2018091_12C00090_V01. Several packets of waves are detectable at all latitudes in this image; we indicate several of them by the grids of white lines. This is the first PJ at which images of the Equatorial Zone were returned at maximum quality specifically for the purpose of examining these waves.


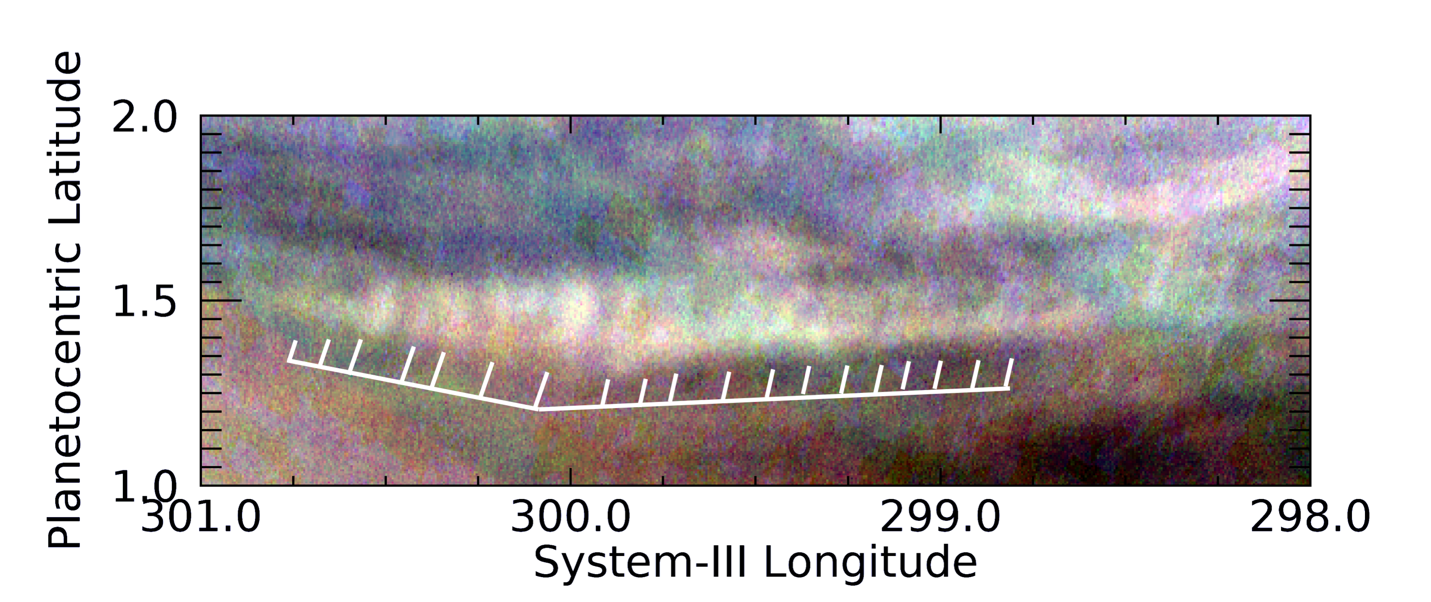
 Figure PJ12_90b. JNCE_2018091_12C00090_V01. A pattern of waves associated with a relatively brighter cloud pattern in the Equatorial Zone is evident above the noise of this image.


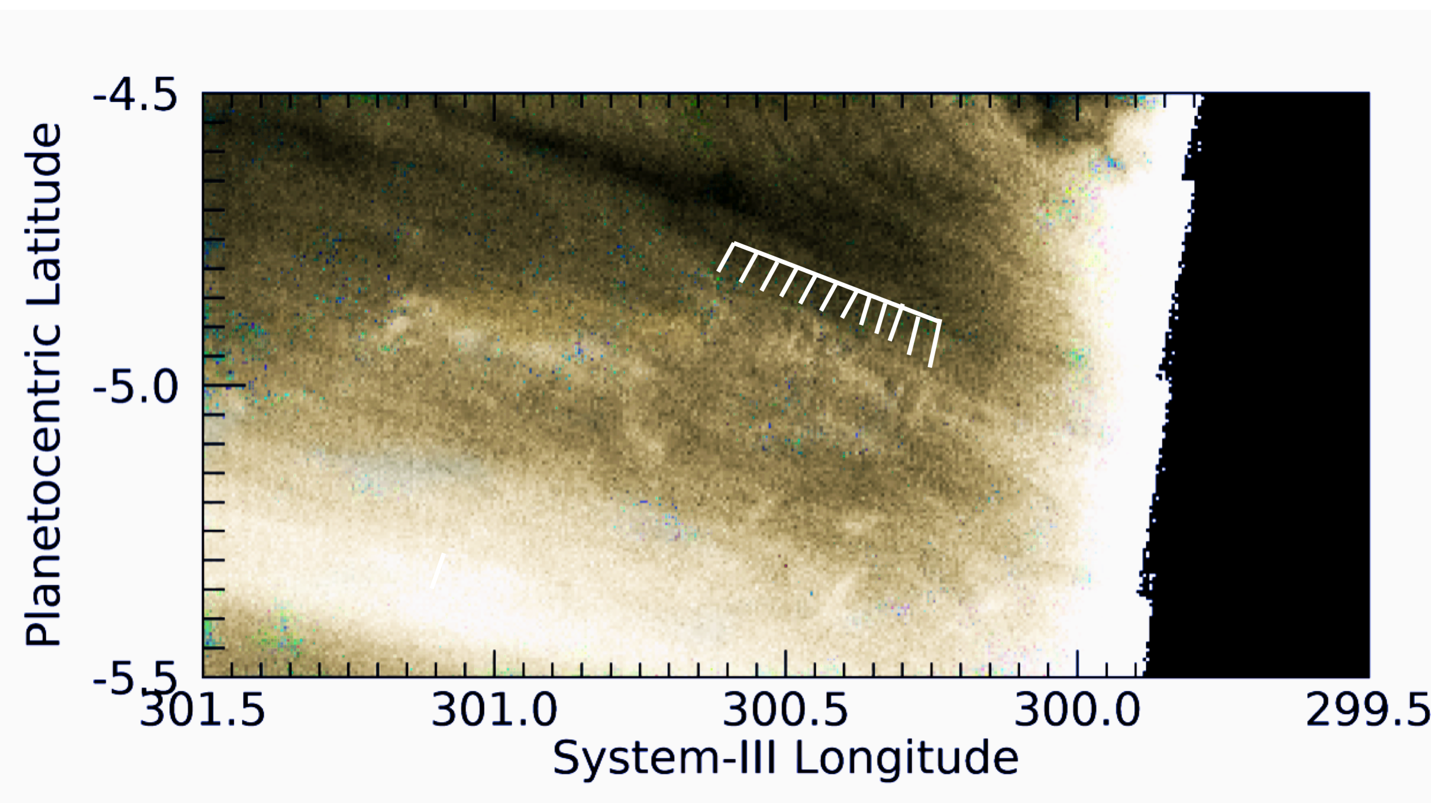


Figure PJ12_90c. JNCE_2018091_12C00090_V01. These very subtle wave-fronts in the Equatorial Zone are detectable above the noise in all three filters. Color-adjustment processing has been done to remove striped artefacts from the image.


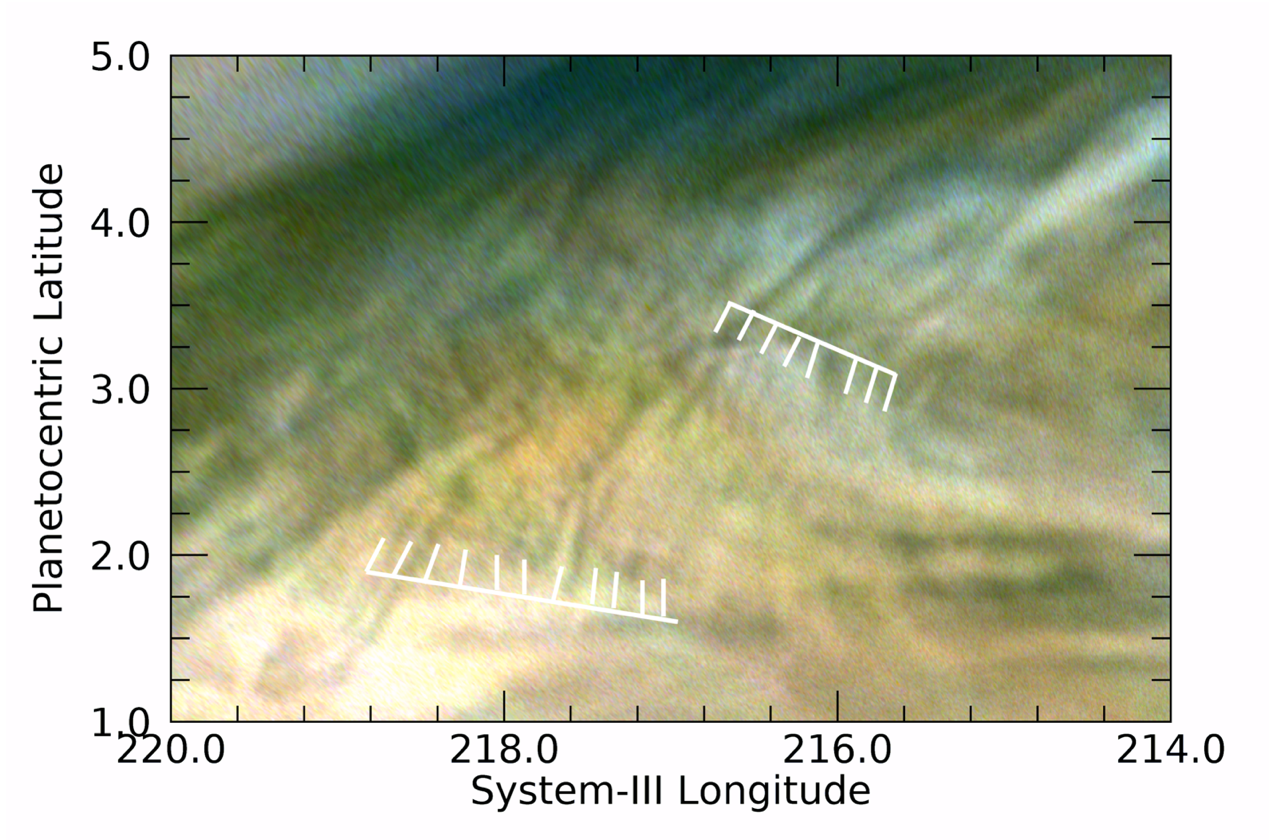


Figure PJ13_35a. JNCE_2018144_13C00035_V01. A series of elongated waves in the Equatorial Zone is detected in this image.


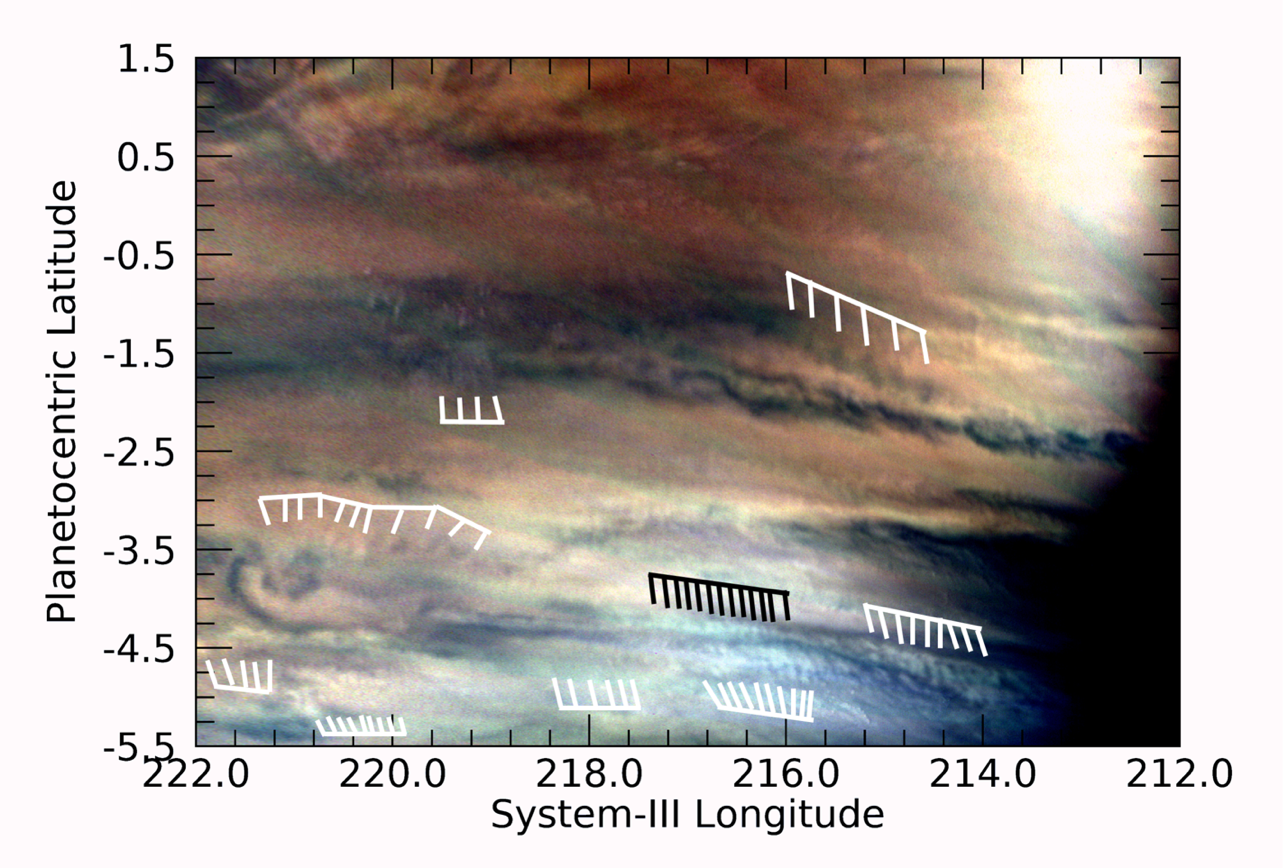


Figure PJ13_35b. JNCE_2018144_13C00035_V01. Many wave packets are detectable in and along the southern edges of the Equatorial Zone and into the South Equatorial Belt. Several of these are indicated in this figure.

.


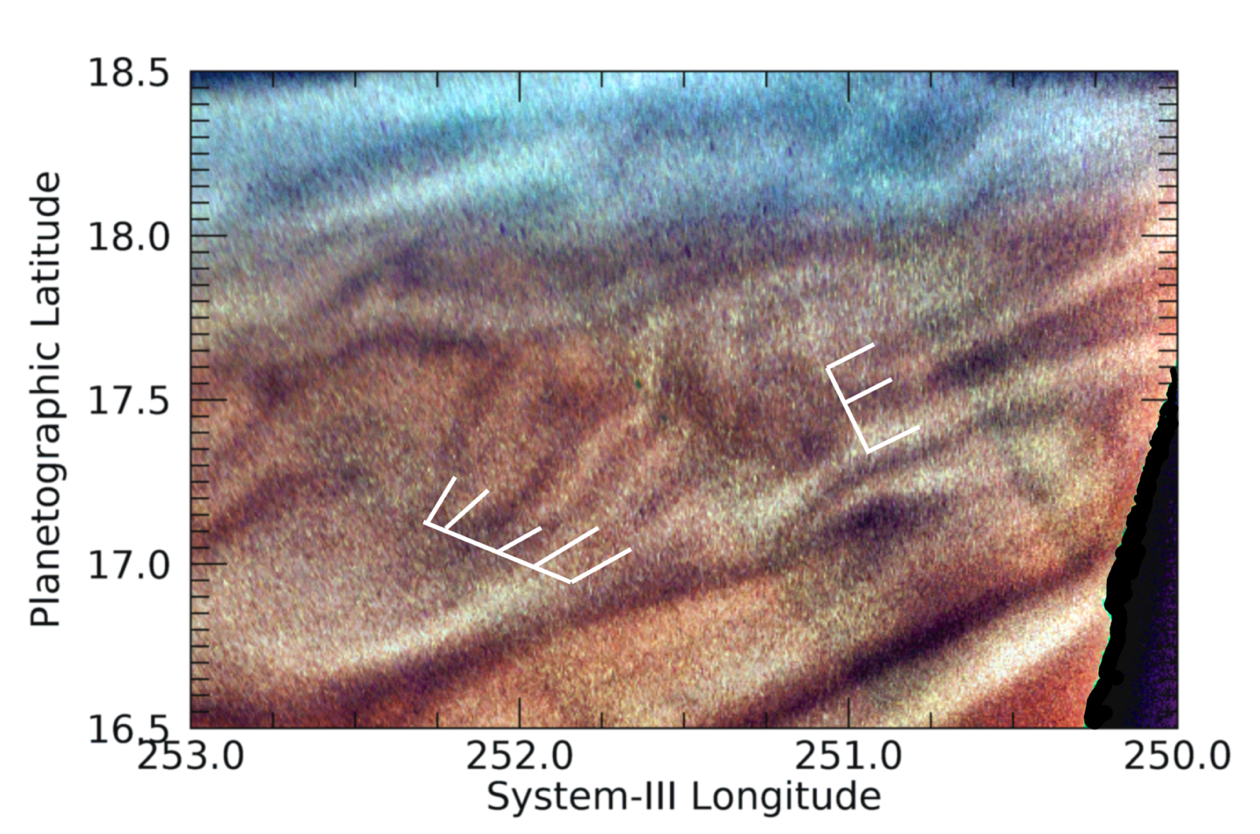

Figure PJ14_25a. JNCE_2018197_14C00025_V01. These dark streaks are in a yellowish strip of the North Tropical Zone, just north of the NEBn retrograde jet where it flows around a barge, a part of which is seen in the bottom-right-hand corner of this figure. The most prominent streaks in the lower part of the figure may be aligned with the jet and mark lines of atmospheric flow.

(Note that we were most successful in minimizing the artificial green stripes in this image by combining the regular map and a high-passed version of the map, decomposing both into three HSL - hue, saturation, lightness - channels and combining the L component of the high-passed map, with the H and S components of the regular map to create a colorized map of high contrast.)


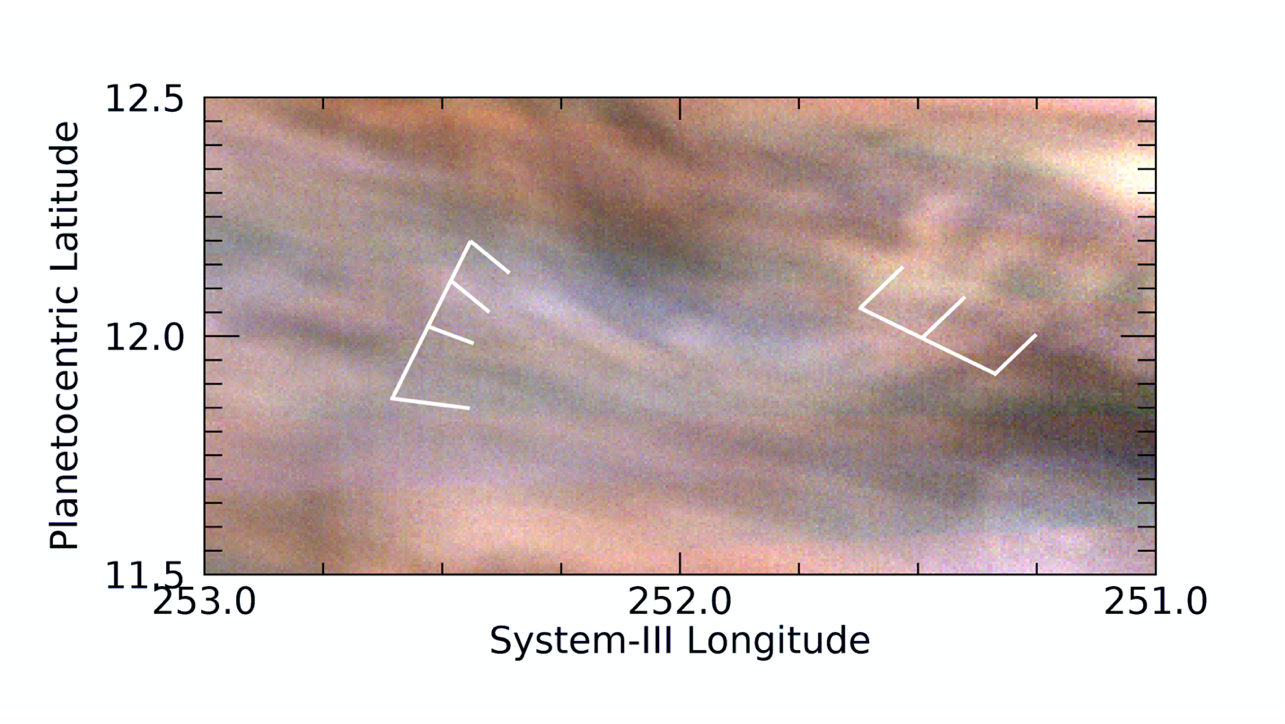


Figure PJ14_25b. JNCE_2018197_14C00025_V01. Repeated patterns are detectable in these otherwise diffuse streaks in the North Equatorial Belt.


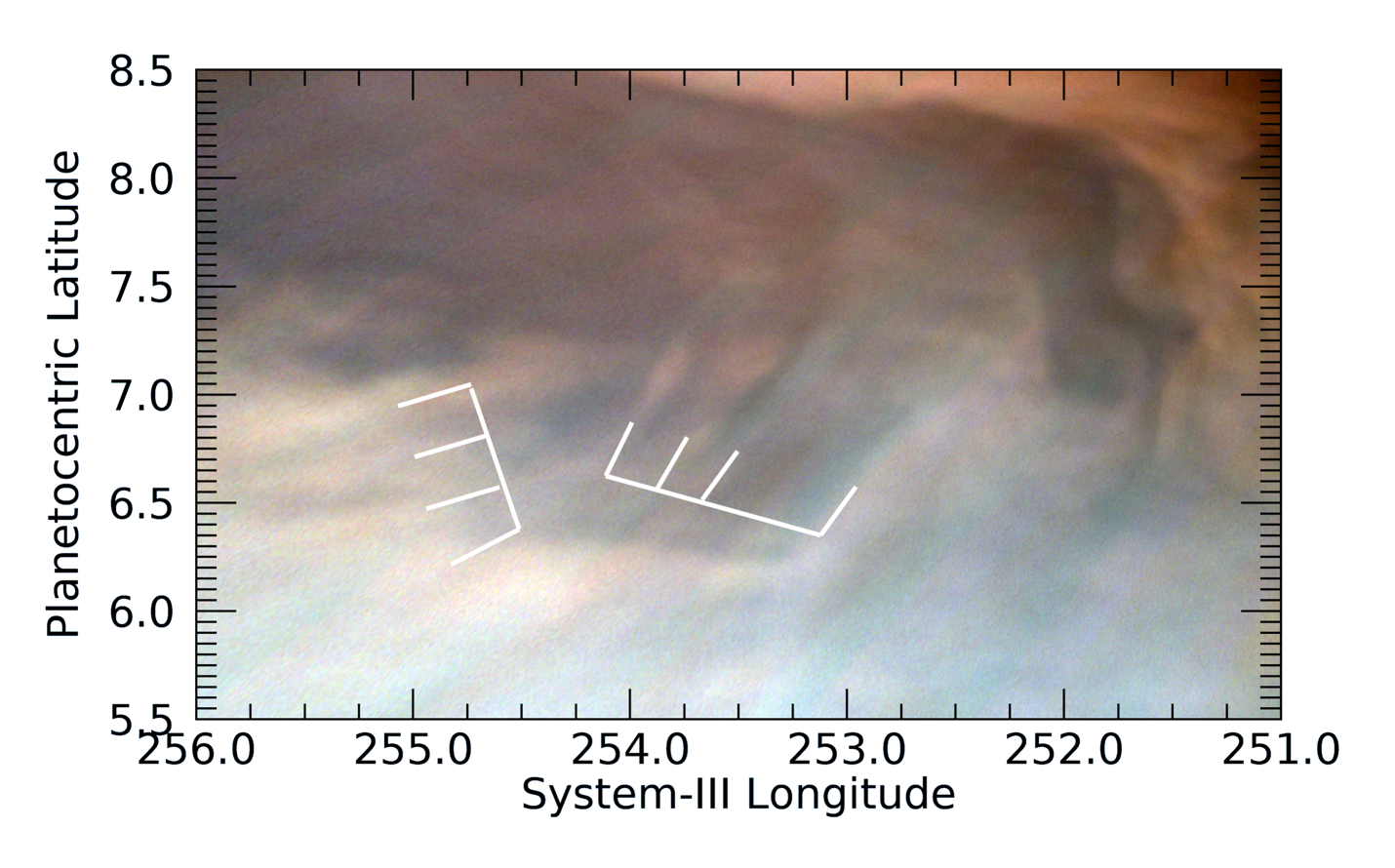


Figure PJ14_25c. JNCE_2018197_14C00025_V01. Similar to the features in Fig. PJ14_25b, Repeated wave-like patterns are detectable here at the boundary between the Equatorial Zone and the North Equatorial Belt. (For both this and the previous figure, the color stretch was softened in order to minize the appearance of artefactual colored stripes.)


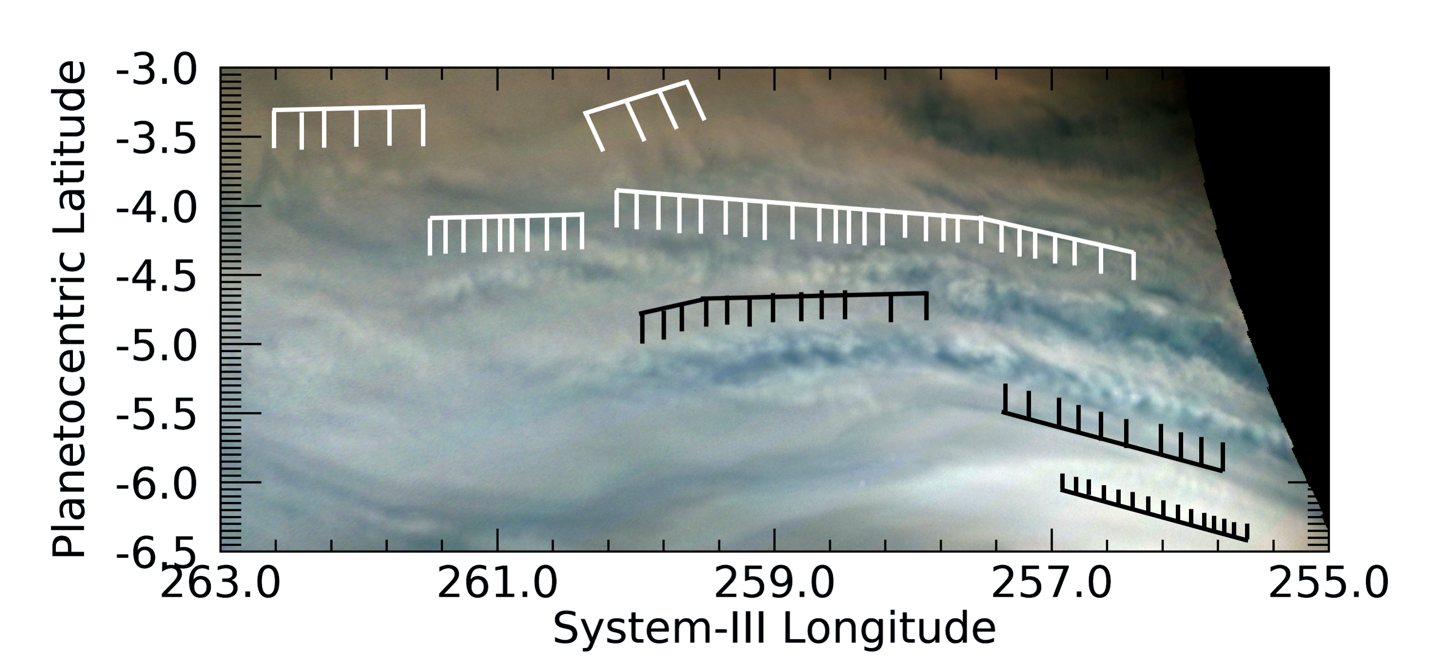


Figure PJ14_28a JNCE_2018197_14C00028_V01. Multiple wave packets are observable in this region of the Equatorial Zone.


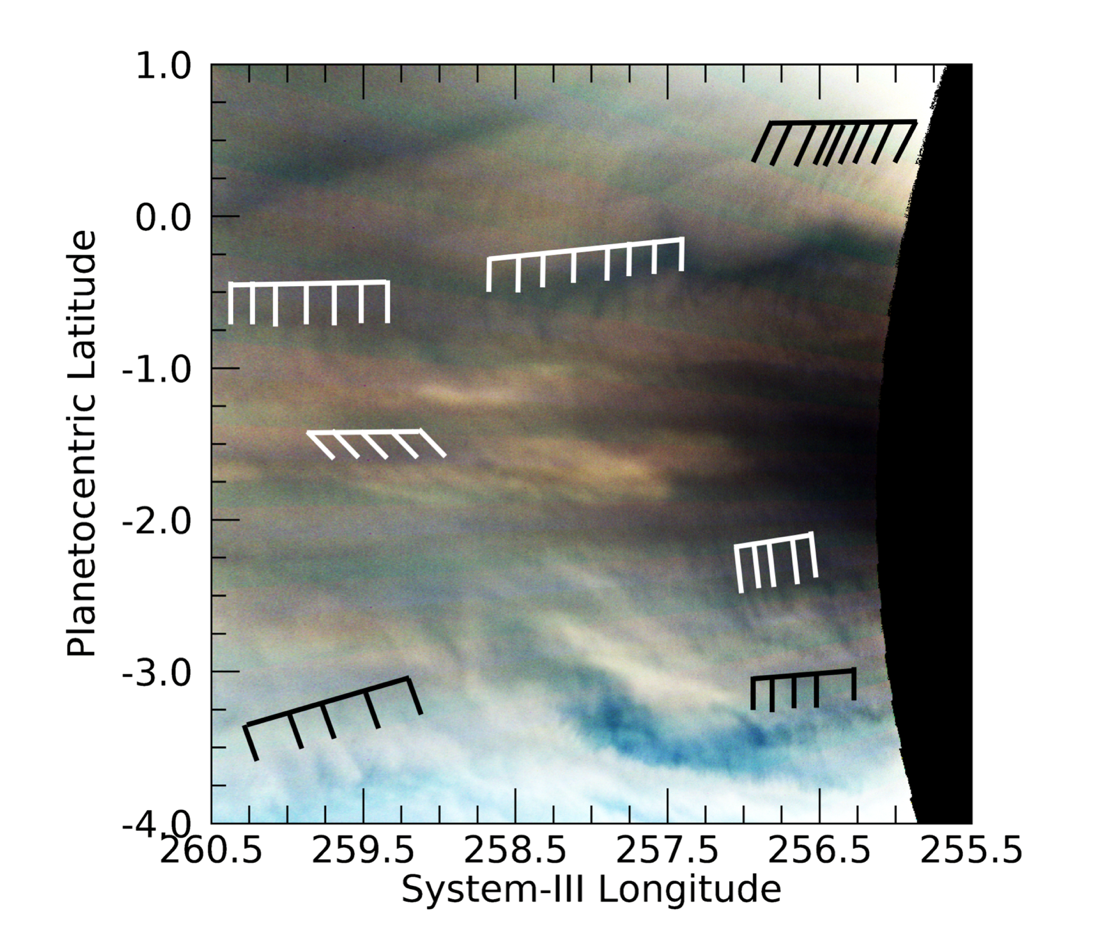


Figure PJ14_28b. JNCE201819714C00028V01. Several wave packets can be detected in this image of the Equatorial Zone, which overlaps slightly with Figure PJ14_28a.


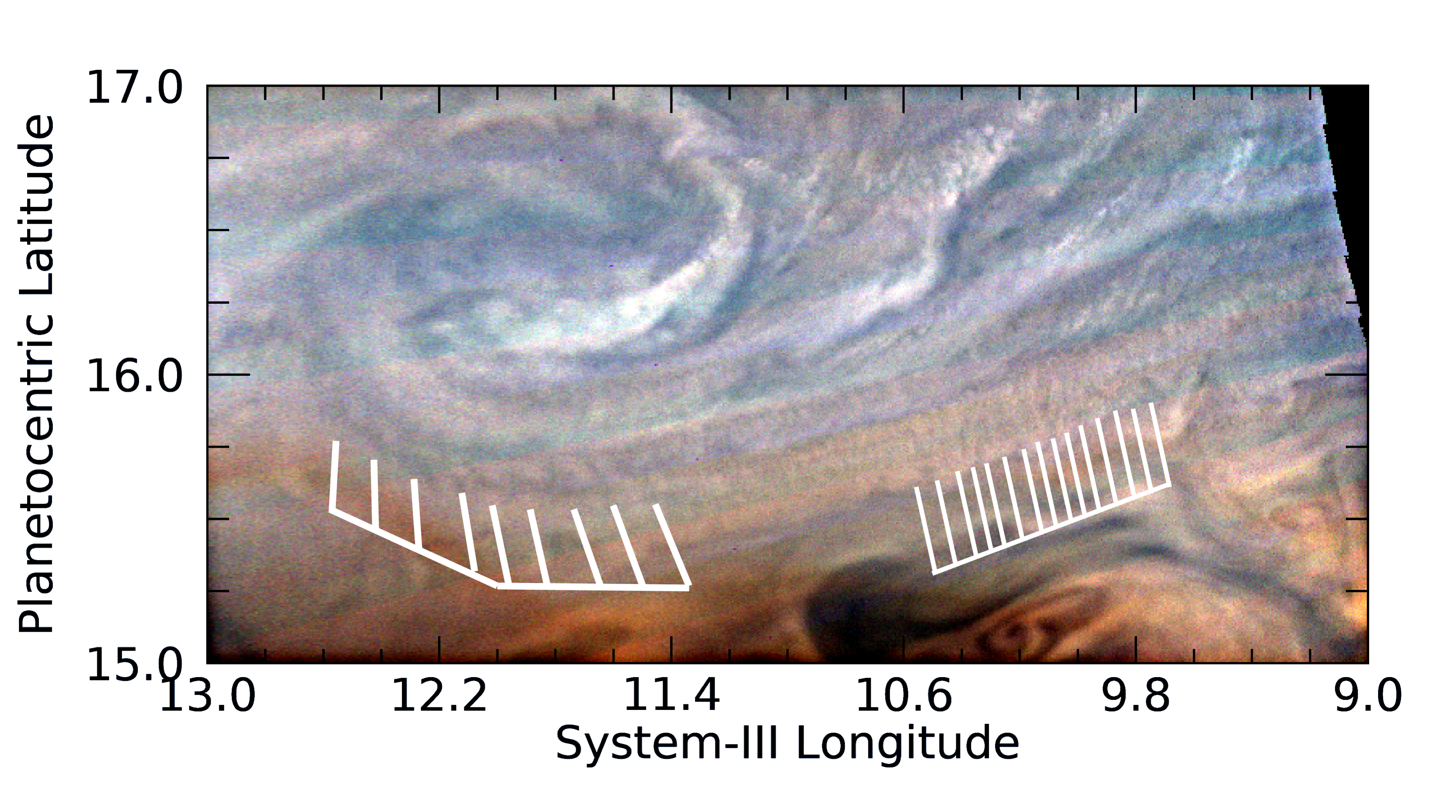


Figure PJ15_26. JNCE_2018250_15C00026_V01. Wave packets with different size wavelengths can be resolved in this region immediately south of a anticyclonic eddy in the northern component of the North Equatorial Belt (NEBn).


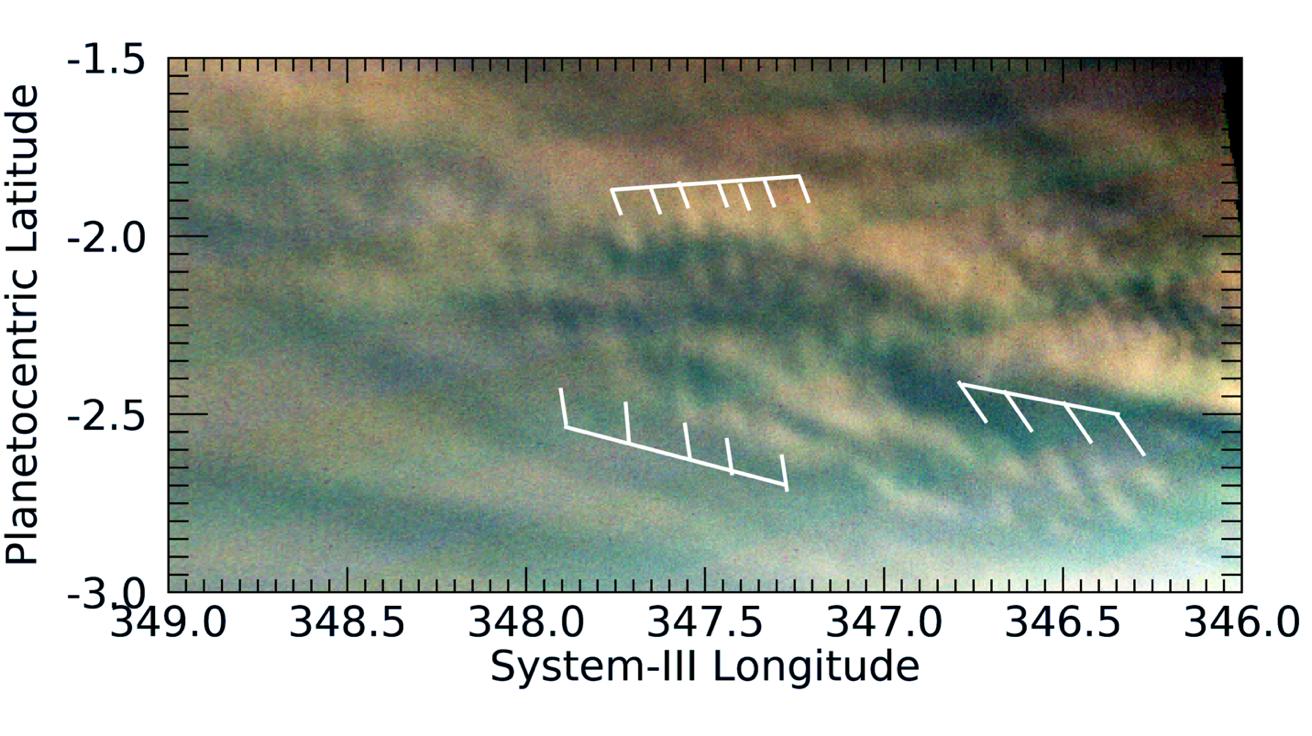
 Figure PJ15_29. JNCE_2018250_15C00029_V01. Several wave packets are present in this image, three of which are indicated by the white grids.


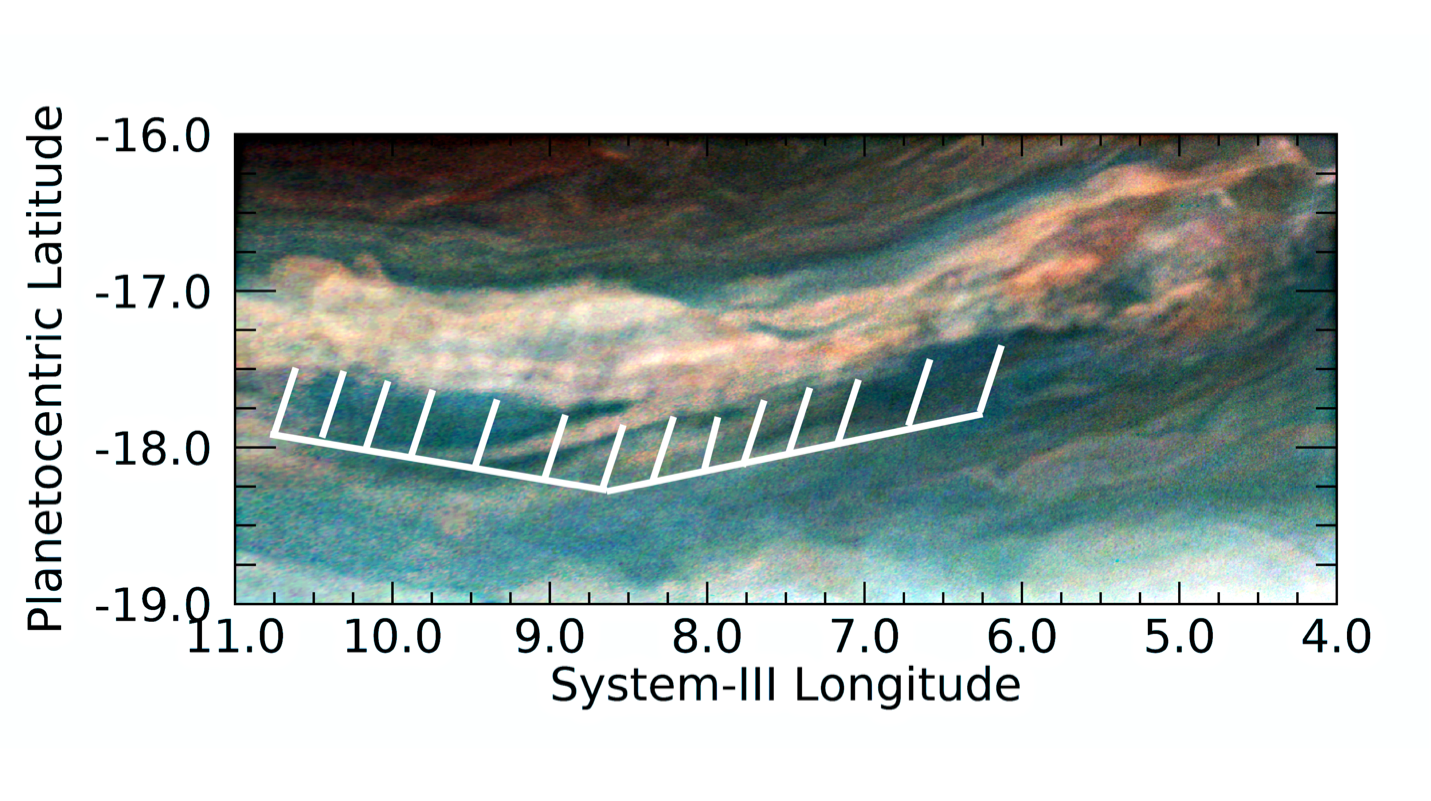


Figure PJ15_31. JNCE_2018250_15C00031_V01. Several waves are seen in this packet that are associated with a white region on the southern edge of a dark cyclonic vortex, known as a “barge” in the South Equatorial Belt. Some unsharp masking has been applied to this image in order to make the faint waves more prominent.


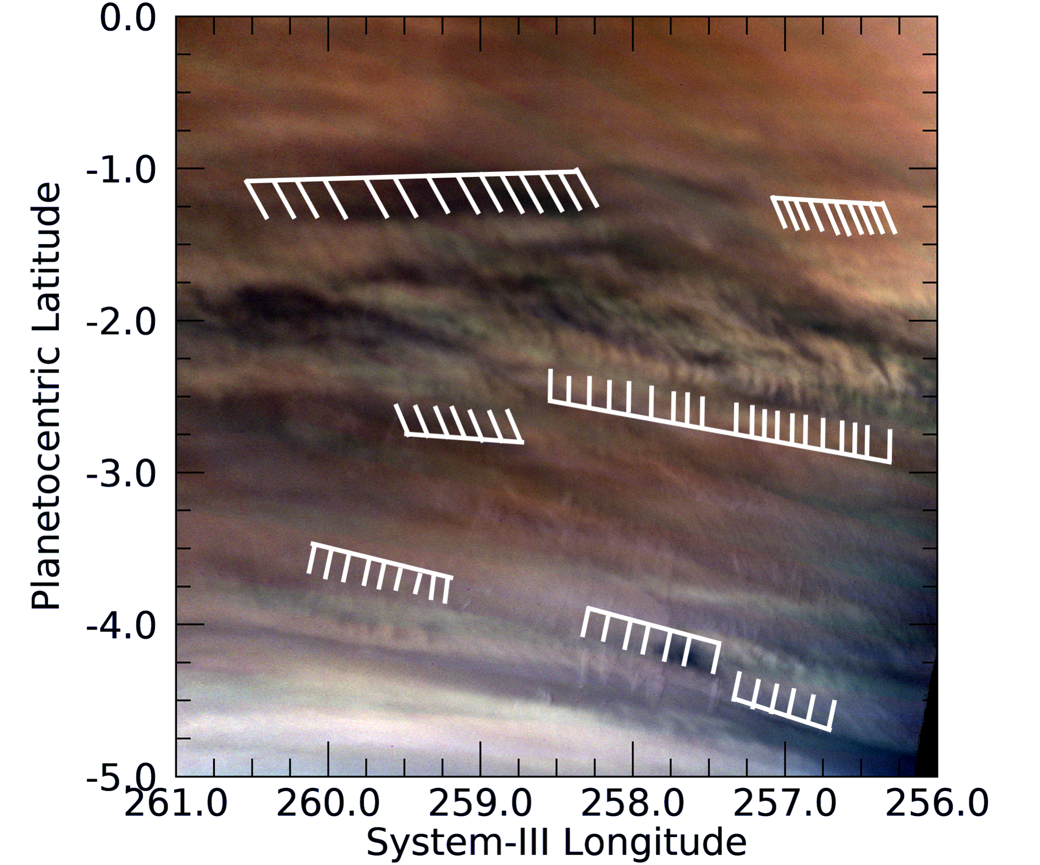


Figure PJ16_20. JNCE_2018302_16C00020_V01. Multiple wave packets are detectable in this image of the Equatorial Zone, several of which are indicated by the white grid lines. Many wavefronts are nearly perpendicular to the direction of the wave packet.


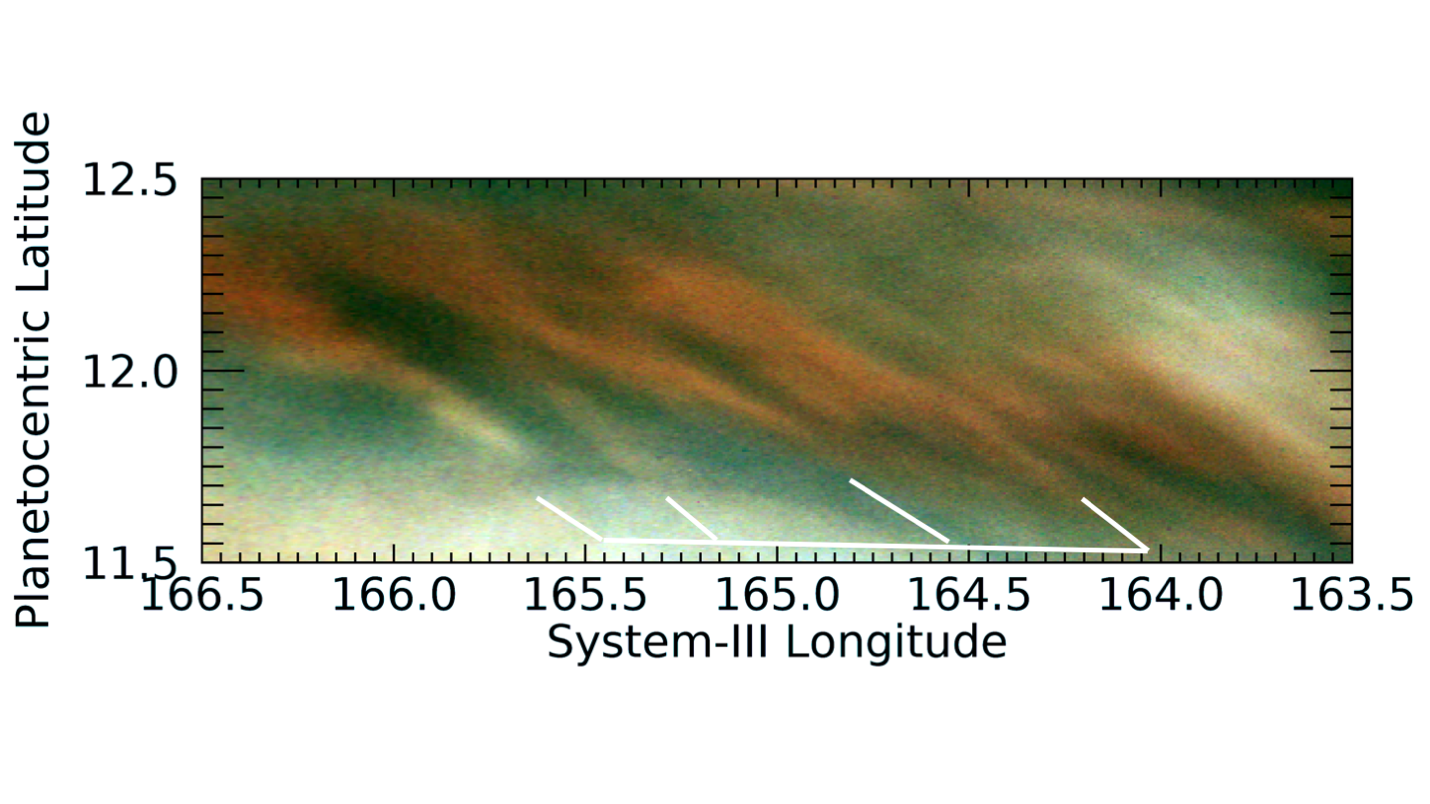
Figure PJ17_24. JNCE_2018355_17C00024_V01. Some streak-like features are detected in the middle of the North Equatorial Belt. This region is dominated by cyclonic motion, so the organization of this feature parallels the zonal-wind gradient. The exaggerated large-scale diffuse color contrast across the image may represent diffuse bands of white or reddish haze overlying the main cloud deck.


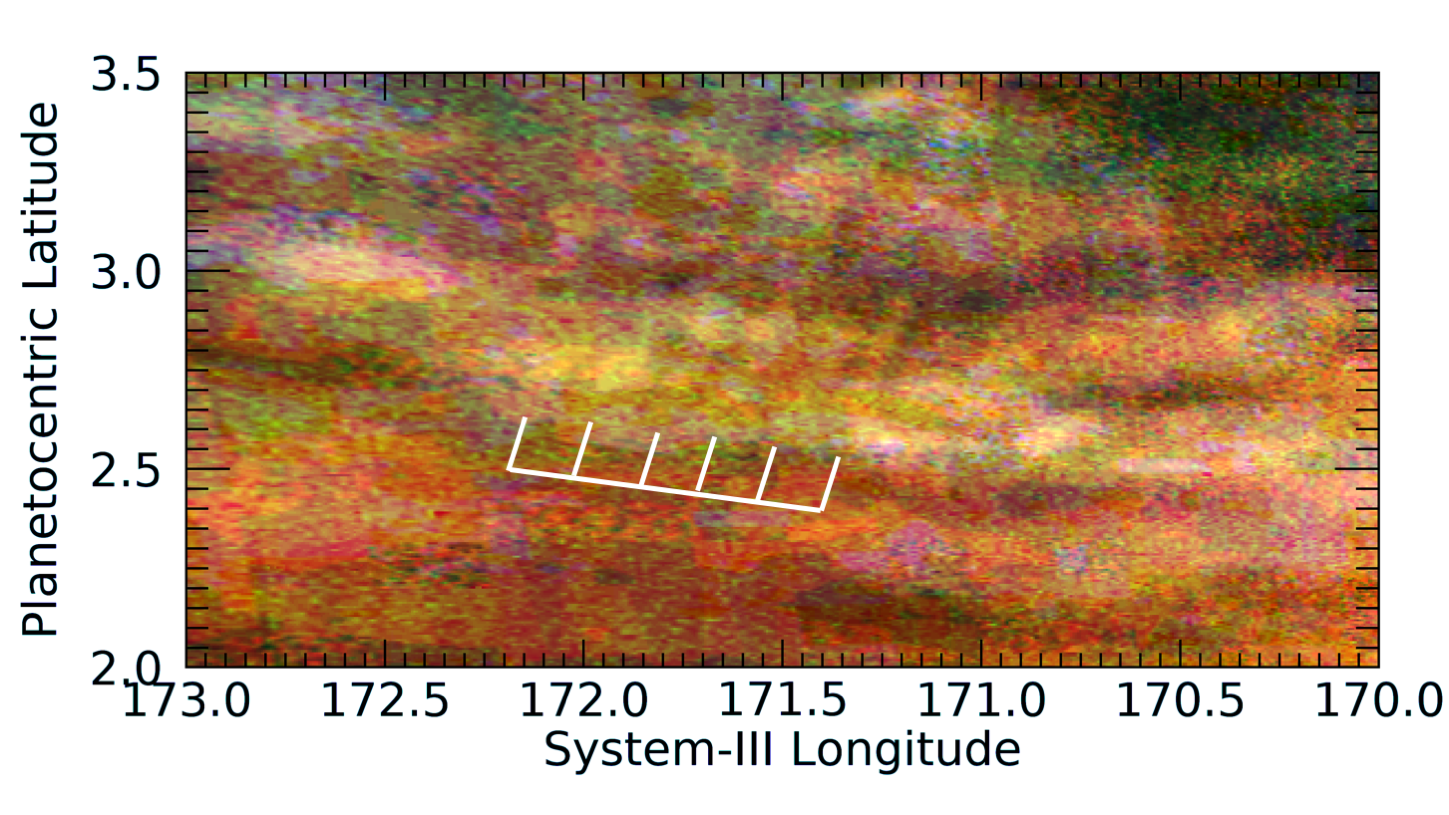


Figure PJ17_26a. JNCE_2018355_17C00026_V01. A short, relatively faint wave packet is detectable in this image of the Equatorial Zone.


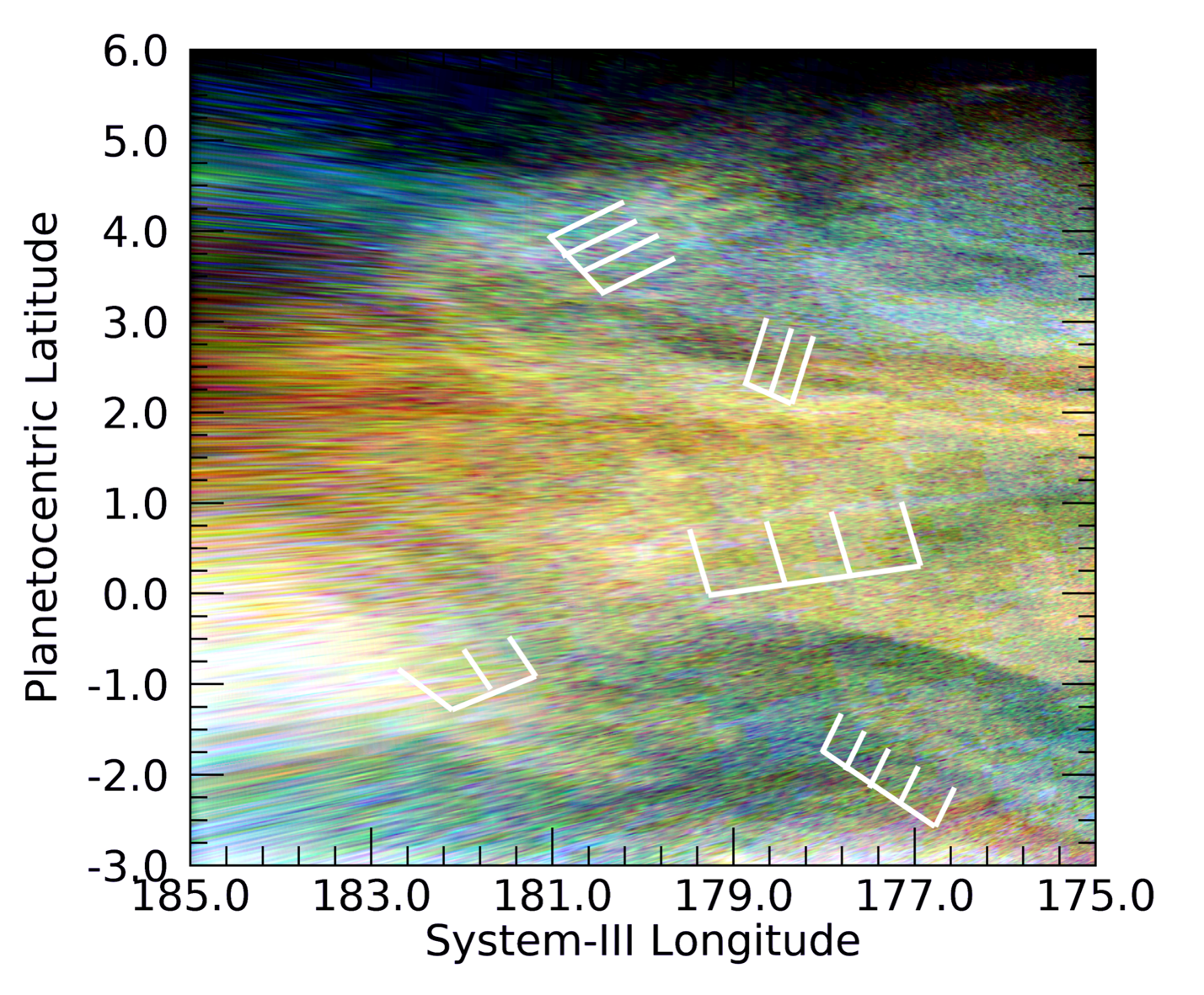


Figure PJ17_26b. JNCE_2018355_17C00026_V01. Several wave packets, some overlapping, are detectable in this image of the Equatorial Zone, with several indicated by the white grids.


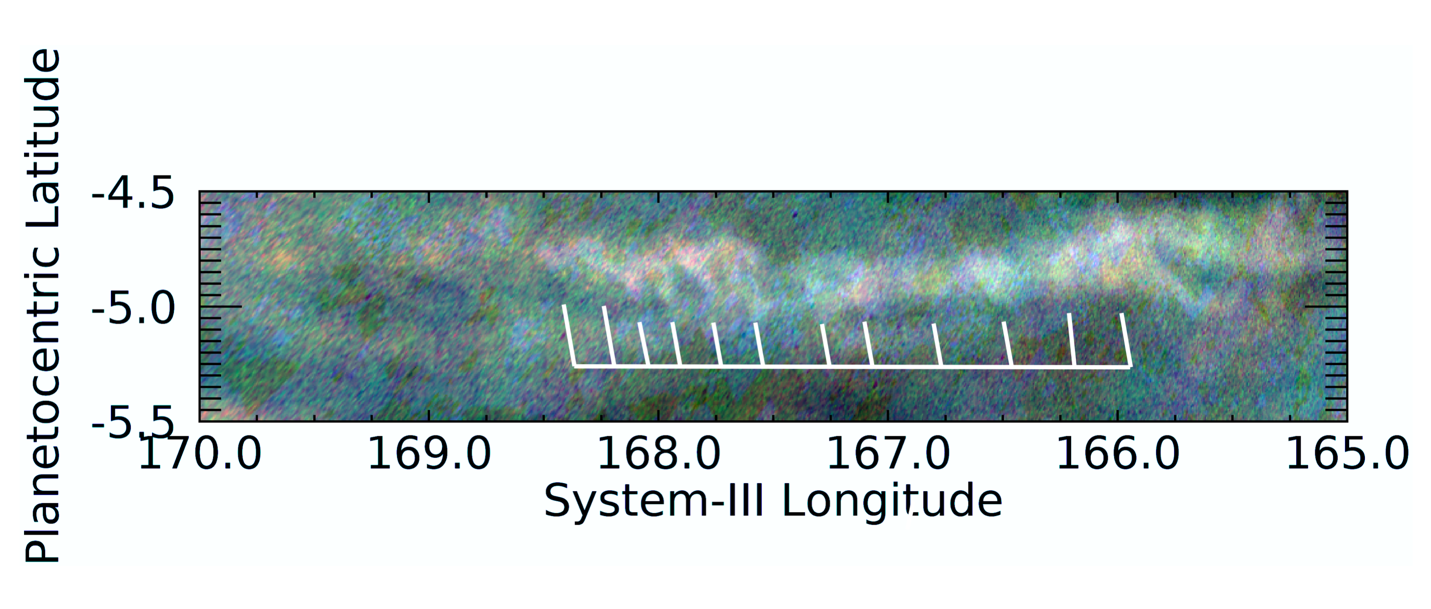


Figure PJ17_26c. JNCE_2018355_17C00026_V06. A short sequence of discrete features is evident in this image of a region within the Equatorial Zone.


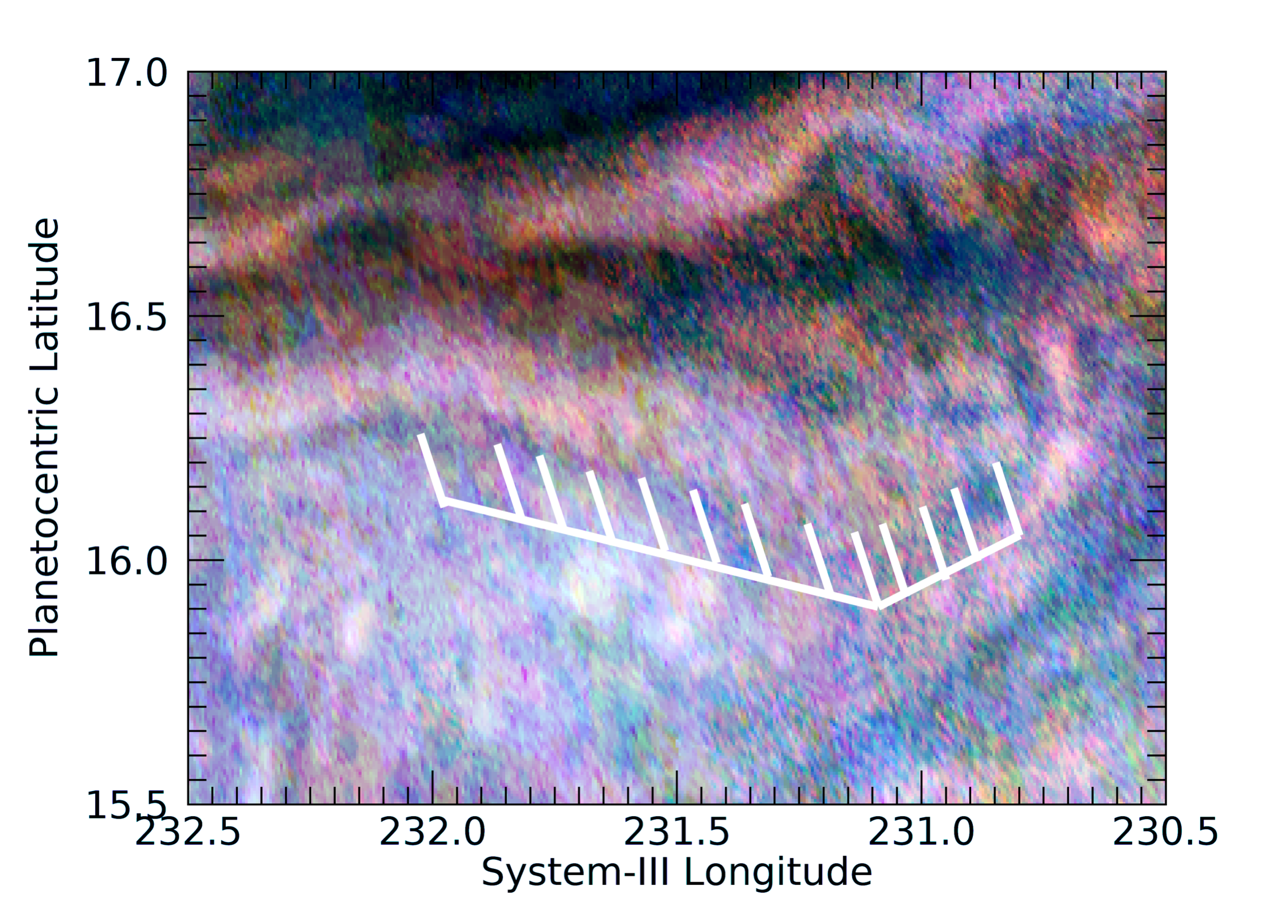


Figure PJ18_34. JNCE_2019043_18C00034_V01. Very faint waves are detectable above the noise in this image. (Note that the extreme stretching needed to display the waves is responsible for the unusual color scheme of this figure.)


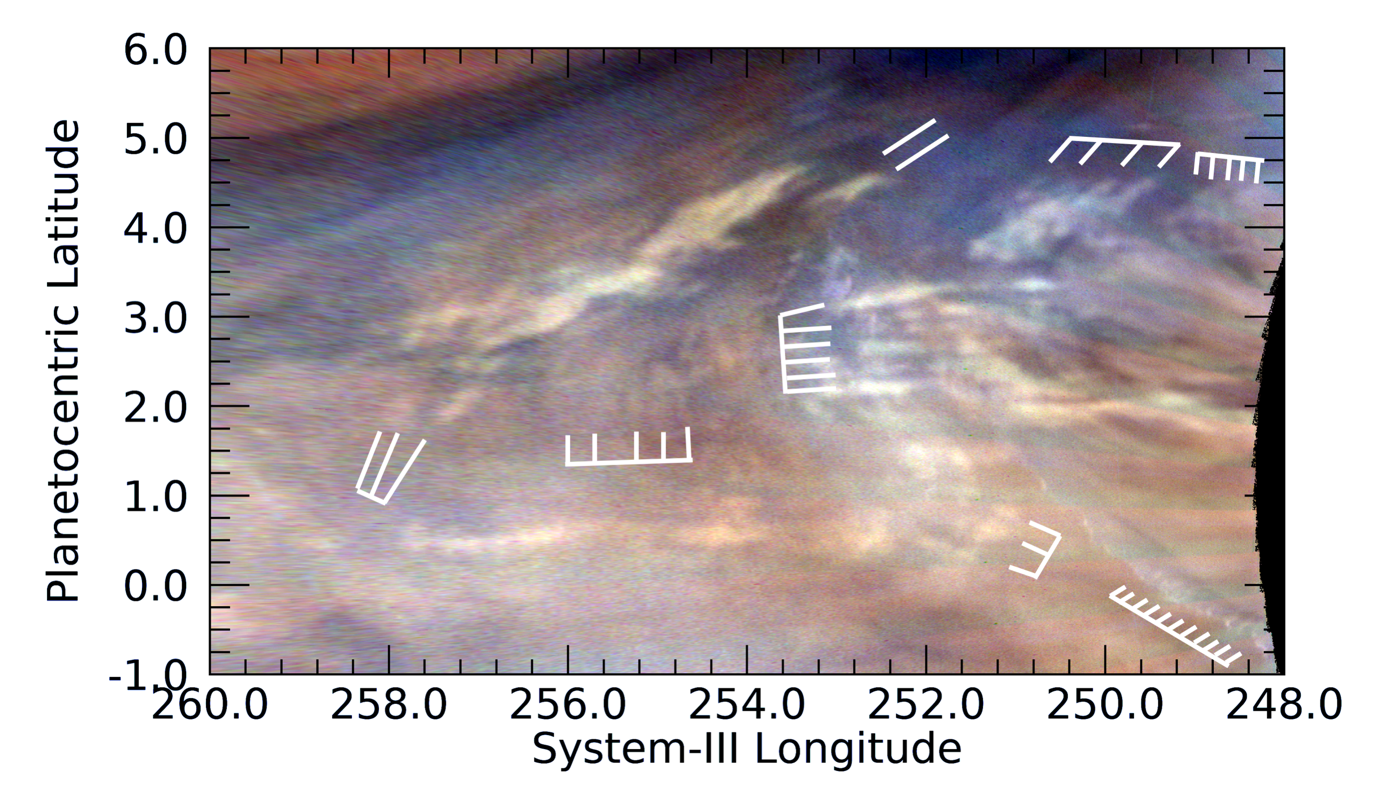


Figure PJ18_38. JNCE_2019043_18C00038_V01. This region in the northern component of the Equatorial Zone is filled with overlapping waves, several of which are indicated by the white grid lines.


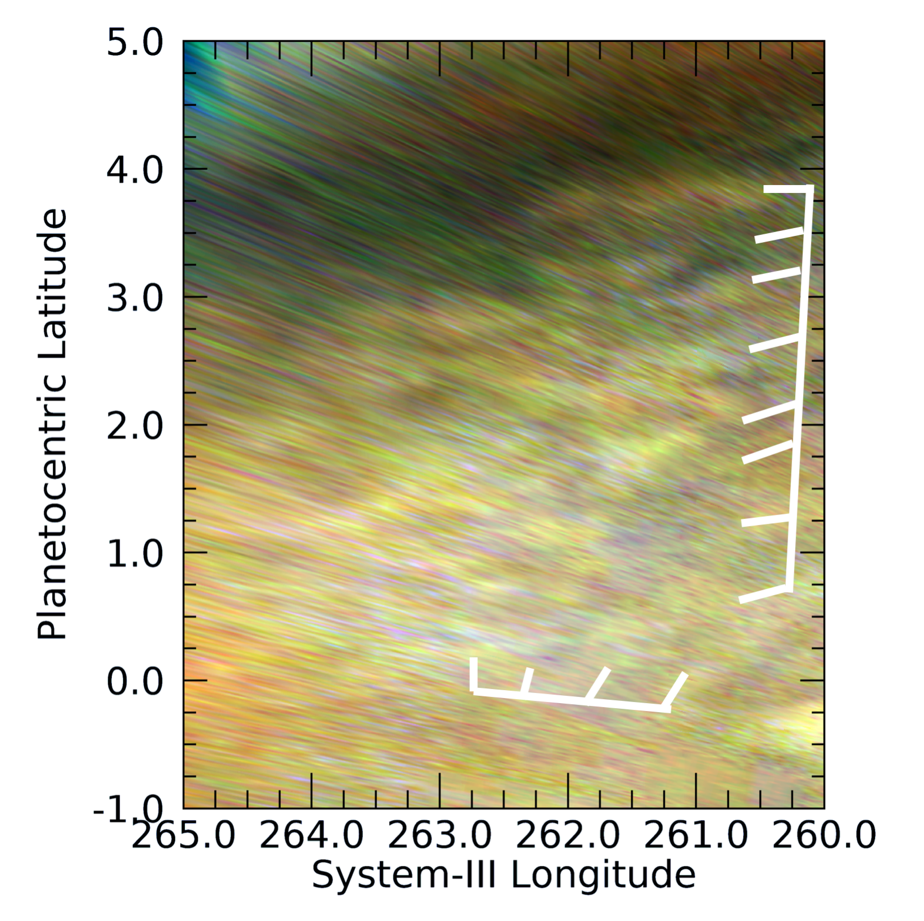


Figure PJ18_39a. JNCE_2019043_18C00039_V01. Several independent wavefronts are detectable in cross directions are detectable in this image of the Equatorial Zone, two of which are indicated by the white grids. This region is immediately to the west of the region shown in Figure PJ18_39b.


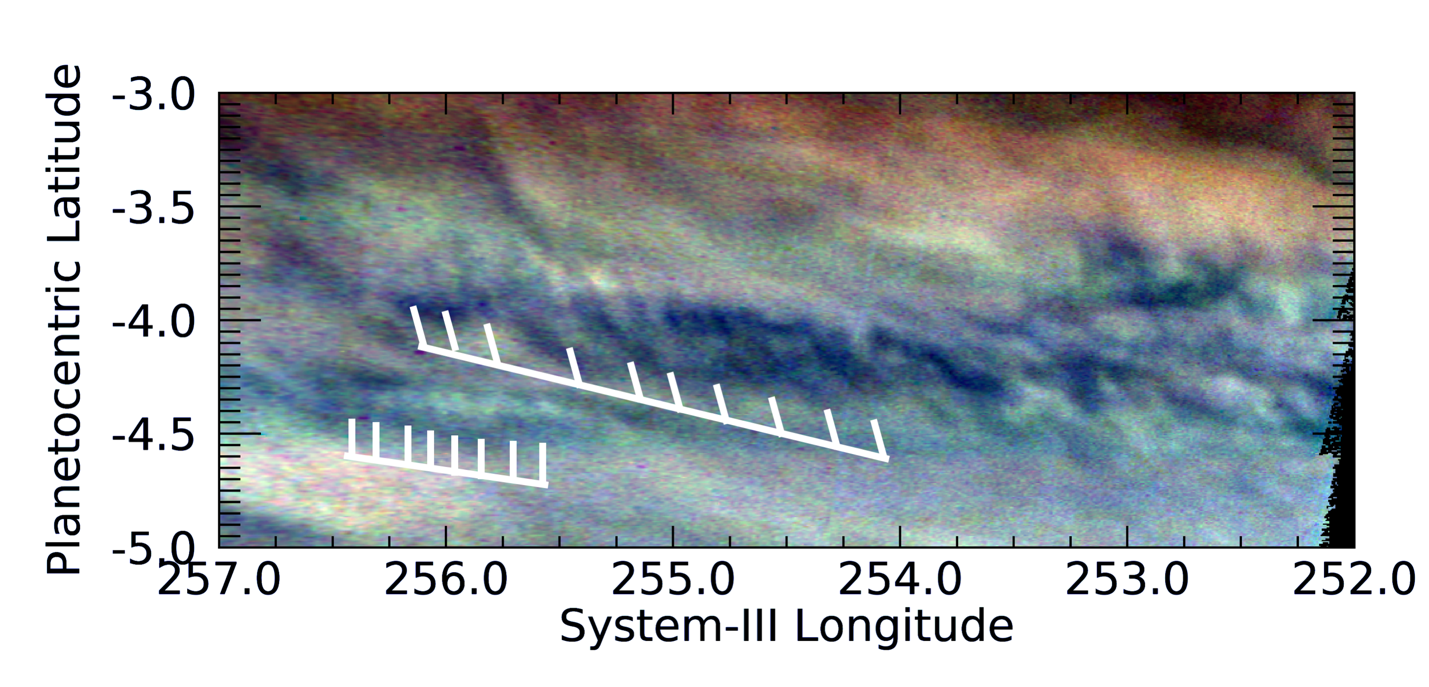


Figure PJ18_39b. JNCE_2019043_18C00039_V01. Very faint waves are detectable in this image of the southern component of the Equatorial Zone.


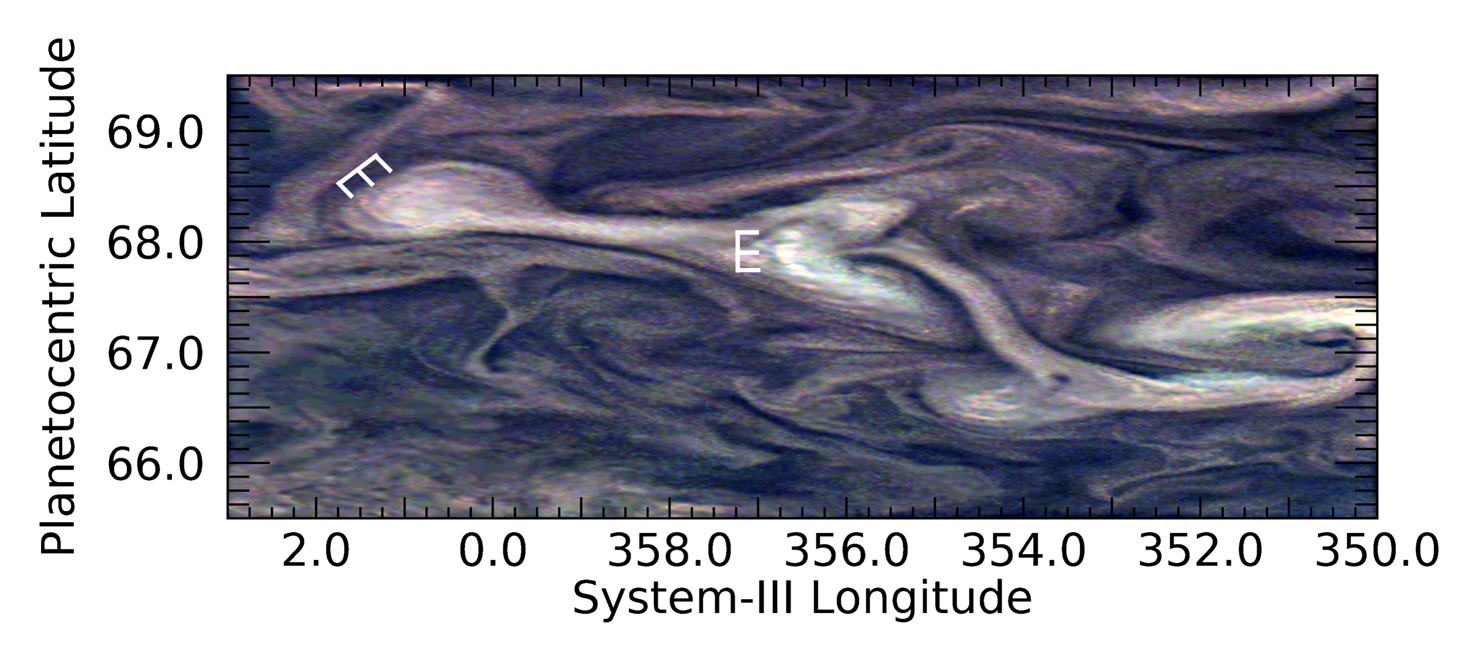


Figure PJ20_24 JNCE_2019043_20C00024_V01. A regular sequence of three bright cloud features can be seen within a branch of a relatively bright feature with two distorted lobes to the east and west of the features indicated by the white grid. The white grid in the upper left indicates another set of three somewhat offset whitish discrete clouds, just within the resolution of JunoCam.

This area is well into the north polar region that is not dominated by zonal winds. It is the furthest north that JunoCam has detected anything resembling a repeatable feature. Moderate unsharp masking has been performed in order to make the subtle features indicated in the upper left more recognizable.


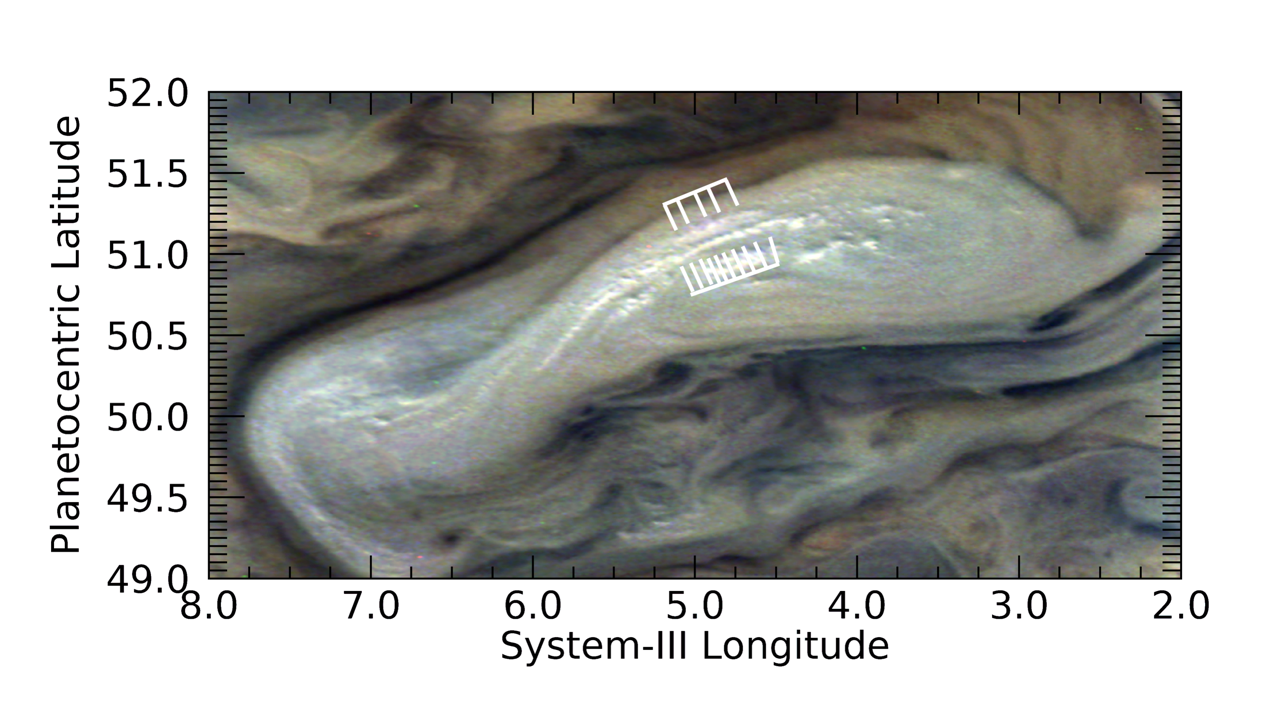


Figure PJ20_26a. JNCE_2019043_20C00026_V01. Regular sequences of variable cloud features can be detected along these cloud banks that are just within the resolution of JunoCam inside this anticyclonic vortex. This region and the one shown in Fig. PJ20_26b are immediately south of the prograde jet at a planetocentric latitude of 53°N.


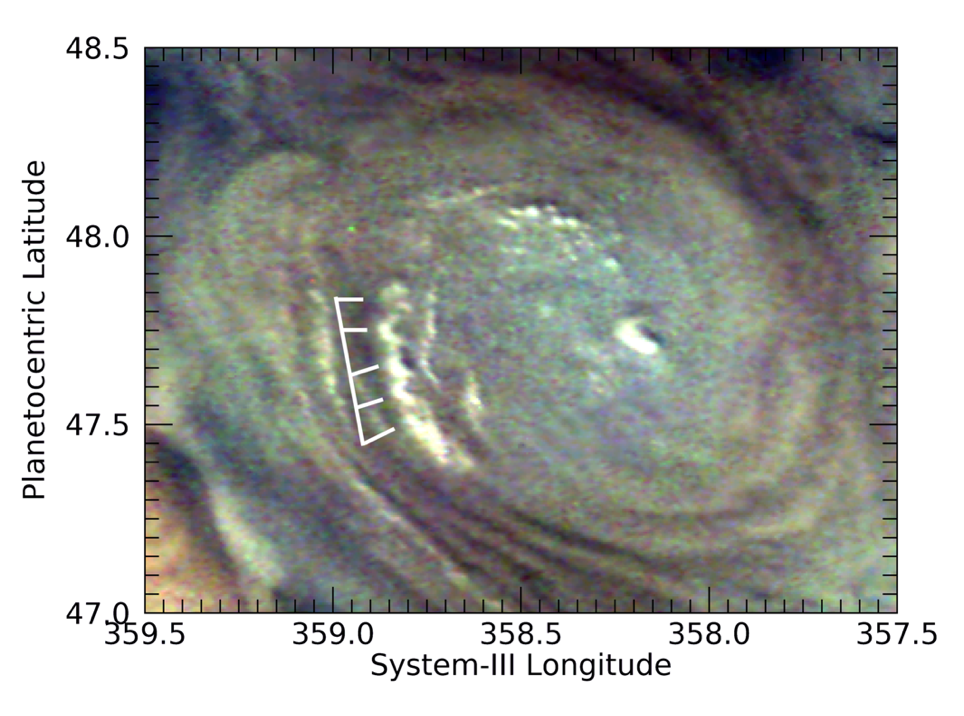


Figure PJ20_26b. JNCE_2019043_20C00026_V01. As in Figure PJ20_1, we detect a regular sequence of variable, somewhat arcuate, cloud features along a curved cloud bank in the western side of this anticyclonic vortex. Banks of clouds such as these and those in Fig. PJ20_26a have been seen in previous perijoves, starting prominently in PJ6, but the regions shown in Figs. PJ04_103b, PJ12_86c, and PJ20_26a and this figure are the only ones in which we have been able to resolve individual cloud components. Thus, they are the only ones in which we have been able to classify them as being a wave-like phenomenon.


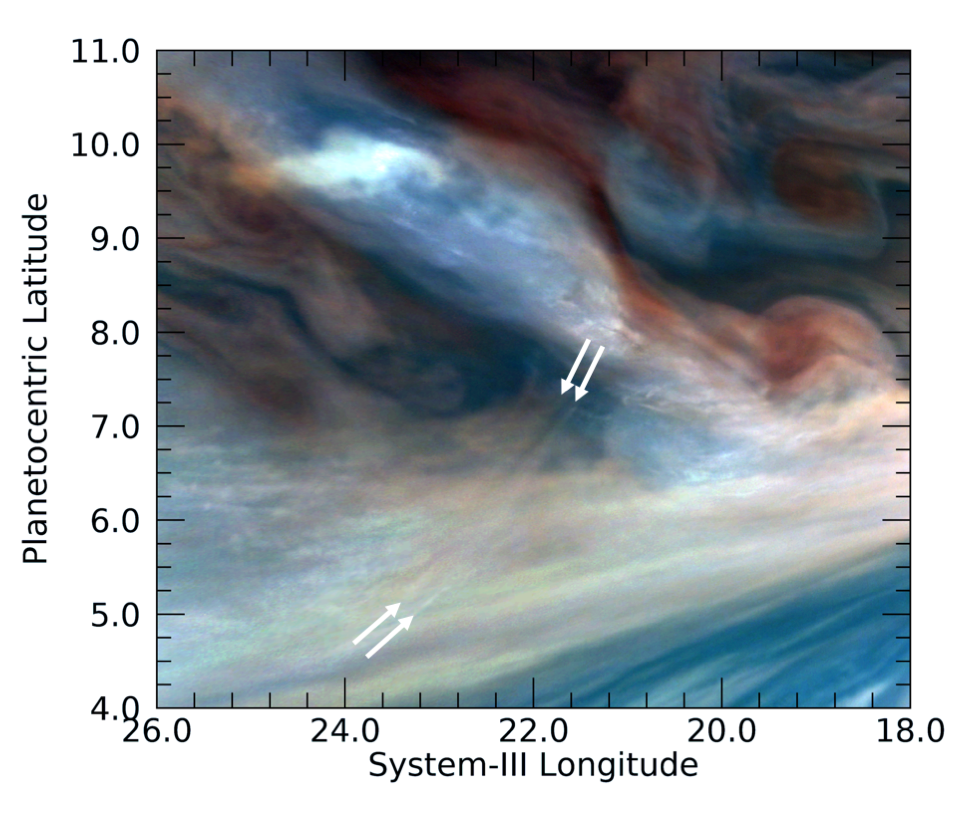


Figure PJ20_33. JNCE_2019043_20C00033_V01. Arrows denote a pair of bright curvilinear waves in the northern component of the Equatorial Zone. This image has also been unsharp-masked, as well as color-stretched, in order to make this feature more prominent.


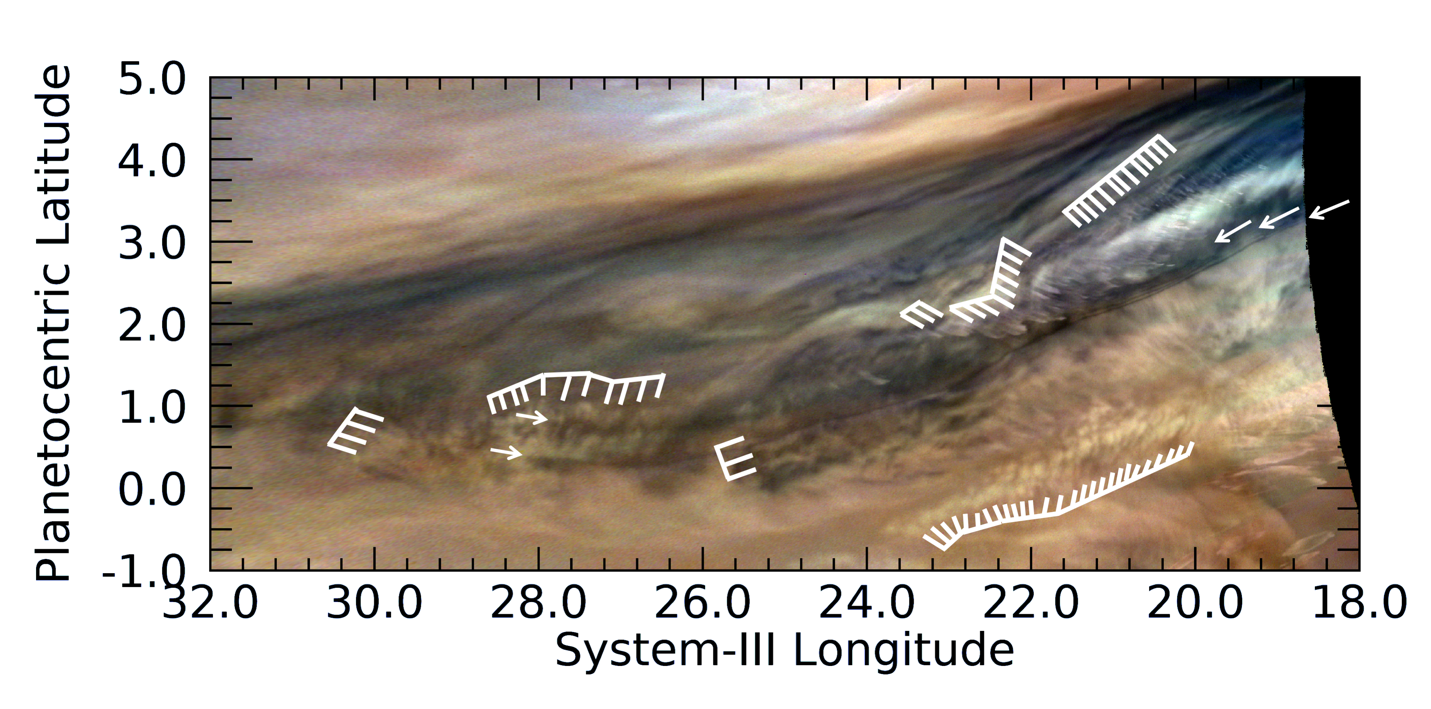


Figure PJ20_34a. JNCE_2019149_20C00034_V01. A wide variety of waves are detectable at this southern extension of a 5-µm hot spot “festoon”, some of which are indicated by white gridlines. This is just south of (and slightly overlapping with) the area in Fig. PJ20_33. Three parallel lines, two of whose origins can be seen on their eastern end, are denoted by left-pointing arrows. Two can be seen out to the point denoted by the right-pointing arrow. (A version of this figure, indicating only the long, dark waves is shown in the Figure 7 of the main article. It also includes the area shown in Fig. PJ20_33.)


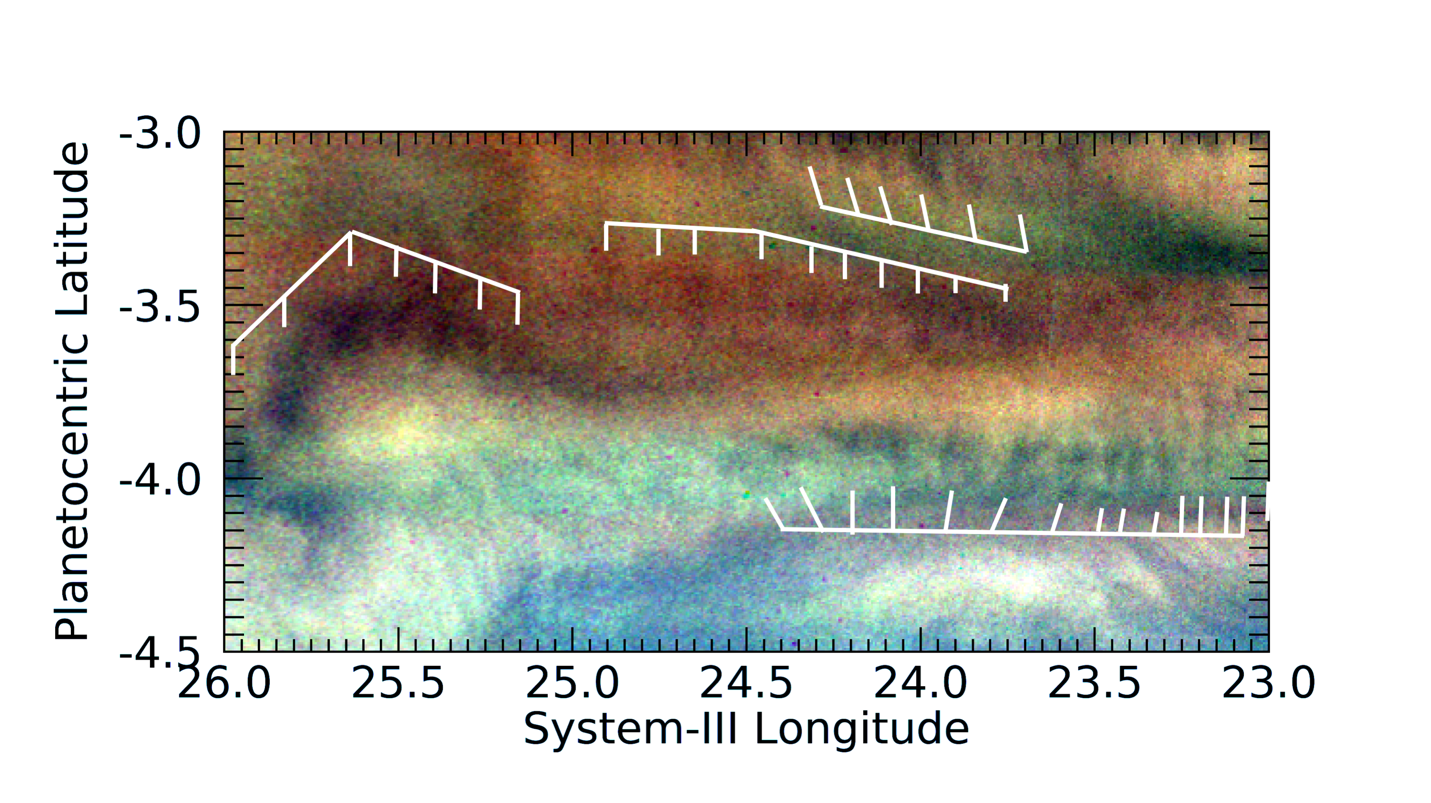


Figure PJ20_34b. JNCE_2019149_20C00034_V01. Long wave packets, some straight and some curved, and short wavefronts in the southern component of the Equatorial Zone. These are barely resolved above the noise.


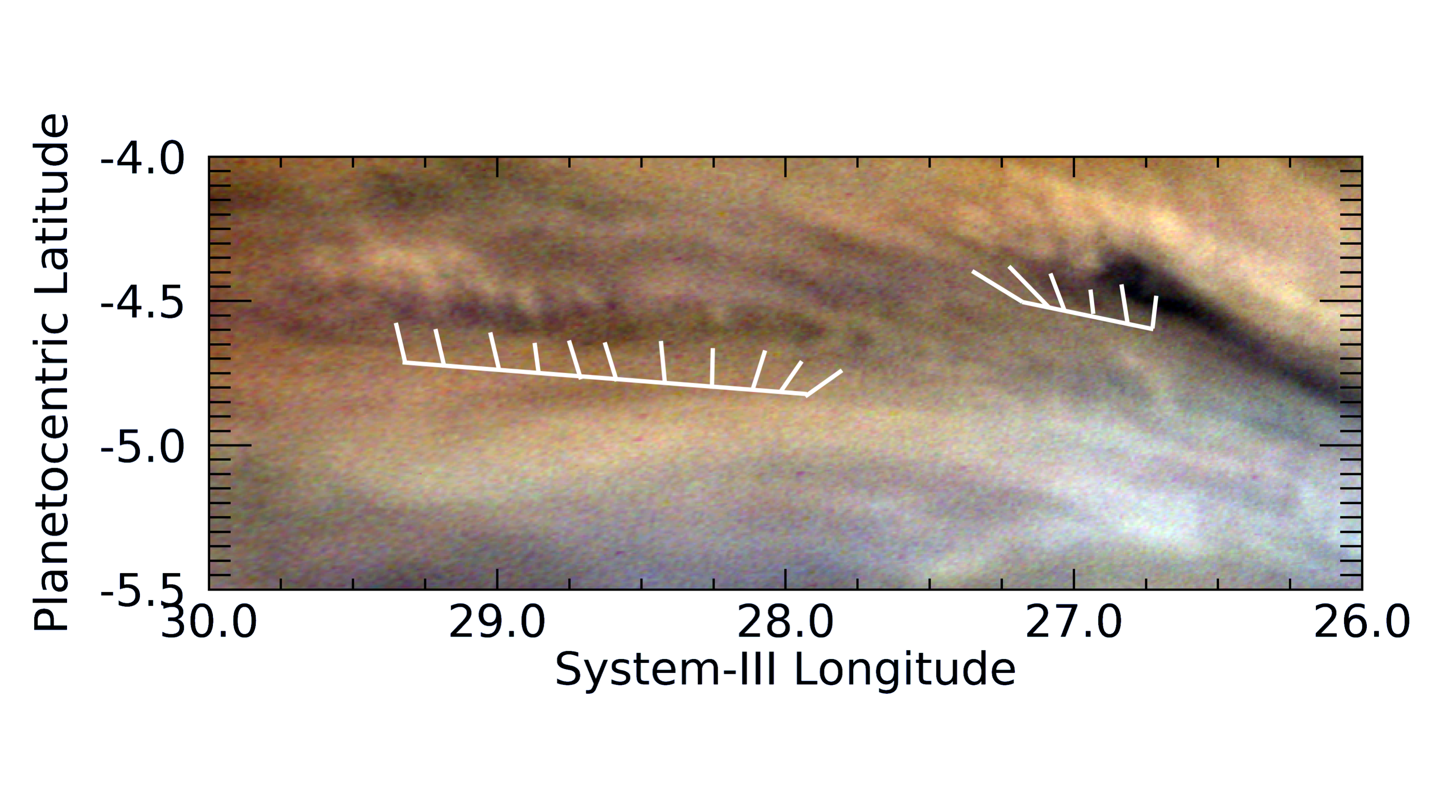


Figure PJ20_34c. JNCE_2019149_20C00034_V01. Long wave packets and short wave fronts just to the west of the area in Fig. PJ20_34b in the southern component of the Equatorial Zone.

.


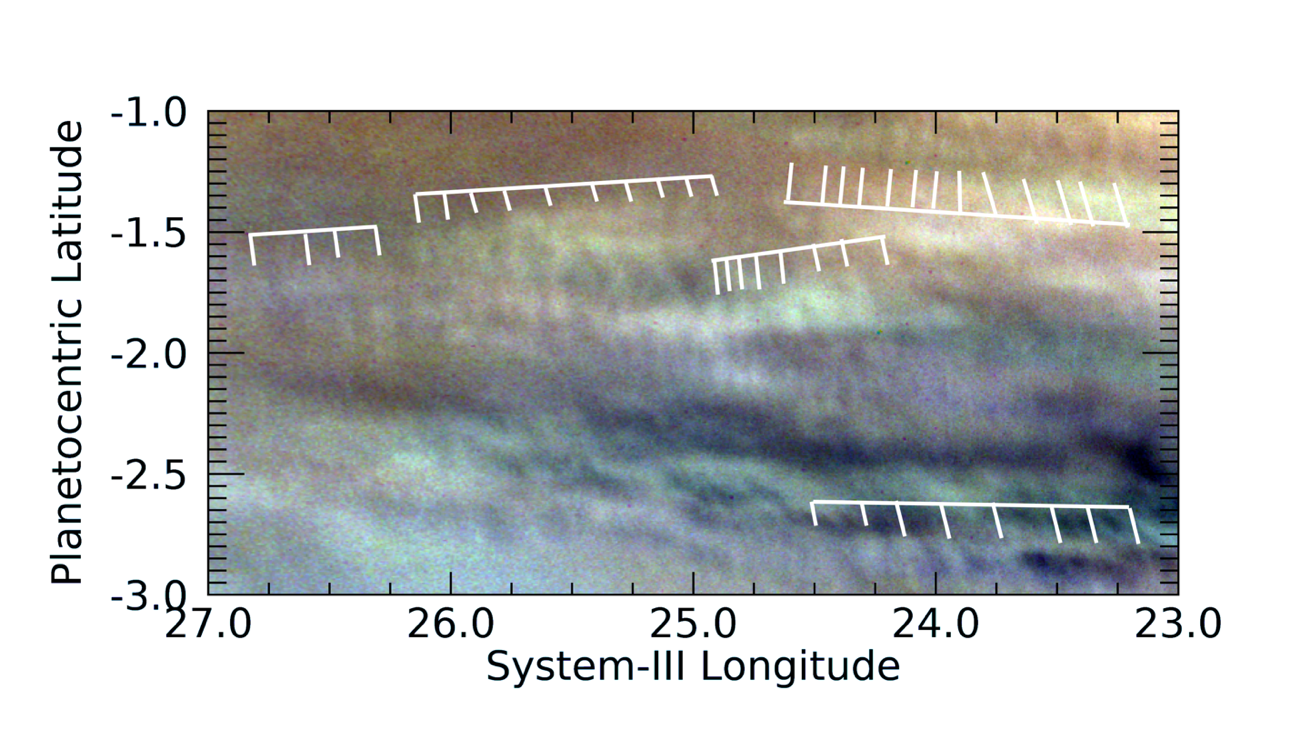


Figure PJ20_36. JNCE_2019149_20C00036_V01. Many, probably related, wave packets can be seen in this image, showing a region to the south of Figure PJ20_34b in the southern component of the Equatorial Zone.

SI2. Table of Measured Wave Properties

| PJ | Image | No. of Crests | Mean  System-III Longitude | Mean  Planeto-centric Latitude | Length(km) | Width(km) | Mean  Wave- length (km) | Tilt (deg.) |
| --- | --- | --- | --- | --- | --- | --- | --- | --- |
| 1 | 99 | 12 | 277.4 | 2.3 | 1443.0 | 172.2 | 131.2 | 99.2 |
| 3 | 109 | 3 | 6.6 | 25.6 | 294.5 | 243.2 | 147.2 | 81.6 |
| 3 | 109 | 4 | 5.6 | 30.6 | 923.5 | 669.6 | 307.8 | 76.7 |
| 3 | 109 | 3 | 7.0 | 29.7 | 566.5 | 747.2 | 283.3 | 68.2 |
| 3 | 109 | 3 | 5.1 | 23.1 | 723.7 | 436.0 | 361.9 | 69.1 |
| 3 | 111 | 18 | 8.9 | 2.0 | 1881.6 | 807.9 | 110.7 | 90.1 |
| 3 | 111 | 12 | 6.8 | 1.7 | 1159.1 | 249.2 | 105.4 | 69.0 |
| 3 | 111 | 7 | 6.4 | -5.2 | 999.1 | 373.7 | 166.5 | 98.2 |
| 3 | 114 | 6 | 18.8 | -15.1 | 974.2 | 238.0 | 194.8 | 100.0 |
| 3 | 114 | 17 | 16.5 | -16.1 | 2647.8 | 331.1 | 165.5 | 93.1 |
| 3 | 114 | 7 | 18.9 | -15.6 | 921.3 | 789.7 | 153.6 | 86.0 |
| 3 | 114 | 12 | 18.6 | -17.2 | 2169.9 | 694.0 | 197.3 | 81.0 |
| 4 | 102 | 3 | 88.3 | 9.7 | 902.2 | 1256.1 | 451.1 | 107.8 |
| 4 | 103 | 3 | 94.6 | 14.4 | 308.8 | 318.9 | 154.4 | 61.0 |
| 4 | 103 | 3 | 94.3 | 13.8 | 324.7 | 502.3 | 162.4 | 71.7 |
| 4 | 103 | 3 | 93.5 | 14.4 | 403.8 | 608.4 | 201.9 | 84.3 |
| 4 | 103 | 3 | 93.7 | 13.3 | 405.1 | 382.6 | 202.6 | 127.9 |
| 4 | 103 | 3 | 94.5 | 12.9 | 425.5 | 484.9 | 212.8 | 62.6 |
| 4 | 103 | 26 | 95.1 | 12.0 | 912.8 | 74.9 | 36.5 | 50.4 |
| 4 | 103 | 6 | 94.0 | 11.5 | 145.5 | 34.2 | 29.1 | 75.2 |
| 4 | 104 | 15 | 96.8 | 1.8 | 2541.5 | 143.4 | 181.5 | 91.1 |
| 4 | 104 | 9 | 96.1 | 0.7 | 1321.3 | 398.4 | 165.2 | 83.9 |
| 4 | 104 | 10 | 95.2 | -0.8 | 1409.9 | 387.9 | 156.7 | 101.8 |
| 4 | 104 | 5 | 96.3 | 3.0 | 1704.5 | 1225.7 | 426.1 | 25.1 |
| 5 | 107 | 3 | 9.0 | 0.1 | 229.1 | 868.1 | 114.5 | 123.3 |
| 5 | 107 | 6 | 8.0 | 0.3 | 1387.1 | 832.3 | 277.4 | 94.5 |
| 5 | 107 | 4 | 6.0 | 0.4 | 489.8 | 240.1 | 163.3 | 68.5 |
| 5 | 107 | 5 | 5.7 | -0.1 | 426.4 | 249.5 | 106.6 | 101.4 |
| 5 | 107 | 5 | 6.3 | -1.1 | 884.9 | 264.5 | 221.2 | 80.6 |
| 5 | 108 | 4 | 12.9 | -17.5 | 693.0 | 986.5 | 231.1 | 98.4 |
| 6 | 115 | 14 | 321.4 | 2.6 | 1188.4 | 137.8 | 91.4 | 116.9 |
| 6 | 115 | 9 | 322.6 | 2.0 | 705.2 | 196.0 | 88.1 | 104.6 |
| 6 | 115 | 6 | 321.0 | 1.8 | 338.4 | 301.5 | 67.7 | 109.7 |
| 6 | 115 | 5 | 322.0 | 1.5 | 604.7 | 508.9 | 151.2 | 95.3 |
| 6 | 115 | 4 | 321.3 | 1.2 | 426.3 | 422.4 | 142.1 | 78.0 |
| 6 | 115 | 4 | 321.9 | 1.0 | 335.5 | 444.4 | 111.8 | 76.6 |
| PJ | Image | No. of Crests | Mean  System-III Longitude | Mean  Planeto-centric Latitude | Length(km) | Width(km) | Mean Wave- length (km) | Tilt (deg.) |
| 6 | 115 | 6 | 322.5 | 0.4 | 492.8 | 250.4 | 98.6 | 88.5 |
| 6 | 115 | 3 | 321.2 | 0.2 | 364.0 | 193.3 | 182.0 | 96.0 |
| 6 | 115 | 34 | 323.8 | -4.4 | 5177.0 | 253.4 | 156.9 | 89.8 |
| 7 | 57 | 25 | 231.5 | 3.4 | 2149.8 | 155.7 | 89.6 | 97.5 |
| 7 | 57 | 6 | 230.8 | 3.4 | 690.5 | 198.2 | 138.1 | 59.4 |
| 7 | 57 | 6 | 232.0 | 2.8 | 1110.3 | 348.9 | 222.1 | 50.4 |
| 7 | 59 | 7 | 238.3 | -15.8 | 432.6 | 233.9 | 72.1 | 77.7 |
| 7 | 59 | 9 | 237.8 | -15.9 | 628.0 | 118.0 | 78.5 | 92.2 |
| 8 | 116 | 16 | 144.8 | -2.3 | 1509.7 | 243.6 | 100.6 | 127.2 |
| 8 | 116 | 17 | 143.5 | -3.2 | 1787.7 | 179.2 | 111.7 | 75.8 |
| 8 | 116 | 21 | 144.0 | -4.9 | 1987.8 | 295.5 | 99.4 | 84.3 |
| 8 | 116 | 21 | 144.1 | -5.1 | 2069.4 | 203.9 | 103.5 | 56.2 |
| 9 | 85 | 6 | 53.8 | 2.3 | 924.4 | 139.0 | 184.9 | 84.6 |
| 9 | 88 | 25 | 53.4 | -3.7 | 5833.8 | 282.4 | 243.1 | 77.8 |
| 10 | 28 | 24 | 119.6 | 2.9 | 3474.3 | 216.5 | 151.1 | 75.5 |
| 10 | 30 | 4 | 127.5 | -0.9 | 604.2 | 427.6 | 201.4 | 83.7 |
| 10 | 30 | 7 | 124.9 | -1.6 | 848.8 | 310.5 | 141.5 | 101.4 |
| 10 | 30 | 6 | 125.3 | -2.2 | 852.5 | 190.6 | 170.5 | 114.1 |
| 10 | 30 | 8 | 124.1 | -2.2 | 940.9 | 204.2 | 134.4 | 95.9 |
| 10 | 30 | 6 | 126.6 | -3.8 | 937.6 | 167.8 | 187.5 | 109.0 |
| 10 | 30 | 22 | 124.5 | -3.3 | 3024.1 | 354.5 | 144.0 | 115.4 |
| 10 | 30 | 10 | 120.5 | 2.9 | 1449.3 | 170.5 | 161.0 | 81.6 |
| 11 | 20 | 4 | 38.7 | 3.5 | 943.7 | 647.3 | 314.6 | 88.0 |
| 11 | 20 | 27 | 34.8 | 2.6 | 4408.6 | 682.0 | 169.6 | 98.6 |
| 11 | 20 | 7 | 35.7 | 1.7 | 1764.9 | 309.6 | 294.1 | 113.9 |
| 11 | 20 | 9 | 32.0 | 1.8 | 1491.3 | 265.5 | 186.4 | 96.3 |
| 11 | 20 | 8 | 38.1 | 0.3 | 3829.3 | 1125.4 | 547.0 | 40.2 |
| 11 | 20 | 13 | 38.3 | -3.5 | 2117.7 | 317.4 | 176.5 | 108.5 |
| 11 | 20 | 8 | 35.3 | -3.4 | 2138.7 | 218.7 | 305.5 | 96.5 |
| 12 | 87 | 7 | 293.4 | 21.2 | 2909.2 | 255.5 | 484.9 | 102.6 |
| 12 | 87 | 7 | 293.2 | 19.9 | 1666.1 | 223.0 | 277.7 | 92.4 |
| 12 | 87 | 11 | 289.0 | 30.1 | 3785.3 | 273.6 | 378.529 | 95.1 |
| 12 | 90 | 24 | 299.8 | -1.4 | 1128.0 | 421.2 | 49.044 | 54.9 |
| 12 | 90 | 27 | 301.9 | -1.9 | 4751.8 | 441.8 | 182.8 | 126.7 |
| 12 | 90 | 19 | 300.8 | -2.9 | 2448.5 | 319.9 | 136.0 | 113.5 |
| 12 | 90 | 7 | 302.9 | -3.0 | 1338.2 | 164.9 | 223.0 | 87.4 |
| 12 | 90 | 20 | 302.7 | -4.1 | 1613.8 | 238.8 | 84.9 | 97.0 |
| 12 | 90 | 8 | 301.7 | -4.3 | 637.9 | 191.1 | 91.1 | 113.7 |
| 12 | 90 | 4 | 301.0 | -4.4 | 320.6 | 362.3 | 106.9 | 140.1 |
| PJ | Image | No. of Crests | Mean  System-III Longitude | Mean  Planeto-centric Latitude | Length(km) | Width(km) | Mean Wave- length (km) | Tilt (deg.) |
| 12 | 90 | 19 | 299.7 | 1.5 | 2423.7 | 185.1 | 134.7 | 74.1 |
| 12 | 90 | 12 | 300.4 | -5.0 | 339.9 | 62.2 | 30.9 | 88.2 |
| 12 | 86 | 4 | 293.7 | 33.0 | 391.6 | 279.8 | 130.5 | 108.5 |
| 12 | 86 | 4 | 294.3 | 32.4 | 129.5 | 45.5 | 43.1 | 71.3 |
| 12 | 86 | 3 | 294.1 | 32.4 | 98.9 | 35.2 | 49.5 | 93.5 |
| 12 | 86 | 4 | 294.0 | 32.0 | 333.5 | 162.8 | 111.2 | 73.7 |
| 12 | 86 | 5 | 289.2 | 34.2 | 543.4 | 588.2 | 135.9 | 118.3 |
| 12 | 86 | 4 | 289.9 | 33.3 | 286.0 | 390.8 | 95.3 | 100.1 |
| 12 | 86 | 4 | 288.5 | 32.5 | 413.0 | 303.7 | 137.7 | 113.3 |
| 12 | 86 | 4 | 289.0 | 32.2 | 310.8 | 1924.2 | 103.6 | 112.2 |
| 13 | 35 | 6 | 215.3 | -1.6 | 1858.2 | 270.6 | 371.6 | 110.4 |
| 13 | 35 | 4 | 219.3 | -1.6 | 574.8 | 513.4 | 191.6 | 95.5 |
| 13 | 35 | 11 | 220.2 | -3.5 | 2421.9 | 387.2 | 242.2 | 76.4 |
| 13 | 35 | 13 | 216.6 | -4.5 | 1777.0 | 548.0 | 148.1 | 117.9 |
| 13 | 35 | 10 | 214.6 | -4.7 | 2421.2 | 627.1 | 269.0 | 81.0 |
| 13 | 35 | 5 | 221.6 | -4.5 | 875.3 | 339.6 | 218.8 | 109.9 |
| 13 | 35 | 9 | 220.383 | -5.0 | 1152.5 | 449.5 | 144.1 | 105.7 |
| 13 | 35 | 6 | 217.954 | -4.7 | 1171.0 | 456.8 | 234.2 | 123.7 |
| 13 | 35 | 10 | 216.356 | -4.7 | 1561.1 | 394.5 | 173.5 | 127.1 |
| 13 | 35 | 11 | 217.948 | 1.9 | 2298.3 | 569.7 | 229.8 | 68.5 |
| 13 | 35 | 8 | 216.275 | 3.0 | 1527.4 | 728.1 | 218.2 | 71.5 |
| 13 | 36 | 6 | 222.974 | -19.7 | 1109.9 | 75.5 | 222.0 | 79.6 |
| 13 | 36 | 4 | 221.939 | -19.3 | 561.6 | 61.6 | 187.2 | 96.4 |
| 14 | 25 | 5 | 251.918 | 17.3 | 650.7 | 587.9 | 162.7 | 94.8 |
| 14 | 25 | 3 | 250.69 | 17.6 | 421.5 | 632.1 | 210.7 | 93.6 |
| 14 | 25 | 4 | 252.245 | 11.9 | 324.7 | 499.6 | 108.2 | 70.1 |
| 14 | 25 | 3 | 251.308 | 12.2 | 447.9 | 202.1 | 223.9 | 60.6 |
| 14 | 25 | 4 | 255.706 | 6.4 | 982.4 | 2170.5 | 327.5 | 103.7 |
| 14 | 25 | 4 | 253.429 | 6.8 | 1454.9 | 1030.7 | 485.0 | 70.7 |
| 14 | 28 | 6 | 262.072 | -3.8 | 1517.0 | 256.3 | 303.4 | 93.5 |
| 14 | 28 | 4 | 259.751 | -3.7 | 982.4 | 263.5 | 327.5 | 101.8 |
| 14 | 28 | 10 | 260.933 | -4.6 | 1290.5 | 148.1 | 143.4 | 90.4 |
| 14 | 28 | 26 | 258.247 | -4.5 | 4657.9 | 199.4 | 186.3 | 105.6 |
| 14 | 28 | 12 | 258.918 | -5.1 | 2566.7 | 188.0 | 233.3 | 114.4 |
| 14 | 28 | 10 | 256.575 | -5.4 | 2060.6 | 213.2 | 229.0 | 112.4 |
| 14 | 28 | 15 | 256.225 | -6.031 | 1592.8 | 177.7 | 113.8 | 111.9 |
| 14 | 28 | 9 | 256.585 | 0.19 | 1260.0 | 415.2 | 157.5 | 60.2 |
| 14 | 28 | 8 | 258.039 | -0.628 | 1614.5 | 408.7 | 230.6 | 89.5 |
| 14 | 28 | 7 | 259.961 | -0.921 | 1652.8 | 543.3 | 275.5 | 118.6 |
| PJ | Image | No. of Crests | Mean  System-III Longitude | Mean  Planeto-centric Latitude | Length(km) | Width(km) | Mean  Wave- length (km) | Tilt (deg.) |
| 14 | 28 | 5 | 259.663 | -1.8 | 1126.9 | 402.0 | 281.7 | 126.9 |
| 14 | 28 | 5 | 256.697 | -2.6 | 673.6 | 321.2 | 168.4 | 106.7 |
| 14 | 28 | 5 | 259.574 | -3.6 | 1361.6 | 524.4 | 340.4 | 104.4 |
| 14 | 28 | 5 | 256.579 | -3.3 | 933.3 | 273.6 | 233.3 | 128.0 |
| 15 | 24 | 9 | 331.914 | 22.7 | 960.2 | 220.5 | 120.0 | 135.2 |
| 15 | 29 | 6 | 347.466 | -2.0 | 646.7 | 138.1 | 129.3 | 120.0 |
| 15 | 29 | 4 | 346.416 | -2.7 | 501.6 | 239.4 | 167.2 | 125.6 |
| 15 | 29 | 5 | 347.613 | -2.4 | 874.6 | 238.8 | 218.6 | 127.5 |
| 15 | 29 | 5 | 348.354 | -2.0 | 673.4 | 458.6 | 168.4 | 86.9 |
| 15 | 29 | 23 | 347.087 | -2.2 | 2411.4 | 439.5 | 109.6 | 110.6 |
| 16 | 19 | 14 | 259.2 | -1.5 | 2504.5 | 478.9 | 192.7 | 110.9 |
| 16 | 19 | 10 | 256.577 | -1.5 | 984.5 | 205.0 | 109.4 | 121.1 |
| 16 | 19 | 20 | 257.432 | -2.3 | 2921.0 | 342.5 | 153.7 | 115.7 |
| 16 | 19 | 7 | 259.324 | -2.4 | 1213.7 | 440.9 | 202.3 | 143.0 |
| 16 | 19 | 9 | 259.717 | -3.9 | 1180.0 | 285.4 | 147.5 | 79.5 |
| 16 | 19 | 7 | 257.911 | -4.4 | 1094.1 | 299.3 | 182.4 | 91.5 |
| 16 | 19 | 6 | 256.964 | -4.3 | 833.3 | 195.8 | 166.7 | 99.9 |
| 17 | 26 | 4 | 179.208 | 4.3 | 1122.8 | 936.2 | 374.3 | 59.4 |
| 17 | 26 | 3 | 177.695 | 3.8 | 674.7 | 702.4 | 337.4 | 64.4 |
| 17 | 26 | 3 | 183.029 | 1.3 | 737.1 | 1246.4 | 368.6 | 85.6 |
| 17 | 26 | 4 | 178.393 | 1.1 | 2952.4 | 1236.4 | 984.1 | 108.1 |
| 17 | 26 | 3 | 181.868 | 0.1 | 1221.9 | 1557.6 | 611.0 | 124.4 |
| 17 | 26 | 6 | 176.48 | -1.8 | 2521.5 | 432.0 | 504.3 | 71.0 |
| 17 | 26 | 3 | 174.442 | -2.5 | 442.0 | 557.9 | 221.0 | 92.3 |
| 17 | 26 | 12 | 167.233 | -4.8 | 2988.8 | 290.0 | 271.7 | 108.0 |
| 17 | 26 | 6 | 171.684 | 2.8 | 993.5 | 436.9 | 198.7 | 68.8 |
| 17 | 24 | 4 | 165.336 | 12.0 | 1640.6 | 1148.6 | 546.9 | 151.3 |
| 18 | 34 | 13 | 231.486 | 16.3 | 1479.7 | 253.3 | 123.3 | 123.3 |
| 18 | 38 | 2 | 253.097 | 4.5 | 336.9 | 990.8 | 336.9 | 85.0 |
| 18 | 38 | 4 | 250.31 | 4.4 | 1496.1 | 532.8 | 498.7 | 46.0 |
| 18 | 38 | 5 | 248.654 | 4.1 | 739.6 | 679.9 | 184.9 | 89.4 |
| 18 | 38 | 6 | 252.512 | 2.7 | 1236.6 | 1510.1 | 247.3 | 96.2 |
| 18 | 38 | 3 | 257.468 | 2.3 | 1143.6 | 1282.5 | 571.8 | 56.1 |
| 18 | 38 | 4 | 255.324 | 1.7 | 1792.6 | 955.6 | 597.5 | 109.6 |
| 18 | 38 | 3 | 251.435 | 0.6 | 942.5 | 610.1 | 471.2 | 113.8 |
| 18 | 38 | 14 | 248.909 | -0.2 | 1802.4 | 177.6 | 138.7 | 75.3 |
| 18 | 39 | 8 | 261.87 | 2.0 | 4251.8 | 2824.4 | 607.4 | 62.1 |
| 18 | 39 | 4 | 261.65 | 0.5 | 2291.3 | 1374.9 | 763.8 | 57.5 |
| 18 | 39 | 10 | 255.183 | -3.9 | 2556.7 | 344.9 | 284.1 | 126.1 |
| PJ | Image | No. of Crests | Mean  System-III Longitude | Mean  Planeto-centric Latitude | Length(km) | Width(km) | Mean  Wave- length (km) | Tilt (deg.) |
| 18 | 39 | 8 | 255.9 | -4.4 | 944.9 | 190.7 | 135.0 | 142.1 |
| 20 | 26 | 5 | 4.9 | 51.2 | 565.4 | 121.2 | 141.3 | 117.7 |
| 20 | 26 | 10 | 4.8 | 51.1 | 756.8 | 85.3 | 84.1 | 123.5 |
| 20 | 26 | 5 | -1.2 | 47.7 | 469.0 | 123.8 | 117.3 | 60.5 |
| 20 | 33 | 2 | 22.4 | 6.3 | 297.4 | 3411.7 | 297.4 | 111.5 |
| 20 | 34 | 18 | 20.6 | 3.5 | 1745.0 | 340.6 | 102.6 | 102.3 |
| 20 | 34 | 10 | 22.2 | 2.3 | 1343.8 | 371.9 | 149.3 | 98.2 |
| 20 | 34 | 4 | 23.0 | 1.9 | 444.6 | 357.7 | 148.2 | 138.0 |
| 20 | 34 | 3 | 20.0 | 2.7 | 242.4 | 3239.2 | 121.2 | 161.5 |
| 20 | 34 | 27 | 21.7 | 0.1 | 4547.5 | 366.2 | 174.9 | 89.8 |
| 20 | 34 | 11 | 27.6 | 0.8 | 2394.4 | 232.1 | 239.4 | 75.9 |
| 20 | 34 | 4 | 29.8 | 0.5 | 591.2 | 404.3 | 197.1 | 92.1 |
| 20 | 34 | 3 | 25.2 | 0.6 | 580.3 | 418.5 | 290.2 | 76.4 |
| 20 | 34 | 21 | 20.1 | 3.3 | 1741.8 | 344.5 | 87.1 | 102.7 |
| 20 | 36 | 6 | 24.1 | -3.1 | 826.2 | 255.1 | 165.2 | 117.2 |
| 20 | 36 | 10 | 24.2 | -3.5 | 1115.6 | 191.3 | 124.0 | 97.6 |
| 20 | 36 | 7 | 25.5 | -3.8 | 1076.0 | 275.6 | 179.3 | 72.3 |
| 20 | 36 | 14 | 23.8 | -4.0 | 1809.4 | 261.7 | 139.2 | 115.8 |
| 20 | 36 | 6 | 27.2 | -4.3 | 1245.4 | 513.1 | 249.1 | 142.7 |
| 20 | 36 | 11 | 28.5 | -4.6 | 2102.1 | 209.8 | 210.2 | 71.0 |
| 20 | 36 | 13 | 23.9 | -1.1 | 1616.7 | 176.7 | 134.7 | 85.7 |
| 20 | 36 | 10 | 25.5 | -1.5 | 1557.7 | 194.8 | 173.1 | 105.4 |
| 20 | 36 | 4 | 26.5 | -1.8 | 645.8 | 357.1 | 215.3 | 107.0 |
| 20 | 36 | 8 | 24.5 | -1.8 | 987.2 | 227.2 | 141.0 | 106.4 |
| 20 | 36 | 8 | 23.7 | -2.9 | 1396.8 | 239.9 | 199.5 | 114.8 |

Notes: The term ‘wavelength’ refers to the distance between wave crests or discrete clouds. The tilt angle is defined as 0º at the eastward direction of the wave packet to 180º in the westward direction of the wave packet. Thus, wave crests oriented more-or-less orthogonally to the direction of the wave packet, such as those shown in Figure 2 of the main article, have tilt angles close to 90º.

SI3. Figures of Measured Wave Properties


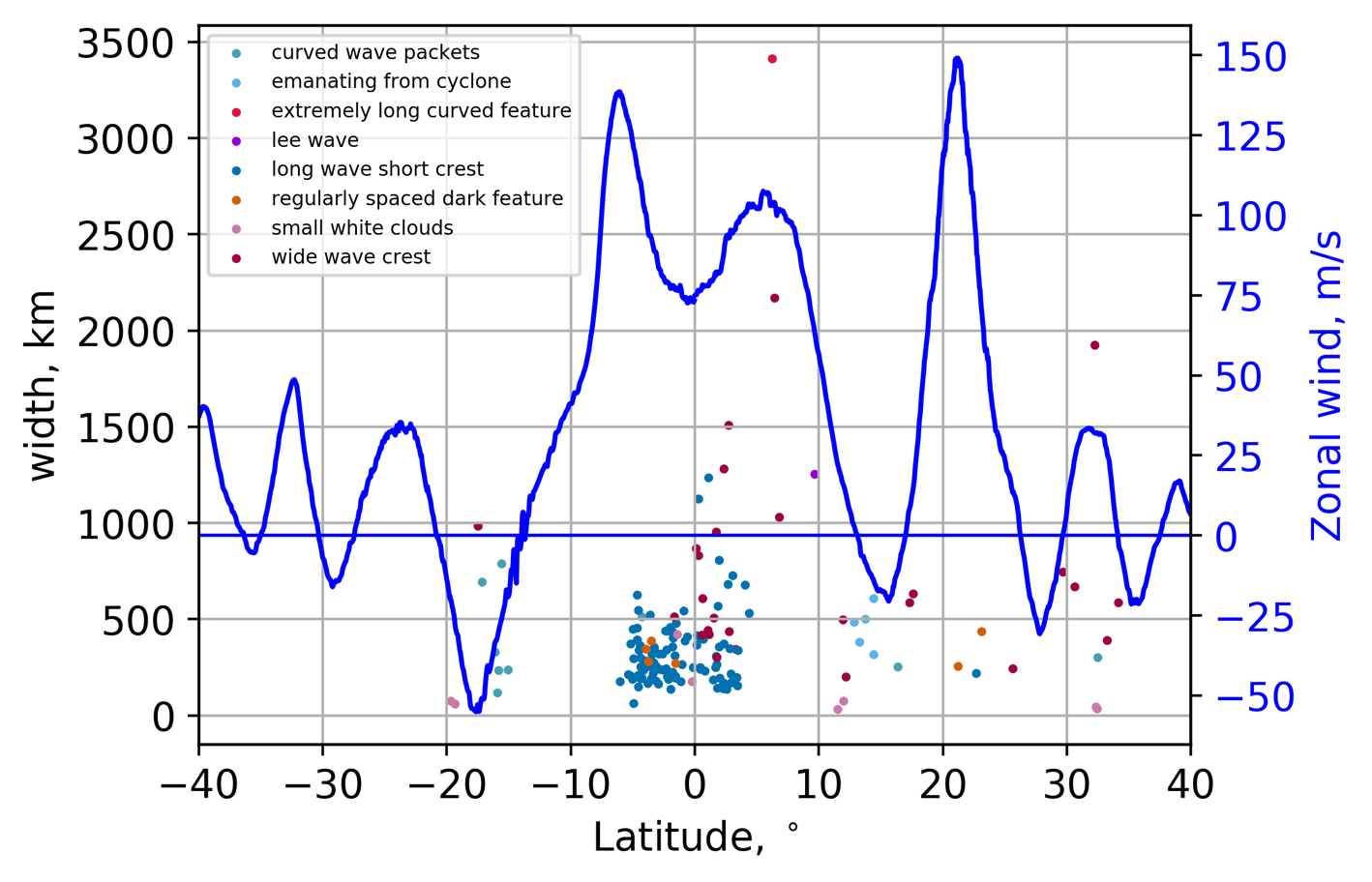


*Figure SI3-1. Plot of wave widths as a function of latitude. Zonal wind velocities are co-plotted in blue.*


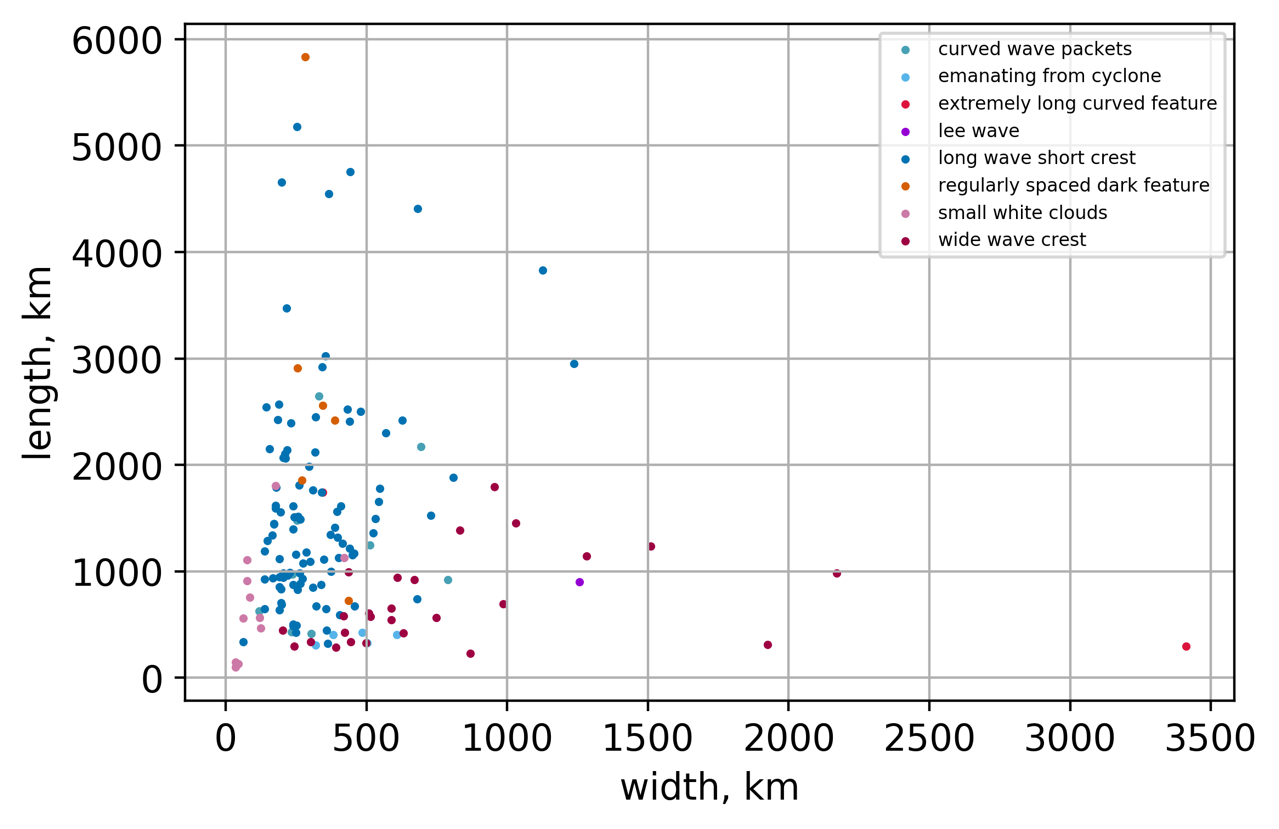


*Figure SI3-2. Plot of wave widths vs wavelengths. Although there is some overlap, most of the long wave packets with short crests (dark blue) and waves with wide crests (brownish-red) appear to be clustered independently from each other.*


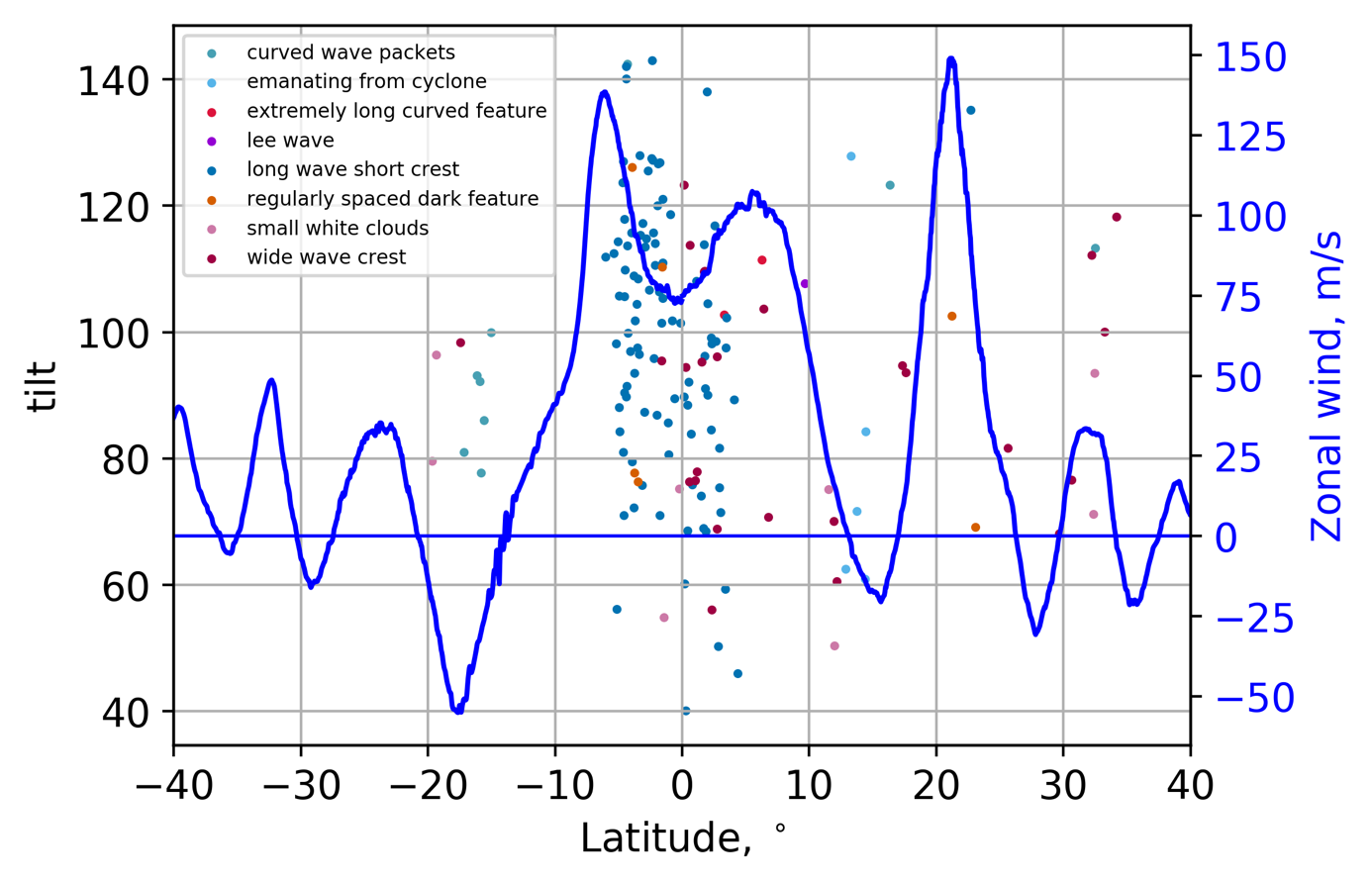


*Figure SI3-3. Plot of wave tilts in degrees as a function of latitude. Zonal wind velocity is co-plotted in blue.*
